# Predicting short- to long-term breast cancer risk from longitudinal mammographic screening history

**DOI:** 10.1038/s41523-025-00831-x

**Published:** 2025-10-29

**Authors:** Xin Wang, Tao Tan, Yuan Gao, Ruisheng Su, Jonas Teuwen, Jaap Kroes, Tianyu Zhang, Anna D’Angelo, Luyi Han, Caroline A. Drukker, Marjanka K. Schmidt, Regina Beets-Tan, Nico Karssemeijer, Ritse Mann

**Affiliations:** 1 Department of Radiology, The Netherlands Cancer Institute, 1066 CX Amsterdam, The Netherlands; 2 GROW School for Oncology and Development Biology, Maastricht University, P. O. Box 616, 6200 MD Maastricht, The Netherlands; 3 Department of Diagnostic Imaging, Radboud University Medical Center, 6525 GA Nijmegen, The Netherlands; 4 Faculty of Applied Sciences, Macao Polytechnic University, 999078 Macao, China; 5 Erasmus Medical Center, Erasmus University, 3015 GD Rotterdam, The Netherlands; 6 Department of Radiation Oncology, The Netherlands Cancer Institute, 1066 CX Amsterdam, The Netherlands; 7 ScreenPoint Medical, Nijmegen, 6525 EC The Netherlands; 8 Dipartimento di Diagnostica per Immagini, Radioterapia Oncologica ed Ematologia, Fondazione Policlinico Universitario A. Gemelli, IRCCS, 00168 Rome, Italy; 9 Department of Surgical Oncology, The Netherlands Cancer Institute, 1066 CX Amsterdam, The Netherlands; 10 Division of Molecular Pathology, The Netherlands Cancer Institute, 1066 CX Amsterdam, The Netherlands; 11 Department of Epidemiology, The Netherlands Cancer Institute, 1066 CX Amsterdam, The Netherlands; 12 Department of Medical Imaging, Radboud University Medical Center, 6500 HB Nijmegen, The Netherlands

**Keywords:** Breast cancer, Population screening, Translational research, Cancer screening

## Abstract

Breast cancer (BC) risk assessment aims to enhance individualized screening and prevention strategies. While recent deep learning (DL) models based on mammography have shown promise in short-term risk prediction, they primarily rely on single-time-point (STP) exams, ignoring temporal changes in breast tissue from sequence exams. We present the Multi-Time Point Breast Cancer Risk Model (MTP-BCR), a novel DL approach that integrates traditional risk factors and longitudinal mammography data to capture subtle tissue changes indicative of future malignancy. Using a large in-house dataset with 171,168 mammograms from 9133 women, MTP-BCR achieved superior performance in 10-year risk prediction, with an AUC of 0.80 (95% CI, 0.78–0.82) at the patient level, outperforming STP-based and traditional risk models. External validation on the CSAW-CC dataset confirmed its robustness. Further analysis demonstrates the advantages of the MTP-BCR method in diverse populations. MTP-BCR also excels in risk stratification and offers heatmaps to enhance clinical interpretability.

## Introduction

Breast cancer (BC) is one of the most common cancers in the world and is the cause of a large fraction of cancer-related mortality among women^1,2^. Age-based population-level BC screening programs have been implemented to detect breast tumors at an early stage and reduce BC-specific mortality^3–7^. However, the broad adoption of mammographic screening has led to concerns regarding the high cost of imaging, false positives, and overdiagnoses, which explains the strong controversy over screening^8,9^. In response to these challenges, “personalized” BC screening regimens are advocated based on the individual woman’s future risk of BC, which follows from demographic and genetic information, exposure to endogenous and exogenous risk factors, and also medical imaging^10–12^. Current BC risk assessment models are designed to be sensitive to the high-risk population who could benefit from more aggressive screening and prevention. At the same time, these models may advocate less frequent screening for the low-risk population to reduce the harm and cost of screening^13,14^.

Based on the timespan for BC prediction, risk models can be divided into short- and long-term risk models. Short-term risk models can be used to guide physicians in selecting supplemental screening for high-risk women, while long-term risk prediction helps to determine risk-based screening regimens and eligibility for preventive treatment for low- and high-risk individuals^15^. Traditional risk models, such as Tyrer-Cuzick^11^, CANRISK^16^, BCRAT^12^, and BCSC^17^, primarily focus on long-term risk estimates. Performances in clinical practice remain modest, as they lack sensitivity to short/middle-term cancer risk variation due to the limited incorporation of time-varying risk factors and individual-specific risk factors (detailed imaging characteristics) beyond breast density. In recent years, deep learning (DL) methods, combining screening mammograms with detailed risk factors outperform the clinically adopted traditional models in five-year BC risk prediction^7,14,18–27^. However, due to the lack of a long-term longitudinal screening mammogram dataset, the potential of image-based DL methods for longer-term (e.g., 10 years) BC risk prediction has been less explored.

More importantly, the most recent DL-based models directly learn the risk prediction from single-time point (STP) images or exams without considering historical reference^7,18,21^. We hypothesize that learning the breast tissue changes may facilitate BC risk prediction. In clinical practice, radiologists routinely compare mammography exams to identify developing abnormalities. Therefore, beyond learning risk features (e.g., breast density) from STP imaging, multi-time point learning may also be helpful in discovering the underlying dynamics of the risk pattern for BC development^22,28^. Besides, according to current BC guidelines, it is recommended that women who have undergone breast-conserving therapy for early-stage BC should continue with screening mammography^29,30^. Hence, in real-world clinical practice, the consideration of both primary and recurring breast cancers in risk prediction models becomes imperative. While existing image-based risk models tend to emphasize primary BC, understanding the dynamics of recurrence risk is equally crucial for developing personalized screening and prevention strategies. Our study aims to estimate both types of risks, reflecting real-world clinical scenarios. To achieve this, it is essential to incorporate available prior tumor information and prognostic risk factors into the prediction models, as this information is crucial for accurately assessing recurrence risk^29^. This comprehensive approach enables a more complete assessment of long-term BC risk, ultimately improving patient care.

Besides, the interpretability of medical AI risk models remains a challenge. The comprehension of the underlying tissue changes behind predicted outputs is crucial for gaining clinical acceptance. How to endow an existing risk model with explainability of the underlying reasoning remains the boundary to explore. Apart from being similar to what an actual radiologist does when searching for the signs of BC risk^31,32^, AI models should reasonably show radiologists more details during inference^33^. Most recent studies focusing on patient-level risk prediction aim to reference non-lesion imaging biomarkers, such as the density or pattern of the background parenchyma^34^, which could improve the interpretability. However, their methods do not provide location-specific risk at the breast level. Improving these specific predictions and visualizations is also essential to enhance the interpretability of the model, enabling physicians to grasp the model’s decision-making more effectively. Additionally, more detailed risk information can guide physicians to focus on changing areas of the breast, facilitating more targeted examinations and personalized prevention strategies. Thus, an ideal risk model should not only stratify high-risk groups but also direct the attention of medical practitioners to evolving areas in the breast at an earlier stage.

We develop the multi-time-points breast cancer risk (MTP-BCR) model, an end-to-end model that estimates the long-term future BC risk based on changes in breast tissue, considering both primary and recurring breast cancers. Our model leverages historical and current exams from the longitudinal clinical mammogram datasets. Additionally, our method supports both BC risk prediction at the patient level and at the unilateral breast level. Unilateral breast level BC risk could consider potential heterogeneity in risk factors and cancer occurrence between the bilateral breasts (e.g., imaging characteristics or anatomical variations, which may influence cancer development in each breast independently). To enhance the interpretability of our model, we highlight suspicious areas in a longitudinal test dataset using the model’s heatmaps, which may also illustrate the attention consistency of our model and improve its interpretability. The results imply that our model obtains a remarkable performance compared to clinical traditional risk (primary) and recurrence risk models and other single-time point mammogram-based DL methods among various scenarios.

## Results

### Overview of study design

**Fig. 1:**
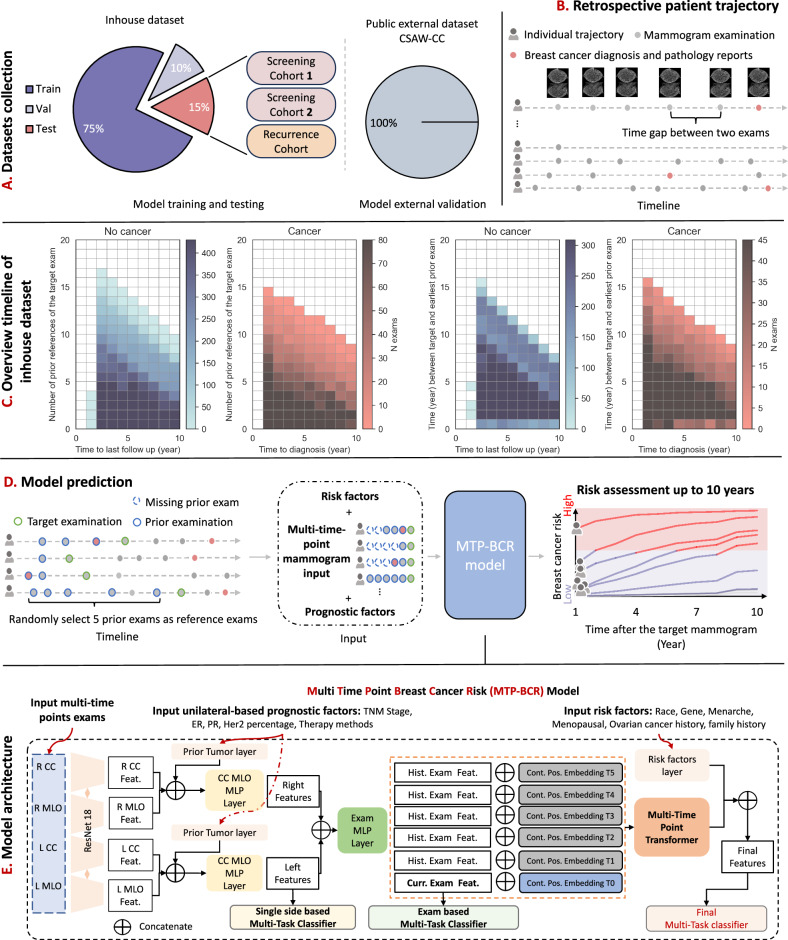
Overview of the study design. **A** In-house and public datasets collection for model building and validation. We build our model based on the in-house dataset. To further evaluate the model’s performance on two screening cohorts, a recurrence cohort, and a public dataset. **B** A schematic diagram of a retrospective patient trajectory. The diagram simulates timelines of retrospective patient trajectories for the in-house dataset. Each grey dot represents a mammogram examination, and red dots mean the diagnostic examination with pathology results. **C** Overview of retrospective patients’ trajectory for the in-house dataset. In the first heatmap, we plot the distribution of examinations in terms of the number of prior reference examinations and the time to last negative follow-up in the no cancer group or the time to diagnosis in the cancer group. In the second, we plot the distribution of examinations in terms of the time interval between the target and the earliest prior reference examinations and the time to last negative follow-up in the no cancer group or the time to diagnosis in the cancer group. The plot shows that the retrospective trajectory distributions of the cancer and no cancer groups are similar. **D** Schematic of the BC risk prediction by the MTP-BCR model. The target mammogram (the dot with a green circle) is the mammogram for which the risk is calculated. This can be any mammogram in the timeline of a woman. Prior mammograms are those obtained before the target mammogram, and up to five prior mammograms can be inputted (the grey or red dots with blue circles). The time to BC is the time between eventual cancer detection and the target mammogram, which can be up to 10 years. **E** The architecture of the proposed MTP-BCR model. (Detailed in the Method).

To investigate the risk development pattern, multiple examinations of the breasts over a certain span of time are required. Longitudinal screening mammograms provide this data. In this study, we collect both in-house and public longitudinal datasets, as depicted in Fig. 1A. The datasets consist of retrospective trajectories from each woman, recorded at the hospital, with at least one year of follow-up data (as illustrated in the schematic representation, Fig. 1B). As shown in Fig. 1C, for the in-house dataset, groups of cancer and no cancer retrospective trajectory distributions in terms of the number of prior references and the time interval are similar. The retrospective trajectory distributions of the primary and the recurrence groups are also provided in Supplementary Fig. 1.

Like radiologists, who typically identify developing abnormalities by looking at changes in longitudinal exams, we propose a novel end-to-end multi-time point network, MTP-BCR, leveraging longitudinal mammograms and medical records to capture the features related to increased BC risk. For the risk estimation task, the risk model integrates the reference and target examinations to predict future risk after the date of the target examination (Fig. 1D). The target mammogram represents the specific mammogram for which the risk is calculated, which can be any mammogram in a woman’s timeline. Prior reference mammograms refer to those obtained before the target mammogram, with 0 up to 5 prior mammograms taken into consideration. This design allows the model to extract both static breast cancer risk features from single-time-point inputs and dynamic tissue changes from multiple time-point mammograms when available. The time to BC is measured as the duration between eventual cancer detection and the target mammogram.

Our objective is to accurately predict short- to long-term BC risk. To achieve this, unlike traditional STP-based methods, which rely solely on data from a single exam, we propose a multi-time point transformer to combine static and dynamic risk features across “multiple time points” learning. This strategy mimics practical clinical scenarios where longitudinal data is available, enabling the model to capture temporal changes that STP methods might miss, thus facilitating short- to long-term BC risk prediction. In addition, considering the specific breast level risk prediction, our MTP-BCR integrates “multi-level” learning, which allows for a more comprehensive understanding of risk at different levels of granularity. Furthermore, the “multi-task” learning method is designed to leverage clinical prior knowledge, including radiologic features and risk factors, to explicitly guide and constrain the learning process. This approach enhances the model’s ability to capture risk-related features more effectively, improving its overall predictive accuracy.

As illustrated in Fig. 1E, our model first extracts static risk features from a single time-point exam and prior medical records, utilizing a multi-level approach that considers both breast-specific and patient-level risks. Furthermore, the model incorporates a multi-time-point fusion strategy, selecting features from up to five historical exams prior to the current exam. By integrating these dynamic features with patient-specific risk factors, our model offers a more accurate prediction of 10-year BC risk, addressing both short- and long-term concerns. Further details regarding the architectural details, contents of the medical records, and risk factors are elaborated in the methodology section.

### Datasets for model training and evaluation

Table 1 shows the in-house dataset consisting of the 34,749 examinations in the cancer-free group of women without BC and at least 1 year of negative follow-up, and 8023 exams of women who were diagnosed with BC within 10 years in the cases group. The mean age is 53.77 (11.79) and 56.18 (11.68) for cancer-free group and cases groups, respectively^35,36^. Additionally, 35.68 and 31.95% of the cancer-free and case groups have dense breasts (ACR 3 and 4). The percentage of the personal history of BC is 50.94% for the cancer-free group and 53.30% for the cases group. The proportion of different receptor types of tumors is shown in Supplementary Table 1, and the distribution is highly consistent with that reported in the previous study^37^.

**Table 1:** Characteristics of participants with up to 10 years of follow-up for the in-house dataset and the CSAW-CC dataset

	In-house dataset		
Characteristic	Cancer free,^a^ *N* = 34,769	Cases, ^a^ *N* = 8023	*P* value ^b^
Age, years, mean (SD)	53.77 (11.79)	56.18 (11.68)	<0.01
Age at menarche, years, mean (SD)	12.98 (3.81)	12.87 (1.40)	0.67
Menopausal status *n*/*N*(%)			<0.01
Pre-menopausal	5333 (15.34%)	944 (11.77%)	
Peri-menopausal & Unknow	17,825 (51.27%)	3566 (44.45%)	
Post-menopausal	11,611 (33.39%)	3513 (43.79%)	
Family history of breast cancer, *n*/*N*(%)	24,026 (69.10%)	6852 (85,40%)	<0.01
Family history of ovarian cancer, *n*/*N*(%)	2009 (5.78%)	510 (6.36%)	0.052
Breast density *n*/*N*(%)			<0.01
ACR 1	2250 (6.49%)	445 (5.63%)	
ACR 2	20,051 (57.83%)	4941 (62.51%)	
ACR 3	9385 (27.07%)	1845 (23.34%)	
ACR 4	2987 (8.61%)	673 (8.61%)	
Race *n*/*N*(%)			<0.01
White	10,133 (29.14%)	2133 (26.59%)	
African	135 (0.39%)	25 (0.31%)	
Asian	265 (0.76%)	31 (0.39%)	
Other Race	90 (0.26%)	16 (0.20%)	
Unknow	24,146 (69.45%)	5818 (72.52%)	
Gene mutation (BRCA1/2, Tp53, Chek2) *n*/*N*(%)	31 (0.09%)	251 (3.13%)	0.031
Personal history of breast cancer, *n*/*N*(%)	17,711 (50.94%)	4276 (53.30%)	<0.01
Personal history of ovarian cancer, *n*/*N*(%)	368 (1.06%)	138 (1.72%)	<0.01
Time to cancer, years *n*/*N*(%)			-
0–1	-	3742 (46.64%)	
1–2	-	719 (8.96%)	
2–3	-	691 (8.61%)	
3–4	-	593 (7.39%)	
4–5	-	525 (6.54%)	
5–6	-	451 (5.62%)	
6–7	-	426 (5.31%)	
7–8	-	351 (4.37%)	
8–9	-	292 (3.64%)	
9–10	-	233 (2.90%)	

	CSAW-CC dataset		
Characteristic	Cancer free,^a^ *N* = 22,871	Cases,^a^*N* = 1826	*P*value ^b^
Age range *n*/*N*(%)			<0.01
40–55	12,647 (55.30%)	737 (40.36%)	
>55	10,224 (44.70%)	1089 (59.64%)	
Percent mammographic density, mean (SD)	24.25 (15.04)	24.86 (14.95)	<0.01
Time to cancer *n*/*N*(%)			-
0–60 days	-	524 (28.70%)	
60 days–2 years	-	267 (14.62%)	
2–7 years	-	1035 (56.68%)	

^a^Subcohort of noncases with at least 1 year of negative follow-up. For cases, all incident cases diagnosed within 10 years are included.

^b^Wilcoxon rank-sum test; Pearson’s *x*^2^ test.

*SD* standard deviation.

For model training, the dataset is divided into training, validation, and test sets at the patient level. Detailed demographics of each set are given in Supplementary Table 2. The proposed model aims to handle multiple tasks, which encompass the detection of existing malignancies, future risk prediction of primary tumors, and prediction of tumor recurrence. This would facilitate implementation in an actual clinical BC screening program, where not only focusing on the stratification of the high-risk population is essential. In fact, the detection of existing malignancies can be considered an extremely short-term BC risk^38^. Furthermore, some of the model characteristics for these tasks of cancer detection and future risk prediction can be complementary^21^.

In this study, we compare our method with traditional and mammogram-based DL models. The traditional methods include the BCSC risk model (traditional-risk factors), retrospective BI-RADS scores of the radiologist (BI-RADS)^21^, a machine learning (SVM) based risk model (baseline-risk factors), and a machine learning-based BC recurrence risk prediction model (baseline-recurrence)^39^. The mammogram-based DL models include a single-time-point image-based baseline method (STP-Baseline), a single-time-point exam-transformer-based risk model (STP-transformer)^21^, and a single-time-point BC detection method (STP-detection)^32^. More detailed information is provided in the method part. For full leverage of mammogram examinations, we include all examinations with at least one year of screening follow-up.

#### Evaluation of different cohorts for risk stratification

To evaluate the model’s capability in longer-term BC risk prediction, we utilized two standard screening cohorts with distinct stratified aims: assessing future risk in patients with benign findings and identifying subtle risks in populations deemed low-risk by radiologists. We employ two screening test sets following the methodologies outlined in ref. ^26^, ^21^. The screening cohort 1 (normal or biopsy-negative screening group), comprises 5937 mammography exams involving 1236 patients. These exams were characterized by BI-RADS 1 and 2 scores, or other BI-RADS scores, but with benign biopsy results within 90 days from the screening date. This cohort helps assess how well the risk models can predict future risk, including for patients who have had a benign finding, thus supporting decisions about long-term monitoring and preventive care. The screening cohort 2 (the normal BI-RADS screening group: 5139 exams/1157 patients) only consists of the exams scored as BI-RADS 0, 1, and 2. This cohort evaluates the model’s ability to detect subtle risk factors in a population considered low-risk by radiologists, which could be crucial for refining screening practices and ensuring high-risk individuals are not overlooked. Note that, for the sake of maintaining comparability with BCSC methods, we also reported the results of both cohorts, which excluded examinations with a prior history of BC or those outside the age range of 35–74. Furthermore, to explore the models’ efficacy in the task of recurrence risk prediction, we also collect a recurrence cohort, comprising 5937 examinations from 1236 patients with prior BC history. Comprehensive demographic details of each respective set are provided in Supplementary Tables 3, 4.

#### Public dataset for further validation

For a comprehensive assessment of the proposed method, we incorporate the public CSAW-CC dataset^40^. The process of dataset acquisition is depicted in Supplementary Fig. 2. The full CSAW-CC dataset is leveraged for validation, which encompasses a total of 24,697 examinations derived from 8723 distinct women. As presented in Table 1, the dataset comprises 22,871 examinations within the cancer-free and 1826 examinations within the cases. About 45% of the cancer-free examinations and 60% of the case examinations are from women older than 55 years of age. Besides, the dataset offers labels for the cases across three distinct categories. Specifically, 524 examinations were diagnosed within 60 days, 267 examinations between 60 days and 2 years, and 1053 examinations were diagnosed with BC after 2 years during their follow-up.

### Risk prediction on the entire in-house test dataset

All concordance index (C-index) and area under receiver operating characteristics (AUC) results in the in-house test dataset are summarized in Table 2. As shown in Fig. 3 and Supplementary Table 15, the results of age-adjusted AUC (aAUC) are calculated following the method of a previous study^35^.

**Table 2:** Comparison of 10-year risk predictions on the in-house test set, three cohorts, and the CSAW-CC dataset

Method	C-Index	1-Year AUC	2-Year AUC	3-Year AUC	5-Year AUC	10-Year AUC
In-house test set: 6311 exams, 511 followed by cancer diagnosis within 1 year; 869 diagnosis within 5 years; 1132 diagnosis within 10 years.						
BI-RADS	0.69 (0.67–0.70)	0.83 (0.81–0.85)	0.78 (0.76–0.80)	0.74 (0.72–0.76)	0.70 (0.68–0.71)	0.65 (0.63–0.66)
STP-Baseline	0.64 (0.62–0.66)	0.74 (0.72–0.76)	0.70 (0.68–0.72)	0.67 (0.65–0.69)	0.62 (0.60–0.64)	0.53 (0.51–0.56)
Baseline-Risk factors	0.58 (0.56–0.59)	0.57 (0.55–0.59)	0.56 (0.54–0.58)	0.56 (0.54–0.58)	0.56 (0.54–0.58)	0.59 (0.56–0.61)
STP-Detection	0.69 (0.67–0.70)	0.74 (0.72–0.77)	0.72 (0.70–0.75)	0.72 (0.69–0.74)	0.70 (0.68–0.72)	0.69 (0.66–0.71)
STP-Transformer	0.73 (0.72–0.75)	0.84 (0.81–0.86)	0.80 (0.78–0.82)	0.78 (0.76–0.80)	0.74 (0.72–0.76)	0.71 (0.68–0.73)
MTP-BCR (Ours) Patient Level	0.82 (0.81–0.84)	**0.91 (0.89–0.92)**	**0.88 (0.86–0.89)**	**0.86 (0.84–0.87)**	**0.82 (0.81–0.84)**	**0.80 (0.78–0.82)**
MTP-BCR (Ours) Unilateral Breast Level	0.81 (0.79–0.82)	0.89 (0.87–0.91)	0.87 (0.85–0.88)	0.84 (0.83–0.86)	0.81 (0.79–0.82)	0.77 (0.75–0.78)
In-house Screening cohort 1 (Normal or biopsy–negative): 5,937 exams, 137 followed by cancer diagnosis within 1 year; 495 diagnosis within 5 years; 758 diagnosis within 10 years.						
BI-RADS	0.53 (0.52–0.55)	0.61 (0.57–0.65)	0.58 (0.55–0.60)	0.55 (0.54–0.58)	0.54 (0.52–0.55)	0.52 (0.51–0.53)
STP-Baseline	0.54 (0.52–0.56)	0.57 (0.52–0.62)	0.54 (0.51–0.58)	0.54 (0.50–0.57)	0.51 (0.48–0.54)	0.46 (0.44–0.49)
Baseline-Risk factors	0.57 (0.54–0.59)	0.52 (0.47–0.57)	0.52 (0.48–0.55)	0.53 (0.49–0.57)	0.54 (0.51–0.57)	0.58 (0.56–0.61)
STP-Detection	0.61 (0.59–0.63)	0.59 (0.54–0.65)	0.61 (0.57–0.65)	0.62 (0.59–0.65)	0.62 (0.60–0.65)	0.63 (0.60–0.66)
STP-Transformer	0.64 (0.62–0.66)	0.65 (0.60–0.70)	0.64 (0.60–0.67)	0.65 (0.62–0.68)	0.66 (0.63–0.68)	0.68 (0.66–0.71)
MTP-BCR (Ours) Patient Level	0.74 (0.72–0.76)	**0.77 (0.73–0.81)**	**0.75 (0.71–0.78)**	**0.75 (0.72–0.77)**	**0.73 (0.71–0.76)**	**0.73 (0.71–0.75)**
MTP-BCR (Ours) Unilateral Breast Level	0.72 (0.71–0.74)	0.76 (0.71–0.81)	0.75 (0.72–0.79)	0.74 (0.71–0.77)	0.72 (0.69–0.74)	0.69 (0.67–0.72)
In-house Screening cohort 2 (Normal BI-RADS): 5,139 exams, 102 followed by cancer diagnosis within 1 year; 404 diagnosis within 5 years; 612 diagnosis within 10 years.						
BI-RADS	0.50 (0.50–0.51)	0.53 (0.50–0.55)	0.51 (0.50–0.53)	0.51 (0.50–0.52)	0.51 (0.50–0.51)	0.50 (0.50–0.51)
STP-Baseline	0.54 (0.52–0.57)	0.56 (0.50–0.62)	0.54 (0.49–0.58)	0.53 (0.49–0.57)	0.50 (0.47–0.54)	0.46 (0.43–0.49)
Baseline-Risk factors	0.56 (0.54–0.59)	0.52 (0.45–0.58)	0.51 (0.46–0.56)	0.53 (0.48–0.57)	0.55 (0.51–0.58)	0.58 (0.55–0.61)
STP-Detection	0.61 (0.59–0.64)	0.61 (0.54–0.67)	0.60 (0.56–0.65)	0.62 (0.58–0.66)	0.62 (0.59–0.65)	0.63 (0.60–0.66)
STP-Transformer	0.62 (0.60–0.65)	0.63 (0.57–0.68)	0.61 (0.57–0.65)	0.63 (0.59–0.66)	0.64 (0.61–0.67)	0.67 (0.65–0.70)
MTP-BCR (Ours) Patient Level	0.73 (0.71–0.75)	**0.74 (0.69–0.79)**	**0.73 (0.69–0.76)**	**0.73 (0.70–0.76)**	**0.72 (0.70–0.75)**	**0.73 (0.71–0.76)**
MTP-BCR (Ours) Unilateral Breast Level	0.73 (0.71–0.75)	0.77 (0.72–0.81)	0.75 (0.72–0.79)	0.74 (0.71–0.77)	0.72 (0.69–0.74)	0.70 (0.67–0.72)
In-house recurrence cohort: 3232 exams, 121 followed by cancer diagnosis within 1 year; 357 diagnosis within 5 years; 511 diagnosis within 10 years.						
BI-RADS	0.58 (0.56–0.60)	0.80 (0.74–0.86)	0.68 (0.64–0.73)	0.62 (0.59–0.66)	0.59 (0.56–0.61)	0.55 (0.52–0.57)
Baseline-Risk factors	0.58 (0.55–0.66)	0.59 (0.52–0.65)	0.55 (0.50–0.61)	0.56 (0.52–0.61)	0.56 (0.52–0.60)	0.58 (0.54–0.62)
Baseline-Recurrence	0.62 (0.59–0.65)	0.64 (0.57–0.71)	0.63 (0.58–0.68)	0.62 (0.57–0.66)	0.61 (0.57–0.64)	0.59 (0.54–0.63)
STP-Detection	0.63 (0.60–0.66)	0.73 (0.67–0.80)	0.69 (0.64–0.74)	0.66 (0.61–0.70)	0.63 (0.60–0.67)	0.62 (0.58–0.66)
MTP-BCR (Ours) Patient Level	0.72 (0.69–0.74)	0.82 (0.76–0.87)	**0.79 (0.75–0.83)**	**0.76 (0.72–0.79)**	**0.71 (0.68–0.75)**	0.64 (0.59–0.68)
MTP-BCR (Ours) Unilateral Breast Level	0.69 (0.66–0.71)	0.78 (0.72–0.83)	0.76 (0.71–0.80)	0.72 (0.68–0.76)	0.68 (0.65–0.71)	0.61 (0.57–0.64)
Method	C-Index	1-Year AUC	2-Year AUC	7-Year AUC		
CSAW-CC dataset: 24,697 exams, 1826 followed by cancer diagnosis.						
STP-Baseline	0.58 (0.57–0.59)	0.68 (0.65–0.71)	0.63 (0.61–0.65)	0.58 (0.57–0.59)		
STP-Detection	0.67 (0.66–0.68)	0.83 (0.81–0.85)	0.76 (0.75–0.78)	0.67 (0.66–0.68)		
STP-Transformer	0.69 (0.68–0.70)	0.86 (0.85–0.88)	0.78 (0.77–0.80)	0.69 (0.68–0.70)		
MTP-BCR (Ours) Patient Level	0.74 (0.73–0.75)	0.88 (0.86–0.90)	**0.81 (0.79–0.83)**	**0.74 (0.72–0.75)**		
MTP-BCR (Ours) Unilateral Breast Level	0.74 (0.72–0.75)	0.90 (0.88–0.91)	0.82 (0.81–0.84)	0.74 (0.72–0.75)		

C-index and AUC results are presented with a 95% Confidence Interval. Bold: *P* < 0.05, the AUCs of our methods are significantly higher than all other models for the same time horizon. Baseline-risk factors: SVM model based on risk factors; Baseline-recurrence: a traditional machine learning (SVM)-based recurrence risk prediction model, leveraging risk factors and prognostic factors. STP-Baseline: Single-time point (STP) image-only based baseline DL methods; STP-Detection: STP-based DL detection method; STP-transformer: STP-based DL risk prediction method, which leverages the transformer to fuse the representation of each view.

**Fig. 2:**
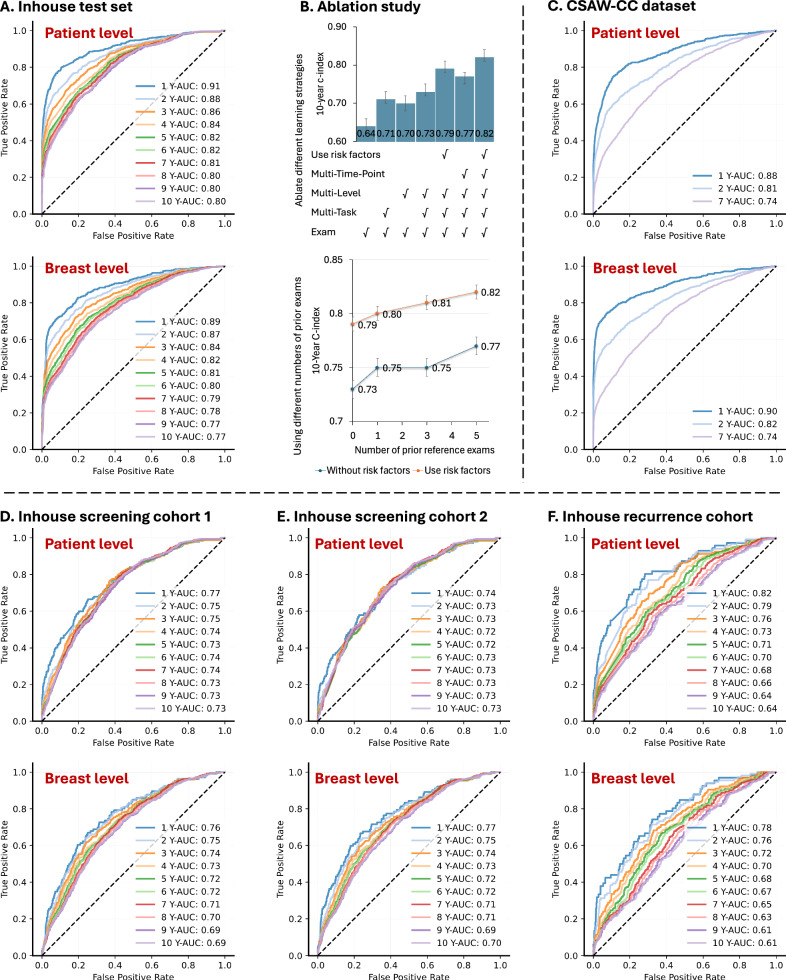
ROC curves and ablation experiments results of the risk prediction of the MTP-BCR model. **A** ROCs for the MTP-BCR method patient- and breast-level prediction on the entire in-house test set. **B** Ablation experiments results on the entire in-house test set. The first plot shows that our proposed learning strategies can improve the ability of the risk model. The second plot shows that the performance of our risk model improves with more of the prior reference exams. **C** ROCs for MTP-BCR method patient- and breast-level prediction on the CSAW-CC dataset. **D**–**F** ROCs for risk prediction on three in-house cohorts.

Our method’s performance: MTP-BCR method achieved a 10-year C-index of 0.82 (95% CI, 0.81–0.84) for patient-level risk prediction (Table 2 and Fig. 2A), with AUCs of 0.91 (95% CI, 0.89–0.92) for 1-year risk and 0.80 (95% CI, 0.78–0.82) for 10-year risk. At the breast-level, similar performance was observed, with a 10-year C-index of 0.81 (95% CI, 0.79–0.82). To account for age-related variability^41^, we calculated aAUC for 1- to 10-year risk, ranging from 0.91 to 0.77 (patient-level) and 0.89–0.74 (breast-level; Supplementary Table 15). These results demonstrate the accuracy of MTP-BCR in predicting unilateral breast cancer risk across different time horizons.

**Fig. 3:**
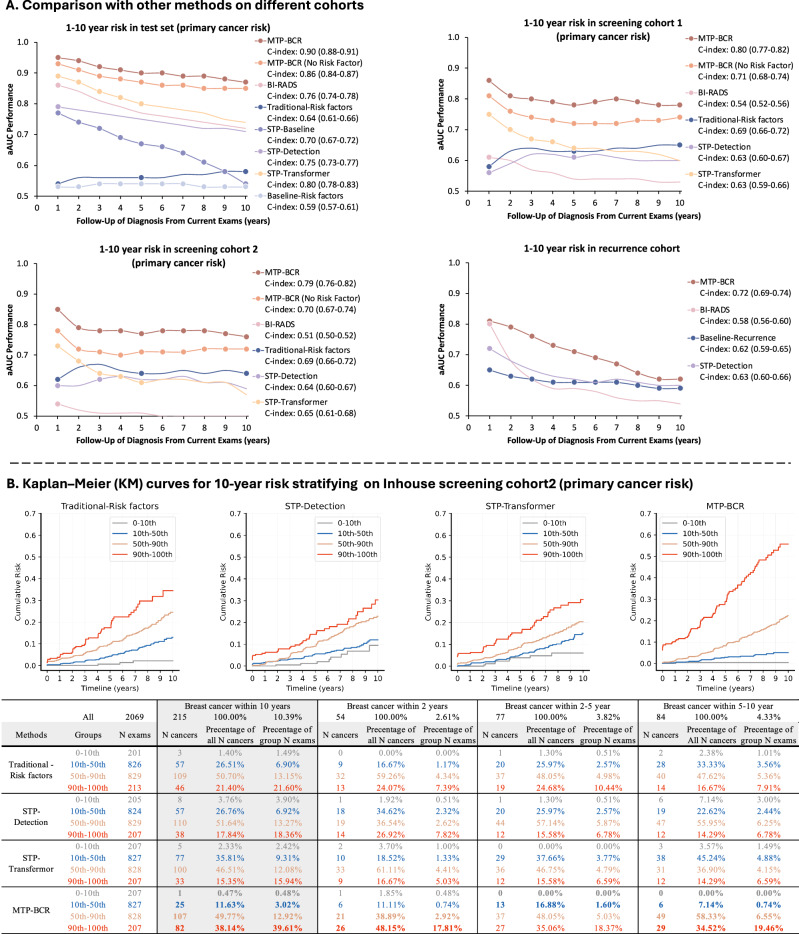
Comparison results of risk prediction. **A** The comparison results of age-adjusted AUC (aAUC) based on the different cohorts. Traditional risk model (BCSC) based on risk factors; Baseline-risk factors: SVM model based on risk factors; Baseline-recurrence SVM: a traditional machine learning (SVM)-based recurrence risk prediction model, leveraging risk factors and prognostic factors. STP-baseline: Single-time point (STP) image-only based baseline DL methods; STP-detection: STP-based DL detection method; STP-transformer: STP-based DL risk prediction method, which leverages the transformer to fuse the representation of each view. **B** Kaplan–Meier (KM) curves for 10-year risk stratifying by different methods on primary screening cohort 2. Groups are divided by the predicted risk scores of each model, including (1) 0 to 10th percentile, (2) 10th to 50th percentile, (3) 50th to 90th percentile, and (4) 90th and up. The summary table for each percentile range was provided, detailing the number of women, the number of cancers, the percent of cancers accounted for women of each group, and the percent of group cancers accounted for all cancers.

Comparing with other methods: as shown in Table 2, the 10-year C-index of the lowest performance baseline image-only method (STP-baseline) is 0.64 (95% CI, 0.62–0.66). For the 1-year AUC of radiologists’ BI-RADS assessments and the STP-Detection method are 0.83 (95% CI, 0.81–0.85) and 0.74 (95% CI, 0.72–0.77), respectively, which are significantly lower than the MTP-BCR method (1-year AUC: *P* < 0.001, DeLong’s test). Furthermore, for the BC risk prediction, the AUC results show that the MTP-BCR method significantly outperforms all other methods at all time points (2- to 10-year AUC: *P* values <0.05, DeLong’s test). We also observe the consistent advantage of our methods in the aAUC results (Supplementary Table 15). For a fair comparison with the traditional-risk factors (BCSC) model, we exclude the women ineligible for risk calculation by the model. The AUC results (Supplementary Table 8) show that MTP-BCR models significantly outperform the 1-year, 5-year, and 10-year BCSC risk models (1-, 5-, and 10-year AUC: *P* values <0.001, DeLong’s test). The latter obtains AUCs of 0.62 (95% CI, 0.58–0.64), 0.65 (95% CI, 0.62–0.68), and 0.71 (95% CI, 0.68–0.74), respectively. Additionally, MTP-BCR models outperform all other methods for 1- to 10-year risk predictions in this population (all *P* values <0.001, DeLong’s test). The curves of aAUC values for cumulative risk at multiple time points of all methods are shown in Fig. 3A. We note that after adjusting by age, the corresponding aAUCs are 0.54, 0.56, and 0.58 for the traditional-risk factors (Supplementary Table 15).

Ablation experiments: to identify the optimal design for the MTP-BCR model, we conducted a series of ablation studies (Fig. 2B and Supplementary Tables 5–7). The results, measured by C-indices (Fig. 2B) and AUCs (Supplementary Table 5), demonstrate that our proposed learning strategies: multi-task, multi-level, and multi-time-point, outperform individual strategies when used alone. Specifically, compared to the baseline method, which has a 10-year C-index of 0.64, the incorporation of “multi-task”, “multi-level”, “multi-task + multi-level” and “multi-task + multi-level + multi-time-point” learning strategies enhances the C-index by 7, 6, 9, and 15%, respectively. Notably, even without the inclusion of traditional risk factors, our purely image-based MTP-BCR method achieved a C-index of 0.77, surpassing all single-time-point (STP)-based methods, including the top-performing STP-Transformer, which achieved a C-index of 0.73. This underscores the superiority of our MTP-based deep learning approach. Additionally, the inclusion of risk factors further improves the model’s performance, demonstrating the value of integrating both image-based and traditional risk data.

We also explore the impact of using different numbers of prior reference exams during training. As shown in Fig. 2B and Supplementary Table 6, the model performs best when trained with five historical mammogram references. Furthermore, Supplementary Table 7 reveals that training the model on the full dataset results in the highest C-index across the entire test set and all sub-cohorts, outperforming models trained solely on primary or recurrence datasets. These findings indicate that training on more diverse information provided by the full dataset enhances the model’s ability to capture a broader range of risk factors, thereby leading to superior predictive performance for both primary and recurrence risk prediction. Besides, we also conducted an additional patient-based evaluation, where only one exam per patient was randomly selected. This ensures statistical independence across observations. The results show our model’s performance remained stable and consistently outperformed other methods (Supplementary Table 19).

### Risk prediction on the external CSAW-CC dataset

All C-index and AUC results on the CSAW-CC dataset are summarized in Table 2. On this pure-image-based dataset, our MTP-BCR method obtains a C-index of 0.74 (95% CI, 0.73–0.75) compared with C-indices of STP-baseline, STP-detection, and STP-transformer methods of 0.58 (95% CI, 0.57–0.59), 0.67 (95% CI, 0.66–0.68), and 0.69 (95% CI, 0.68–0.70), respectively. For unilateral breast-level prediction, MTP-BCR obtains a C-index of 0.74 (95% CI, 0.72–0.75) (Fig. 2C). The 2- and 7-year AUCs in the BC risk prediction by our MTP-BCR are 0.81 and 0.74, significantly higher (all *P* values <0.05, DeLong’s test) than the best performance competitive model, STP-Transformer (which achieves 0.78 and 0.74, for the different time points). The corresponding AUCs for breast-level cancer prediction by MTP-BCR are 0.82 and 0.74, respectively. Supplementary Table 10 further shows the risk stratification performance of different models, and Supplementary Table 11 provides a comprehensive comparison with the MIRAI model^21^. These findings support our conclusion that modeling breast tissue changes over time enhances the prediction of future breast cancer risk.

### Risk prediction performance of MTP-BCR on screening populations

In screening cohort 1, our MTP-BCR method demonstrates superior performance in C-index and AUC metrics (ROCs of each time point are provided in Fig. 2D). Short-term (1 year) risk prediction can be equivalent to interval cancer detection. As shown in Table 2, MTP-BCR achieves 0.77 (95% CI, 0.73–0.81) when using risk factors, significantly higher than that from BI-RADS scores with 0.61 (95% CI, 0.57–0.65), the STP-detection method with 0.59 (95% CI, 0.54–0.65), and also the STP-transformer with 0.65 (95% CI, 0.60–0.70). For 10-year risk, our MTP-BCR method reaches the highest C-index of 0.74 (95% CI, 0.72–0.76), versus 0.64 (95% CI, 0.62–0.66) for the STP-Transformer (Table 2). Our models continue to outperform other methods after adjusting for age, as shown in Supplementary Table 13 and Fig. 3A of aAUC results.

In Supplementary Table 9, the Traditional-Risk factors (BCSC) 1-year risk model obtains an AUC of 0.70, which is significantly lower than the AUCs of our MTP-BCR model, which reaches 0.87. For 5-year risk, MTP-BCR achieves a C-index of 0.79 (95% CI, 0.76–0.82) compared to 0.69 (95% CI, 0.65–0.72) for the BCSC model (Supplementary Table 9). MTP-BCR method significantly outperforms all other models by AUCs at each time point for 10-year risk (all *P* values <0.05, DeLong’s test). After adjusting for age, MTP-BCR achieves a 10-year aAUC of 0.78 with risk factors, outperforming the BCSC model’s 0.65 by 0.13 (Supplementary Table 17). The AUC and C–index metrics show that our MTP-BCR method performs similarly for unilateral breast-level BC risk prediction and patient-level cancer risk prediction.

In screening cohort 2: in this set, we evaluate risk models when radiologists cannot give any suspicious findings on mammograms. As expected, the C-indices and AUCs of the BI-RADS are close to 0.5, as shown in Table 2 and Supplementary Table 9. Corresponding results of aAUCs are provided in Supplementary Tables 16, 17 and Fig. 3A. In contrast, our MTP-BCR (ROCs are provided in Fig. 2E) is still significantly better than all other methods among the short-term, with a 1-year AUC of 0.74, long-term, with a 5-year AUC of 0.72, and longer-term with a 10-year AUC of 0.73 risk predictions. Especially in the traditional-risk factors model target population (aged 35–74, without prior BC history), the 1-year aAUC of our MTP-BCR risk-based model reaches 0.85 while the radiologists and the cancer detection-based models fail to outperform random guessing (as shown in Fig. 3A).

We also compare the ability of risk stratification of different models by dividing the population into specified percentile ranges (0–10th, 10–50th, 50–90th, 90th, and up). The Kaplan–Meier (KM) curves (Fig. 3B) show that the MTP-BCR method better stratifies the high- (higher than 90th) and low- (lower than 10th) risk women. Women with MTP-BCR risk above the 90th percentile accounted for 38% of all cancers by 10 years, while those in the 10th percentile accounted for only 0.47% of all cancers. For this cohort, representing women who may benefit from supplemental imaging, we also evaluated the performance of 2-year risk stratification, which could be related to interval cancer detection. MTP-BCR achieved the highest 2-year interval cancer detection rate at 48.15%. Additionally, for both 2–5 or 5–10 year risk prediction, MTP-BCR consistently outperformed other methods, demonstrating superior stratification of high- and low-risk populations.

### Risk prediction performance of MTP-BCR on the recurrence cohort

In this set, we evaluate the models’ ability to predict recurrence risk, defined as subsequent cancer events in patients with a prior BC history, including ipsilateral (same breast) and contralateral (opposite breast) cancers. As shown in Table 2, our MTP-BCR method significantly outperforms the traditional recurrence risk model (Baseline-Recurrence) across all time points (1- to 10-year AUC: 0.82–0.64 vs. 0.64–0.59; *P* values <0.05, DeLong’s test). The traditional recurrence risk model (Baseline-Recurrence model; risk factor and prognostic factor-based) is better than only the risk-factor-based model. While the BI-RADS method achieves a 1-year AUC of 0.80 (1-year AUC *P* values = 0.624, DeLong’s test), our MTP-BCR model performs significantly better for 2- to 10-year predictions (2- to 10-year AUC *P* values <0.001, DeLong’s test). Moreover, the STP-Detection method obtains performances ranging between 0.73 and 0.62 for 1- to 7-year AUC, which are significantly lower than achieved with the MTP-BCR method (1- to 7-year AUC *P* values <0.05, DeLong’s test). Age-adjusted results confirm MTP-BCR as the best-performing model across all time points (Fig. 3A, Supplementary Table 18).

We also report separate results for ipsilateral and contralateral recurrence (Supplementary Table 12). For the contralateral recurrence risk prediction, the MTP-BCR method with and without risk factors at the patient level risk prediction obtained a 10-year C-index of 0.79 and 0.72. Adding risk and prognostic factors improves AUCs by 0.04–0.05. For the ipsilateral recurrence risk prediction, the 10-year C-indices of MTP-BCR models (with/without risk factor) are 0.69 and 0.56. Correspondingly, the AUCs results demonstrated larger gaps between with/without risk factor MTP-BCR models compared with those of the contralateral recurrence group. Similar phenomena are more obvious at the breast level, indicating cancer characteristics affect the ipsilateral recurrence risk the most.

### The ability of short and long future BC risk assessment

While the results highlight the advantages of our method in risk prediction, assessing true long-term BC risk after removing biases from cancer detection and short-term risk predictions requires further analysis. To address this, we evaluate 5- and 10-year risk prediction on in-house test sets, excluding exams from women diagnosed within 1, 3, and 5 years (Table 3). In these extremely challenging scenarios, the MTP-BCR method achieves AUCs of 0.72 and 0.73 for 5-year and 10-year risk prediction. These results were significantly higher than other methods (all *P* values <0.001, DeLong’s test). These results show that our methods could not only detect BC and improve the performance of short-term risk prediction compared with other methods, but also learn the features related to the real longer-term (10 years) risk. A more granular analysis of AUC results over time is provided in Supplementary Fig. 4.

**Table 3:** Comparison of long-term risk predictions

Method	2–5 Year AUC	4–5 Year AUC	2–10 Year AUC	4–10 Year AUC	6-10 Year AUC
Full test set					
BI-RADS	0.51 (0.50–0.52)	0.50 (0.49–0.52)	0.50 (0.49–0.51)	0.50 (0.49–0.51)	0.50 (0.49–0.52)
Baseline-Risk factors	0.56 (0.53–0.60)	0.59 (0.54–0.64)	0.60 (0.57–0.63)	0.63 (0.60–0.67)	0.66 (0.62–0.70)
STP-Detection	0.63 (0.60–0.66)	0.61 (0.57–0.65)	0.63 (0.60–0.65)	0.61 (0.58–0.64)	0.60 (0.57–0.64)
STP-Transformer	0.60 (0.57–0.63)	0.58 (0.53–0.63)	0.60 (0.57–0.62)	0.59 (0.56–0.62)	0.60 (0.56-0.63)
MTP-BCR (Ours) Patient Level	**0.72 (0.69–0.74)**	**0.72 (0.68–0.75)**	**0.73 (0.70–0.75)**	**0.73 (0.70–0.75)**	**0.73 (0.70–0.76)**
MTP-BCR (Ours) Unilateral Breast Level	0.71 (0.68–0.73)	0.69 (0.66–0.73)	0.69 (0.66–0.71)	0.68 (0.66–0.71)	0.69 (0.66–0.72)
Primary screening population (35–74 years old without prior BC)					
BI-RADS	0.52 (0.49–0.55)	0.50 (0.47–0.53)	0.52 (0.50–0.54)	0.51 (0.49–0.53)	0.51 (0.49–0.54)
Traditional- Risk factors	0.69 (0.64–0.74)	0.67 (0.60–0.74)	0.73 (0.69–0.77)	0.72 (0.67–0.76)	0.71 (0.66–0.76)
Baseline-Risk factors	0.67 (0.61–0.72)	0.66 (0.58–0.74)	0.71 (0.67–0.75)	0.71 (0.66–0.76)	0.72 (0.66–0.78)
STP-Detection	0.66 (0.61–0.71)	0.64 (0.58–0.71)	0.63 (0.59–0.67)	0.62 (0.57–0.67)	0.61 (0.55–0.67)
STP-Transformer	0.59 (0.54–0.65)	0.57 (0.49–0.64)	0.57 (0.53–0.62)	0.56 (0.51–0.61)	0.56 (0.50–0.63)
MTP-BCR (Ours) Patient Level	**0.76 (0.72–0.80)**	**0.76 (0.71–0.81)**	**0.79 (0.75–0.82)**	**0.79 (0.75–0.82)**	**0.79 (0.75–0.83)**
MTP-BCR (Ours) Unilateral Breast Level	0.74 (0.70–0.78)	0.73 (0.68–0.78)	0.72 (0.68–0.75)	0.72 (0.68–0.75)	0.73 (0.68–0.77)
Method	2–7 year AUC	3–7 year AUC			
CSAW: only women who completed scored across the BCSC model					
STP-Detection	0.61 (0.59–0.62)	0.60 (0.58–0.62)			
STP-Transformer	0.62 (0.60–0.63)	0.61 (0.60–0.63)			
MTP-BCR (Ours) Patient Level	**0.68 (0.66–0.69)**	**0.68 (0.66–0.69)**			
MTP-BCR (Ours) Unilateral Breast Level	0.67 (0.66–0.69)	0.67 (0.65–0.68)			

AUC results are presented with a 95% Confidence Interval. Bold: *P* < 0.05, the AUCs of our methods are significantly higher than all other models for the same time horizon. Traditional-risk factors: Traditional risk model (BCSC) based on risk factors; Baseline-risk factors: SVM model based on risk factors; STP-baseline: Single-time point (STP) image-only based baseline DL methods; STP-detection: STP-based DL detection method; STP-transformer: STP-based DL risk prediction method, which leverages the transformer to fuse the representation of each view. Note that the results of the traditional risk model are based on part of the full in-house test set, as they are out of the age range of 35–74 or with prior BC history. For fair comparison, we also implemented the comparison experiments excluding the women who were not eligible according to the traditional risk model.

Furthermore, we also conduct this experiment on the external CSAW-CC dataset. Specifically, when excluding the exams from women diagnosed with cancer within 1 year or 2 years, our MRP-BCR model obtains an AUC of 0.68 (95% CI, 0.66–0.69) and 0.68 (95% CI, 0.66–0.69) for risk prediction.

### Sub-group analysis

**Fig. 4:**
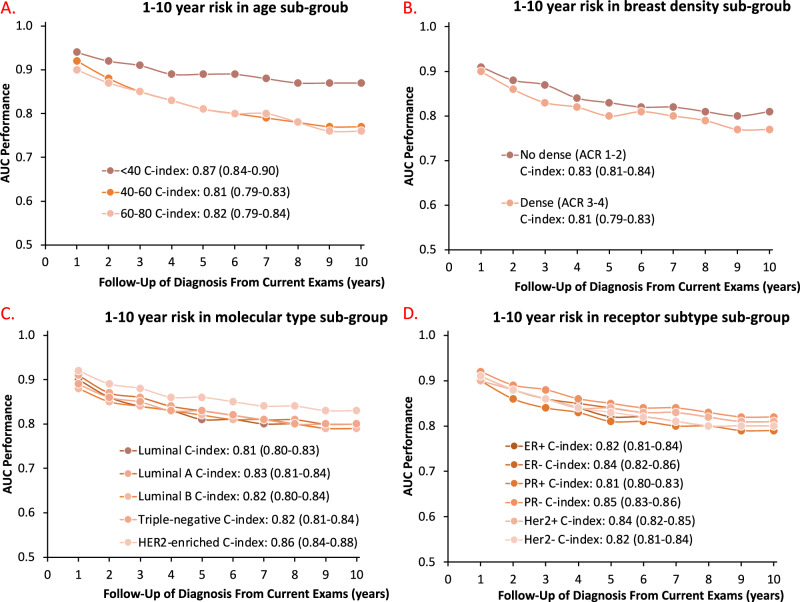
Cumulative risk at multiple time points on different sub-groups. **A–D** AUC performance of the MTP-BCR model across different subgroups, based on (**A**) age, (**B**) breast density, (**C**) molecular subtypes, and (**D**) receptor subtypes of future tumors.

To distinguish how our MTP-BCR method performs in different populations and to determine the potential population that can benefit the most from it, we evaluate our risk model in different clinical subgroups, based on age, breast density, molecular subtypes, and receptor subtypes of future tumors on in-house dataset (Fig. 4). We find that MTP-BCR method performed similarly across different density groups and independently from future cancer sub-types. We also compare the C-index of 10-year risk prediction of different methods in different subgroups, which are available in Supplementary Table 13. We also created the subsets by collecting all mammograms based on the same scanner type to assess the impact of different mammography equipment on the performance of our model. An obvious bias-related difference in the different types of scanners was not found. Due to a lack of race labels, the group analysis for different race groups is not performed. Moreover, the subgroup analysis on CSAW-CC is shown in Supplementary Table 14. The results consistently demonstrate the advantages of the MTP-BCR method, with performance either superior or comparable across these subgroups.

### Consistency of model attention in longitudinal images

**Fig. 5:**
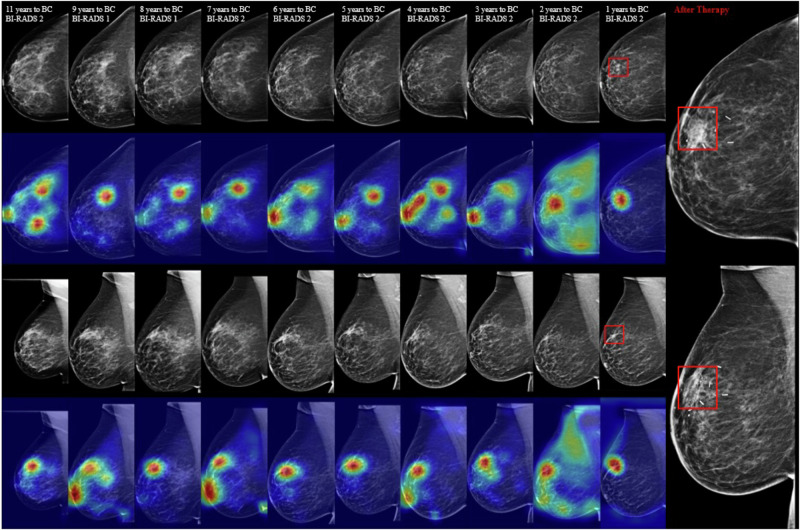
An example of a class activation map (CAM) visualization. The longitudinal craniocaudal (CC) and mediolateral oblique (MLO) mammograms were acquired from a patient who participated in ten consecutive BC screening from 2005 to 2015, culminating in a BC diagnosis during the last screening (invasive ductal and lobular carcinoma located at C50.4, exhibiting positive expression of estrogen receptor (ER+), progesterone receptor (PR+), and human epidermal growth factor receptor 2 (Her2Neu+)). The closer to red, the more relevant the pixel is to the risk prediction.

To investigate how risk-related areas evolve across multiple time mammograms that our MTP-BCR method focused on, the gradient-weighted class activation maps (Grad-CAM)^42^ method is employed. Figure 5 presents a visualization example of a BC patient. The heatmaps highlight potentially related regions where our proposed MTP-BCR method identifies predictive imaging features for BC risk. While this visualization is a preliminary process, results show that the high-risk regions from multiple time point examinations that our model focuses on are relatively consistent. Moreover, the heatmaps show that our risk model could accurately figure out high-risk areas of short-term BC at two views of mammograms. Besides, the previous research^43^ demonstrated that the cancer signs model exhibits sharp activations localized to the tumor, whereas the inherent risk model has broad activations and is more diffusely spread through the parenchyma. Our MTP-BCR risk model not only relies on a broad range of breast cues but also concentrates on localized areas that are near tumor-like patterns, demonstrating that our model combines BC detection and future cancer risk prediction tasks.

## Discussion

In this study, we develop a multi-time-point examination learning-based risk model, MTP-BCR, to assess BC risk at both the patient and unilateral breast level using longitudinal screening mammograms and medical records. From short-term risk to long-term risk prediction, MTP-BCR outperforms radiologists’ BI-RADS assessment, single-time-point-based DL approaches (STP-Detection, STP-Transformer), and a traditional-risk factors model (BCSC). In addition to the risk at the patient level, our method could also estimate the risk at the breast level with comparable ability. Experiments on CSAW-CC and different cohorts of the in-house dataset suggest that the longitudinal assessment of MTP-BCR can accurately identify short-/longer-term, risk-related features on mammograms, which is further supported by consistent heatmaps of multi-time point mammograms. Our model also outperforms traditional recurrence risk models in the related task^39^. Finally, subgroup analysis indicates that the proposed method performs consistently across subgroups of different breast densities and for future types of BC.

The motivation to develop BC risk models is to guide short- to long-term personalized screening or prevention regimens. The idea is to determine the screening frequency and the appropriate screening modality based on the individual risk of women, potentially also to recommend supplemental MRI^44^ or preventive therapy for women at a high risk of developing BC^17^. Based on risk factors such as age, genetic determinants, family history, and recently considered image-based breast density, traditional risk models are used to globally assess 5-year, 10-year, or lifetime risk for large groups of women^45^. However, our results show that density alone is not sufficient to represent all of the risk-related information from mammograms. Moreover, ignoring the short-term risk of BC limits the value of these models in early BC detection. Recent DL-based risk models may fully utilize screening images but mainly target short-term risk prediction or interval cancer detection while ignoring long-term risk, which limits the ability to offer personalized screening recommendations and preventive interventions.

Specifically, the short-term risk models could identify women who, after a negative or benign screening, might benefit from supplemental or more intensive screening and identify interval cancer^41^. The long-term assessment model could advocate less frequent screening for the low-risk population to reduce the harm and cost of screening^13,14^. Long-term risk assessment is reasonable considering that the tumor development time for screening or clinically detected cancer is estimated to be 5–20 years^41,46^. It should be noted that the screening strategy can be adjusted dynamically as our model can accept late screening information into the historical inputs and update the risk prediction. More importantly, recent research demonstrates that prophylactic interventions (including lifestyle changes and risk-reducing medications) have shown promising results in reducing BC incidence in women with an increased risk of BC^47^. For women with a high 10-year or lifetime risk of BC, the NICE and USPSTF clinical guidelines recommend risk-reducing interventions or more intense screening^41,48,49^. In contrast, our MTP-BCR method combines the advantages of both short- and long-term risk prediction strategies. A longer time-horizon enables our model to capture and adapt to changes in a woman’s risk profile over an extended period. Thus, it allows for a more comprehensive understanding of a woman’s evolving risk and enhances the precision of future risk prediction.

MTP-BCR method demonstrates a superior capability in short-term risk prediction. Our analysis of the full test set reveals that, in realistic and complex clinical screening scenarios, the MTP-BCR method consistently outperforms other methods, functioning independently of radiological interpretation. Traditional risk assessment tools relying on relatively static patient demographics and clinical information are limited in their ability to estimate short-term future cancer risk accurately and typically ignore the detection of existing cancer^14^. The “1-year” outcome in the CSAW-CC dataset reflects current cancer detection (Table 2), showing that the cancer detection task mainly relies on searching tumor evidence with smaller benefits added by dynamic visual features. However, such STP methods are limited by static visual features in predicting future risk^22^. In contrast, the MTP-BCR method enhances risk prediction accuracy by incorporating dynamic risk features through the comparison of current and prior exams.

For instance, in a screening cohort characterized by normal BI-RADS findings (cohort 2), our model achieved the highest AUC of 0.74 (95% CI, 0.69–0.79) for 1-year risk prediction. This suggests that the MTP-BCR method could serve as a valuable aid for radiologists, potentially improving the detection of interval cancers across the entire screening population, including both primary and recurring cancers. For interval cancer risk prediction on the primary screening population, our MTP-BCR method achieved an AUC of 0.87 (95% CI, 0.81–0.92). Notably, for women at high risk (90th percentile and above), the model detected ~20% more cancers than other existing methods (Fig. 3). To further support radiologists in clinical decision-making, our method is also capable of estimating unilateral breast-level risk with performance comparable to that of patient-level risk prediction. This dual-level risk estimation, along with the model’s retrospective analysis and visualizations, underscores the potential of the MTP-BCR method to enhance early detection and reduce the incidence of interval cancers by guiding radiologists in identifying high-risk regions on mammographic images.

MTP-BCR method consistently outperforms other methods in long-term risk stratification across screening populations. This enhanced performance is consistent across various demographic subgroups. Notably, the model maintains robust accuracy in predicting long-term risk at both the patient and breast levels, even when short-term cancers are excluded. Validation against the external CSAW-CC dataset, which relies solely on mammograms, further underscores the effectiveness of our MTP-based approach in capturing true future breast cancer risk based on multiple mammograms (Table 3). These results suggest that the MTP-BCR method effectively captures the true risk of future BC based on multiple mammograms and risk factors.

Specifically, KM curve analyses show that the MTP-BCR method identifies ~17% more high-risk women (in the 90th percentile and above) likely to be diagnosed with breast cancer within 10 years compared to other methods, enabling more aggressive screening and prevention strategies^44^. On the other hand, for the lower-risk population (in the 10th percentile and below), the MTP-BCR method reduces the false negative rate by 67–88% for patients who are misclassified as low-risk but will develop breast cancer within 10 years. This enhanced accuracy in identifying truly healthy women could potentially reduce the frequency of unnecessary BC screenings^14^. The improved capability of risk stratification enhances the potential utility of the risk model in optimizing risk-adapted screening regimens and preventive therapies.

MTP-BCR method demonstrates a significant improvement in predicting recurrence risk in BC patients. Notably, our study includes patients with a history of BC, a worth noting group often excluded from previous risk models. This inclusion is critical, as it enhances the model’s applicability and relevance in real-world scenarios. Current survival rates for BC patients are substantial, with over 87% surviving five years and more than 77% surviving 10 years^50^. Thus, models that include recurrence risk also have huge potential for reducing unnecessary follow-up, reducing the possibility of inflicting psychological harm among low-risk women^51^. On the other hand, for high-recurrence risk patients, recommendations for more extensive or different examination or better preventive therapy may reduce the mortality rate^39^.

Existing recurrence risk models often focus primarily on tumor-related prognostic factors, neglecting individualized mammographic characteristics. However, our study demonstrates that mammograms provide significant predictive value and offer a more comprehensive view of a patient’s prognosis. MTP-BCR method innovatively integrates both prior tumor prognostic information^29^ and mammographic data, enhancing the accuracy of recurrence risk prediction. It outperforms traditional recurrence risk models and other image-based DL methods, suggesting that the MTP-BCR method could serve as a robust foundation for developing more tailored screening and prevention strategies at the individual breast level.

The promising performance of MTP-BCR can be attributed to its capacity to capture BC risk-related characteristics. Specifically, unlike conventional methods that often rely on static data, our proposed method incorporates both static features from single time-point exams and dynamic features from multiple time-point exams to capture the development risk of BC. Recently, a study^22^ also explored the potential of using longitudinal mammogram examinations to improve short-term risk prediction. However, we note that it has been restricted to a small image-only dataset and a setting of a fixed number of input time points, which hinders its clinical application. In contrast, our MTP-BCR method is developed using a large clinical screening mammogram dataset, allowing for the efficient integration of risk factors and offering the flexibility to accommodate 0–5 historical reference exams, thereby enhancing its clinical utility. In real-world clinical applications, the ability to use longitudinal data more effectively allows clinicians to track changes over time, improving the accuracy of both short- and long-term risk predictions. This dynamic assessment capability is particularly valuable in tailoring personalized screening and prevention strategies, potentially leading to earlier detection and better outcomes.

Additionally, the multi-task learning strategy enhances the extraction of relevant features from mammographic images and also improves the generalization of the DL model^52^. Our proposed multi-level learning strategy enables our model to learn the relationship between local (unilateral breast) level risk and global (patient) level risk while keeping the local information as much as possible when combining the multiple local features and summarizing them for a comprehensive risk assessment. As a result, the MTP-BCR method can consistently focus on corresponding regions in longitudinal mammograms without the need for registration. Moreover, our model demonstrates comparable performance at both the patient and breast levels of risk assessment. This robust performance across different patient and breast levels ensures that it can be reliably applied in diverse clinical settings, enhancing its utility in routine BC screening programs.

This research has limitations. Mammograms are generally large in size, but due to limitations in GPU resources, it is often necessary to downsample the images to a smaller size for general DL models. Mammogram image sizes used in previous mammography-based risk prediction studies varied from 224 × 224 to 2048 × 1664^14,19,21,22,24,43,53,54^. Considering the trade-off of the GPU memory and the number of historical references, the input mammograms are resized to a size of 1024 × 512. In the future, the optimized image size related to BC risk prediction tasks should be further explored. While we incorporated prior tumor information and therapy records to improve our risk model’s performance on BC recurrence risk, gaps exist between the subgroups with and without prior BC. More efforts are needed to improve the performance of recurrence risk prediction. Further validation of our model is required before it can be broadly implemented in clinical practice. For instance, more detailed demographics (e.g., race) are required to prove its generalizability. Besides, further investigations are required to assess the practical utility of heatmaps in assisting radiologists during image interpretation. For example, conducting a reader study to evaluate the incorporation of the risk model into the radiologist workflow could be a future direction for shedding light on the potential benefits of risk models for personalized BC screening policies.

In conclusion, we propose a novel DL model using longitudinal mammogram examinations and history obtained from medical records that outperform the single time point-based methods and traditional risk factor-based methods. The improvement based on analyzing historical tissue changes on mammograms is consistent across screening and future risk subgroups and is further proven by further validation. These results support the hypothesis that longitudinal mammography contains informative spatiotemporal indicators of future breast risk that cannot be captured by the single-time point DL models. Multi-time point models based on longitudinal analysis strategies have the potential to replace single-time point-based risk prediction models. MTP-BCR method also outperforms other recurrence risk methods in the recurrence risk prediction task. Apart from increasing the accuracy of BC risk prediction, we also improve the interpretability of our risk model, which could potentially accelerate the translation of personalized AI-based risk stratification into routine BC screening policies.

## Methods

We reported our study in line with the TRIPOD-AI (transparent reporting of a multivariable prediction model for individual prognosis or diagnosis) recommendations (Supplementary material)^55^.

### In-house dataset

Our retrospective study was conducted in accordance with the Declaration of Helsinki and was approved by the Institutional Review Board (IRB) of the Netherlands Cancer Institute (protocol number: IRBd21-060, approved in 2021). The requirement for informed consent was waived by the IRB due to the retrospective nature of the study and the use of anonymized data. A flowchart illustrating the construction of this large study dataset is shown in Supplementary Fig. 2. We collected 29,946 patients recorded in our hospital between January 1, 2004, and December 31, 2020. Then we collect the consecutive longitudinal digital screening mammograms^21^. The exclusion criteria were: we excluded patients without at least 1 year of screening follow-up, in line with the research^21^. Details about the distributions of the dataset are available in Supplementary Fig. 3. Although part of the patients did not have 10-year screening follow-up, we also leverage their known outcomes and images to supervise the model. Therefore, we keep 9133 patients consisting of 2562 BC patients who were biopsy-proven within 10 years and 6571 at intermediate risk who had at least 10 years of screening follow-up and did not receive a cancer diagnosis. All patients are randomly divided into training, validation, and test sets with a ratio of 7.5:1:1.5. Note that the split was made at the patient-level so that no image from any given patient would be in more than one partition of the dataset. The training, validation, and test sets include 6858, 919, and 1356 patients with 32,049, 4432, and 6311 examinations, respectively.

In our study, we collect the electronic medical records of the patients, which include radiology reports generated by radiologists after radiographic imaging, pathology reports after biopsy, and therapy reports from our hospital. We then further collected and constructed structured tables of risk factors and prognostic factors from these reports.

#### Risk factors

BC-relevant risk factors are already showing an essential role in both traditional^12,17^ and image-based DL methods^18,21^. Specifically, we obtain age, race, family history, menopausal status, and age of menarche from self-report. Additionally, genetic determinants, previous BC history, previous ovarian cancer history, BI-RADS final assessment scores, and breast density in the ACR category are also collected. The BI-RADS final assessment scores and breast density ACR grades are estimated by radiologists during clinical interpretation. BI-RADS final assessment scores include additional imaging required (BI-RADS 0), normal (BI-RADS 1), benign (BI-RADS 2), probably benign (BI-RADS 3), suspicious for malignancy (BI-RADS 4), highly suggestive of malignancy (BI-RADS 5), and known biopsy-proven malignancy (BI-RADS 6). The ACR class includes mostly composed of fatty tissue (ACR 1), scattered fibroglandular tissue (ACR 2), heterogeneously dense (ACR 3), and extremely dense (ACR 4). Images with missing densities are interpolated by nearest neighbor interpolation with reference to the density estimates of screening images from adjacent years of the patient. Because the weights of patients are missing, we did not calculate the body mass index (BMI). The distribution of clinical risk factors in the in-house dataset is shown in Table 1.

#### Prognostic factors

For patients with prior BC, our model also integrates the prognostic information collected from pathology reports and therapy reports of the initial BC. Specifically, following a previous BC recurrence risk prediction study (28), we collect the information of prior tumors, which includes location information of tumors, pathologic tumor (pT)-stage, pathologic node (pN)-stage, hormone receptor status (estrogen receptor (ER)- and progesterone receptor (PR)-status), anti-hormonal therapy, human epidermal growth factor receptor 2 (HER2-status), types of surgery, adjuvant chemotherapy, adjuvant radiation therapy, antibody therapy and whether or not pathologic complete response (pCR) was achieved. Previous studies indicate that pCR after neoadjuvant therapy is associated with improved event-free and overall survival^56^. These factors help to evaluate the prognosis of individuals who have already been diagnosed with BC, providing insights into the likelihood of disease progression or recurrence.

### Public dataset

To explore the performance of the proposed model on a public dataset, we also collect a comprehensive screening dataset called the Cohort of Screen-Aged Women (CSAW-CC) dataset^40^. The dataset is released by the Karolinska University Hospital after approval by the ethical review board of Stockholm, which waived the requirement for individual informed consent (EPN 2016/2600-31), and additional approval by the Ethical Review Authority of Sweden (EPM 2019-01946, EPM 2019-03638, EPM 2021-01030). This dataset contains anonymized mammograms from 873 women with primary BC and 7850 healthy women in the screening age range (40-74 years) examined in the period 2008 to 2015. Ground-truth information is sourced from the official dataset (https://snd.se/en/catalogue/dataset/2021-204-1). Each of the women was involved in one to five mammogram examinations. All screening mammograms are full-field digital mammograms and include four standard views acquired on Hologic equipment. Due to the lack of risk and prognostic factors, we only leverage the mammograms during further validation.

### Problem formulation

Risk calculation can be treated as a multi-class classification problem^18,21^, which is common in breast imaging, such as the classification of breast density^57^, the Breast Imaging Reporting and Data System (BI-RADS^58^) score^59^, the type of malignancy^3^, and the BC molecular subtype^60,61^. In this study, we first discretize the time interval between the screening mammograms of the patients and future cancer diagnoses into various 1-year time periods and treated each period as an independent class. Additionally, we also include one more class which defines whether the patients are cancer-free in the time interval. We define the sum of the probabilities for patients getting BC at each year and the probability of staying healthy throughout the total time interval to be equal to 1. To evaluate the overall risk of $j$ years, the probabilities of each year are summed together from the first year up to the ${j}_{{th}}$ year. The formulas are defined as follows Eq. 1:

1 $$Ris{k}_{j}=\mathop{\sum }\limits_{i=1}^{j}{y}_{i}=\mathop{\sum }\limits_{i=1}^{j}{Softmax\left(F\left(x\right)\right)}_{i}$$

where, ${y}_{i}$ means the predicted probability of an exam getting a BC diagnosis at ${i}_{{th}}$ year, which is calculated by inputting a sequence of mammograms and corrected risk factors (*m*) into the model *F* and using the $\mathrm{Softmax}$ function for the probability generalization. For example, to predict the ${Risk}$ of a patient getting BC within $j=2$ years from the checked images, it can be calculated as the sum of the probability of the first year and second year. Thus, the risk of a patient getting BC within 5 or 10 years from the available data can be calculated as the cumulative sum of the probability from the first year up to the 5th or 10th year. Importantly, the prediction results of our model can guarantee that the risk is monotonically increasing and self-consistent. This avoids the situation, that can occur with separately trained models, where long-term risk may be lower than short-term risk. Moreover, this formulation also learns the inherent relationship between risks at different time points. In this study, the model is trained to predict the risk of BC at each of the 15 years and is validated by predicting BC within a ten-year timeframe. Therefore, our model’s design allows for straightforward extension toward longer-term predictions (up to 15 years), provided that a sufficient amount of longer-term follow-up data becomes available.

### Architectural details

As shown in Fig. 1, the MTP-BCR method consists of the weights-shared image encoder (${\varphi }_{\mathrm{encoder}}$) connected to multi-level modules, including a side-specific (unilateral-based) module (${\varphi }_{\mathrm{unilateral}}$), exam-based module (${\varphi }_{\mathrm{exam}}$), and finally, a multi-time-point fusion module (${\varphi }_{\mathrm{fusion}}$) that combines with the inputted risk factors of patients. Moreover, to improve risk modeling performance and generalization, we also introduce multi-task learning, which could benefit from learning the domain-specific features from multiple BC risk-related tasks (detailed below).

#### Image encoder

We employ ImageNet pretrained ResNet-18, excluding the last full connection (FC), as the encoder (${\varphi }_{\mathrm{encoder}}$) to extract breast tissue features. The weights-shared encoders correspond to each image from the sequence of mammography exams. Each exam includes four images, including the craniocaudal (CC) view ($v={cc}$) and mediolateral oblique (MLO) view ($v={mlo}$) from the left ($l=\mathrm{left}$) and right ($l=\mathrm{right}$) side of the breast. Thus, as shown in Eq. 2, each input mammogram, ${x}_{v,\,l}^{t}$, from the six-time point ($t$) exams is represented as the high-dimensional locally feature vector ${\theta }_{v,\,l}^{t}$ with the size of $512\times 1$ by the encoder separately.

2 $${\theta }_{v,\,l}^{t}={\varphi }_{{encoder}}\left({x}_{v,\,l}^{t}\right),\,v\in \left\{{cc},{mlo}\right\},\,l\in \left\{{right},{left}\right\},\,t\in \left\{0,\,1,\,2,\,3,\,4,\,5\right\}$$

#### Side-specific prediction module

Ipsilateral CC and MLO views are different projection views of the same breast. Practically, they are combined to express the three-dimensional structure of the breast, which radiologists use to detect abnormalities. Moreover, most tumors only appear in one of the breasts^62^. In order to train the model to learn the three-dimensional structure of the breast fully and correspond to the previous left and right breast-specific tumor information, we concatenate ($\oplus$) the feature vectors of the ipsilateral view, combined with the side-specific prior tumor information (${\mathrm{tumor}}_{l}^{t}$) and then place a multi-layer perceptron (MLP) for side-based multi-task learning. The inclusion of tumor characteristics in the model is intended to enhance its predictive capability of recurrence BC risk. When the lateral information is unknown or predicts a primary tumor, the vector of the inputting tumor characteristics will be replaced by minus-one-filled placeholders. The MLP layer includes two FC layers with an input size of 1152 for the first FC layer and 512 output units each. A dropout layer with a rate of 0.5 between the two FC layers. Therefore, as shown in Eq. 3 features of the ipsilateral CC and MLO views and outputs a vector (${\varepsilon }_{l}^{t}$) with the size of $512\times 1$ representing the unilateral-based breast features.

3 $${\varepsilon }_{l}^{t}={\varphi }_{{unilateral}}\left({{tumor}}_{l}^{t}{\oplus \theta }_{l,\,v={cc}}^{t}{\oplus \theta }_{l,\,v={mlo}}^{t}\right),\,l\in \left\{right{,}left\right\},\,t\in \left\{0,\,1,\,2,\,3,\,4,\,5\right\}$$

#### Exam-based prediction module

For a similar purpose, we also need to combine the information based on bilateral breasts to predict the patient-level risk. As in Eq. 4, we then concatenate the feature vectors from the output bilateral breasts and feed them to another MLP layer with the same structure for exam-based multi-task learning. Also, a size of $512\times 1$ vector feature (${\delta }^{t}$), which combines the features from the right and left breast, represents the global information of a four-view exam.

4 $${\delta }^{t}={\varphi }_{exam}\left({\varepsilon }_{l=right}^{t}\oplus {\varepsilon }_{l=left}^{t}\right),\,t\in \left\{0,\,1,\,2,\,3,\,4,\,5\right\}$$

#### Multi-time point fusion model

Tor learning the risk development pattern from the longitudinal screening mammograms, five history exams before the current exam are randomly selected as the reference for the comparison by the multi-time point fusion model. The current exam refers to the target exam for which we access future BC risk. A sequence of mammography exams serves as references along with the time intervals (${i}_{0},\,{i}_{1},\,\cdots ,\,{i}_{5}$) to the current exam and are combined with the risk factors to predict the future likelihood of BC occurring after the current mammograms. Inspired by the research^28^, we leverage a sequence/time-aware transformer learning^63^ to capture features about the temporal relations between multiple mammograms, which aims to disentangle the risk-relevant changing patterns from the normal breast tissue changing patterns. For embedding the spatiotemporal relationships of past-current exams into the continuous latent space, we employ the Continuous Position Embedding (CPE) method^28^, which computes time continuous embedding ${e}^{t}$ to condition the image features. Not that, to avoid ignoring local information of images during multi-time exam comparison, we combine both the local image features ${\theta }_{v,{l}}^{t}$, and global features ${\delta }^{t}$. For patients without five history records for references, we select all history records and then mute the missing data by filling in 0. While, with the same aim as the data augmentation, we randomly drop a subset of exams in the reference sequence to improve model robustness and avoid overfitting. The fusion model also includes the patient risk factors (${riskf}$). Subsequently, a fused feature ($\tau$, a ${\rm{vector}}$ size of $640\times 1$) is obtained for the final multi-time fused-based multi-task learning, representing the patient’s multi-time point screening information.

5 $$\tau ={\varphi }_{\mathrm{fusion}}({{riskf}\oplus e}^{t}{\oplus \delta }^{t}{\oplus \theta }_{l,\,v}^{t})$$

#### Multi-task classifier for multi-level learning (side-based, exam-based, and multi-time fused prediction)

Multi-task learning is a data-efficient method that can help improve performance and potentially enhance robustness and generalization. In this study, we utilize multi-task learning to constrain the risk model to learn rich information from the mammograms and the input information for enhancing the ability of BC risk-related characterization and avoiding overfitting, as shown in Fig. 1E. For example, the multi-task includes the prediction of the risk factors (age and density), future BC risk, location, and types. More importantly, half of the examinations with a prior diagnosis of BC, thus related to the recurrence BC risk prediction. Accordingly, we also leverage the model to predict BC history and related features (tumor location, tumor subtype, therapy status, and so on) in multi-task learning. This multi-task classification is used to facilitate multi-level learning across side-specific, exam-based, and multi-time-point modules.

Specifically, a single-time single-side-based multi-task classifier is trained to predict risk, history, location (C50 code) of prior tumor and future tumor, type (recurrence or primary) of prior tumor and future tumor, PCR status of the prior tumor, post TNM stage of the prior tumor, ER, PR, Her2 of the future tumor. Single-time exam-based multi-task classifier includes the predictions of risk, history, age, density, BI-RADS, manufactory, location (C50 code) of prior tumor and future tumor, laterality (left or right) of prior tumor and future tumor, type (recurrence or primary) of prior tumor and future tumor, PCR status of the prior tumor, post TNM stage of the prior tumor, ER, PR, Her2 of the future tumor. We leverage the manufacturer information that is mainly proposed to help the model learn the texture of the breast in the image. Variations in imaging devices or settings could impact the appearance of abnormalities in the mammogram. For the final multi-time fused classifier, we focus on both unilateral specific BC risk and patient-level overall risk. The final multi-time fused classifier includes the predictions of the risk of unilateral breast, risk of patient, history, future tumor type (recurrence or primary), Age, density, BI-RADS, future tumor location (C50 code), laterality (left or right) of prior tumor and future tumor. Note that we only leverage these labels during the training phases. The binary cross entropy (BCE) for risk prediction and cross entropy (CE) loss for other predictions are calculated. For all three classifiers, we mainly focus on the task of risk prediction; thus, risk-specific tasks have five times higher weight than other tasks during the training. For total loss computing, Eq. 6, we also allocate weight ${w}_{\mathrm{fusion}}$ = 1 to the final multi-time point fused classifier, five times higher than the other two classifiers$\,({w}_{\mathrm{side}}=0.2,\,{w}_{\mathrm{exam}}=0.2)$. We choose the weights of loss after the hyperparameter search.

6 $${L}_{total}={L}_{side}\times {w}_{side}+{L}_{exam}\times {w}_{exam}+{L}_{fusion}\times {w}_{fusion}$$

### Implementation details

In this study, we utilize ResNet-18 as the backbone of all our methods, initialized with ImageNet pretrained weights. The implementation is carried out in PyTorch (version 1.12.1) employing consistent training strategies across all models. We use the Adam^64^ optimizer and a rate of 0.5 for dropout^65^ after every fully connected layer. The models are trained for 20 epochs with a batch size of 8 and an initial learning rate of 10^−4^. The learning rate is decayed by a factor of 10 every five epochs. The selection of the best models for each method is based on the highest mean AUC performance within the 1- to 10-year risk prediction index on the validation set. The experiments are performed on a Quadro A6000 GPU (48GB). The source code is available at https://github.com/Netherlands-Cancer-Institute/MTP-BCR. Mammograms with a common DICOM format are pre-processed before being fed into the model. First, we convert the images into 16-bit PNG format and segment the whole breast region to exclude the background. Then, to unify the size of all images, we zero-pad and resize images to 512 by 1024 pixels while retaining the relative scale and aspect ratio. Finally, the image is normalized using the min-max method. We also employ standard data augmentation techniques (i.e., random flip, brightness, and contrast) during training for model robustness and overfitting prevention.

### Ablation experiments

To optimize the design of the MTP-BCR model, we conducted several ablation studies. The first study aimed to evaluate the impact of our proposed learning strategies: multi-task, multi-level, and multi-time-point learning, alongside the integration of traditional risk factors, on the model’s ability to extract risk-related features. The second study focused on assessing the performance variations of the MTP-BCR method when trained with different numbers of prior reference exams, thereby exploring the influence of temporal data on risk prediction accuracy. Additionally, to determine whether training on the full dataset enhances the model’s capability in comprehensively capturing BC risk development, early tumor signs, and recurrence risk, we performed an experiment comparing the outcomes of training from scratch on three different datasets: the full dataset, a primary cancer dataset (excluding mammograms from patients with a prior BC diagnosis), and a recurrence dataset (only including mammograms from patients with a history of BC).

### Competitive methods

Traditional risk models: the mammogram-based in-house dataset is coupled to patient-derived classical risk factors that can be used in the clinical Breast Cancer Surveillance Consortium version 2 (BCSC, https://tools.bcsc-scc.org/BC5yearRisk/). The distribution of clinical risk factors is shown in Supplemental Supplementary Table 2. The BCSC model can estimate 5-year and 10-year BC risk based on risk factors but requires excluding patients following exclusion criteria (previous diagnosis of BC, younger than age 35 or older than age 74, or missing density estimates). Although studies have shown that image-based DL risk models outperform traditional risk models in 5-year risk assessments^21,25,26^, the potential advantages of the former still need to be explored in longer-term 10-year risk assessments. Thus, we compare our model with not only the traditional BCSC 1-year and 5-year risk models but also with the BCSC 10-year risk model. Specifically, we calculated the AUC of the 1-year BC risk through the BCSC model-predicted 1-year risk probabilities. The AUC results of 2-year to 5-year risk were calculated by leveraging the BCSC model’s 5-year risk probabilities. For the AUC results of the longer-term risk (6-year to 10-year risk), we used the 10-year risk probabilities provided by the BCSC model. In addition to the BCSC model, we also trained a machine learning-based risk prediction model (Baseline-risk factors), which only leverages collected risk factors. We built this risk-factor-only model by leveraging support vector machine (SVM) techniques. Moreover, to validate the models’ ability for recurrence risk prediction, we compare our model with a machine learning-based recurrence risk prediction model that leverages both collected prognostic risk factors and standard risk factors. The recurrence risk model (Baseline-Recurrence) is also based on SVM techniques, which is one of the most common machine learning techniques in the BC recurrence risk prediction task^39^.

#### Mammogram-based DL methods

To demonstrate superior risk prediction performance, we engage in comparison with an image-based baseline DL method (STP-Baseline), characterized by the single-time point mammogram-based DL method without the incorporation of any risk factors. This baseline method combines four views of mammograms using late-fusion for risk prediction, which is based on a similar strategy to previous studies^18,66^. To explore the added value of the inclusion of patient-based risk factors, we also built a multi-time point model without risk factors for comparison. We also compare our method to the state-of-the-art (SOTA) DL-based risk prediction method^21^, which leverages a transformer to combine multiple representations of each view of a single time point exam (referenced as STP-transformer in this paper). This mammogram-based risk model can predict 5-year risk at multiple time points and outperforms traditional models. Moreover, this model includes a pretrained risk factor predictor that allows it to be beneficial from predicted risk factors when missing them^21^. In this study, we leveraged the provided weights and then retrained the STP-Transformer model on the in-house training dataset to alleviate the impact of domain shift. We conduct a hyperparameter search to retrain and select the model with the best concordance index (C-index) on the validation set. For a fair comparison to validate that the advantages are from the multi-time point design of the risk model, we keep the same input size (1024 × 512) for all risk models.

Similar to the research^21^, to evaluate the performance of our method on BC detection, we also compare our model with the retrospective BI-RADS scores of the radiologist and the globally aware multiple instance (GMIC) model^32^. The GMIC is another recent SOTA DL model that focuses on detecting BC within three months, and some studies also show its potential for predicting BC risk^21^. The pretrained model is obtained from the public GitHub repository (https://www.github.com/nyukat/gmic). We collect the ensembled predictions from the five pretrained models (referenced as STP-Detection in this paper).

### Evaluation metrics and statistical analysis

In this study, the tasks of prediction of 1- to 10-year risk are categorical classification tasks, in which positive samples are the patients diagnosed with BC within 1 to 10 years, while negative samples are women who stayed healthy for at least 1 to 10 years of screening follow-ups. Evaluation of the distribution of participants’ information is performed using the Wilcoxon rank-sum test or Pearson’s χ^2^ test. The performances of the different methods are evaluated by the area under the receiver operating characteristic curve (AUC, calculated by scikit-learn, version: 1.1.2, https://scikit-learn.org). To generally evaluate AUCs across all times (from 1- to 10-year risk), Uno’s C-index^67^ is calculated using scikit-survival (version 0.18.0, https://scikit-survival.readthedocs.io/en/stable/). Besides, to investigate how much age affects the model’s performance, we also calculate the age-adjusted AUC (aAUC) following a previous study^29^. The method is based on the covariate-adjusted ROC (AROC), which is defined by res. ^68–70^. These measures were implemented to mitigate the potential confounding effects of age on the reported results. The 95% confidence intervals (CI) of AUC and C-index matrices are estimated by bootstrapping with 1000 bootstraps for each measure. Statistical significance among different methods is assessed using DeLong’s test^71^, with the significant level predefined as *P* < 0.05. Additionally, Kaplan–Meier (KM) survival curves are used to visualize the risk stratification abilities of the risk models between different risk groups.

## Supplementary information

SUPPLEMENTAL MATERIAL

TRIPOD-AI checklist

## Data Availability

All data supporting the findings can be provided upon reasonable request to the corresponding author for non-commercial and academic purposes. The public CSAW-CC dataset is obtained from https://snd.gu.se/en/catalogue/dataset/2021-204-1/1. All data associated with this study are present in the paper or the Supplementary Materials. Source data are provided in this paper.

## Code availability

The source codes related to training and developing the deep learning models are available on https://github.com/Netherlands-Cancer-Institute/MTP-BCR.
