# Supplementary material for: Predicting short- to long-term breast cancer risk from longitudinal mammographic screening history
Source: NPJ Breast Cancer. 2025 Oct 29;11:118. doi: 10.1038/s41523-025-00831-x (PMC12572377; doi:10.1038/s41523-025-00831-x)
Supplement: Supplementary file 1 — SUPPLEMENTAL MATERIAL [file 41523_2025_831_MOESM1_ESM.pdf]

Supplemental information

Predicting short- to long-term breast cancer risk from longitudinal mammographic screening history

Xin Wang, Tao Tan,* Yuan Gao, Ruisheng Su, Jonas Teuwen, Jaap Kroes, Tianyu Zhang, Anna D'Angelo, Luyi Han, Caroline A. Drukker, Marjanka K. Schmidt, Regina Beets-Tan, Nico Karssemeijer, Ritse Mann

This PDF file includes:

Supplementary Figs. 1 to 4

Supplementary Tables 1 to 18

Details of inhouse dataset

Supplementary Fig.1. Overview of retrospective patients' trajectory for the inhouse dataset.

Supplementary Fig.2. The flowchart of dataset collection.

Supplementary Fig.3. Details of the inhouse dataset.

Supplementary Table 1. Number of examinations per cancer type in the Inhouse dataset.

Supplementary Table 2. Detailed Demographics of the training, validation, and test set of the inhouse dataset

Supplementary Table 3. Detailed demographics for three cohorts of the Inhouse dataset.

Supplementary Table 4. Detailed demographics for two primary screening cohorts of the Inhouse dataset.

Supplementary results of 10-year BC risk prediction

Ablation studies:

Supplementary Table 5. Ablation studies on 10-year risk dataset.

Supplementary Table 6. Comparison of BCR-MTP method for 10 year risk predictions using different number of time point exams on full inhouse test set

Supplementary Table 7. C-index performance of MTP-BCR models which trained on different training datasets.

Results of comparison of 10-year risk prediction on primary BC population:

Supplementary Table 8. Comparison of 10-year risk predictions on primary BC group of the inhouse test set (only for women who were completely scored across the BCSC model).

Supplementary Table 9. Comparison of 10-year risk predictions on primary screening cohorts (only for women who were completely scored across the BCSC model).

Supplementary Table 10. The comparison of risk stratification ability on the external CSAW-CC dataset.

Supplementary Table 11. Comparison of 10-year risk predictions with MIRAI on inhosue test set, two screening cohorts, and CSAW-CC dataset.

Supplementary Fig.4. 1- to 10-year AUC results of risk prediction excluding the breast cancer cases in 90 days-9 years.

Results of comparison of 10-year risk prediction on recurrence cohort:

Supplementary Table 12. Comparison of 10-year risk predictions on recurrence cohort

Subgroup analysis:

Supplementary Table 13. C-index comparison of 10-year risk prediction in different sub-groups of inhouse dataset.

Supplementary Table 14. Comparison of risk prediction in different sub-groups of the external CSAW dataset

Results of adjusted AUC (aAUC):

Supplementary Table 15. Comparison of 10-year risk predictions based on age-adjusted aAUC on inhouse test set.

Supplementary Table 16. Comparison of 10-year risk predictions based on age-adjusted aAUC on two screening cohorts.

Supplementary Table 17. Comparison of 10-year risk predictions based on age-adjusted aAUC on two primary screening cohorts (only for women who were completely scored across the BCSC model).

Supplementary Table 18. Comparison of 10-year risk predictions based on age-adjusted aAUC on recurrence cohorts.

Results of comparison of 10-year risk prediction on patient-level evaluation:

Supplementary Table 19. Comparison of 10-year risk predictions on inhouse test set, three cohorts, and CSAW-CC dataset using patient-level evaluation (one exam per patient).

Details of inhouse dataset

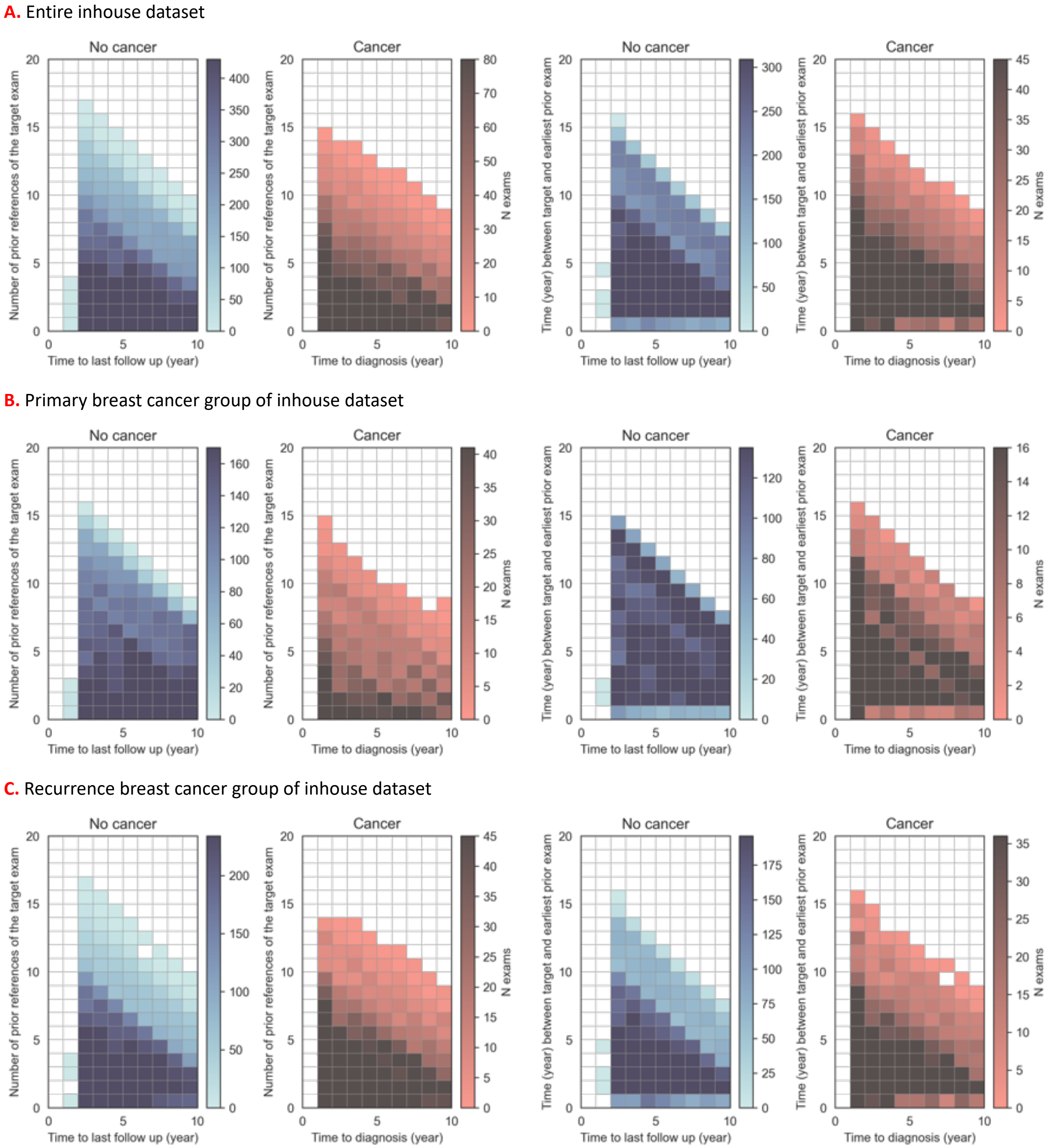

Supplementary Fig.1. Overview of retrospective patients' trajectory for the inhouse dataset. A. graph of trajectories for the entire inhouse dataset; **B.** trajectory graph for primary breast cancer group of the inhouse dataset; **C.** trajectory graph for recurrence breast cancer group of the inhouse dataset.

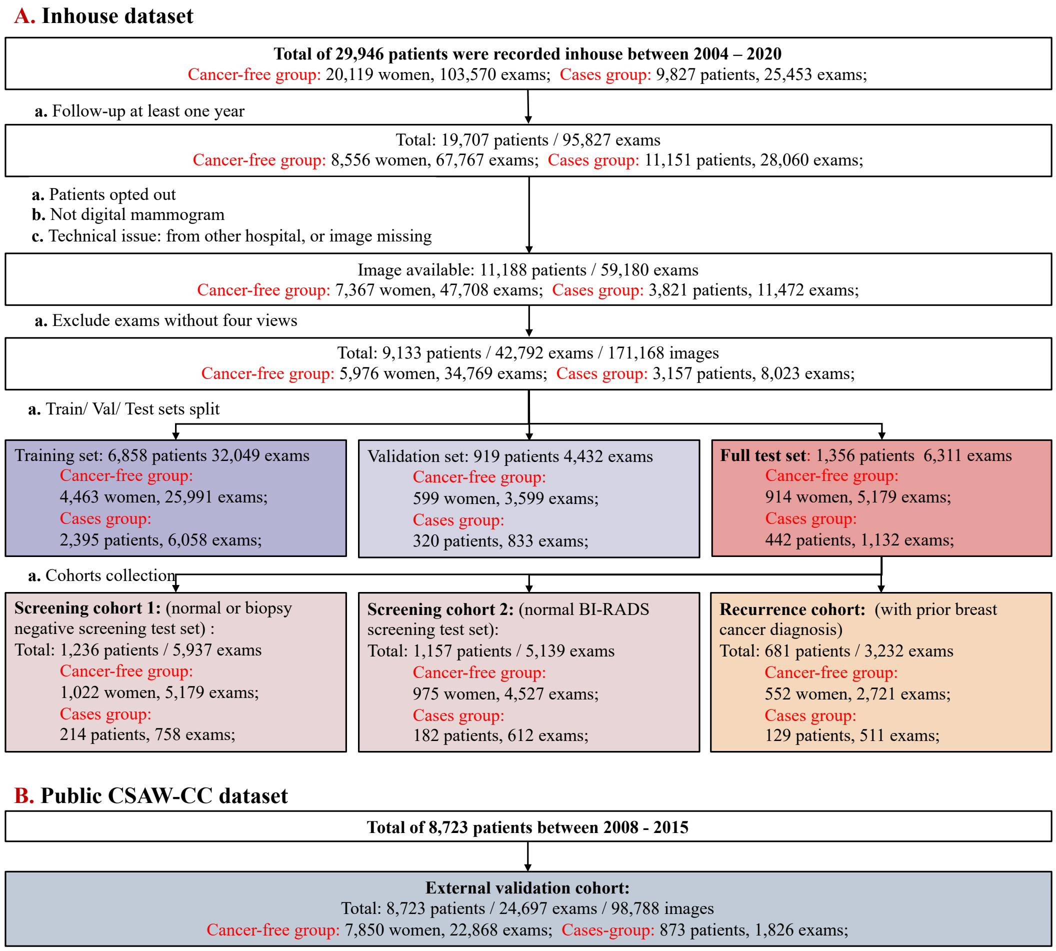

Supplementary Fig.2. The flowchart of dataset collection. A. Inhouse mammogram dataset collection for 10-year risk prediction Inhouse dataset from Netherland Cancer Institute. **B.** Public dataset collection. CSAW-CC, Cohort of Screen-age Women - Case control.

A

Exams with at least X years of screening followup

	All			Training Set			Validation Set			Test Set		
	Full	Biopsy Negative	Normal BI-RADS	Full	Biopsy Negative	Normal BI-RADS	Full	Biopsy Negative	Normal BI-RADS	Full	Biopsy Negative	Normal BI-RADS
**X=1**	**42,792**	**40,024**	**34,636**	**32,049**	**29,932**	**25,887**	**4,432**	**4,155**	**3,610**	**6,311**	**5,937**	**5,139**
X=2	37,943	35,175	30,181	28,417	26,300	22,574	3,956	3,679	3,162	5,570	5,196	4,445
X=3	33,396	30,628	25,997	25,057	22,940	19,480	3,466	3,189	2,717	4,873	4,499	3,800
X=4	29,348	26,580	22,265	22,040	19,923	16,708	3,043	2,766	2,327	4,265	3,891	3,230
**X=5**	**25,472**	**22,704**	**18,720**	**19,156**	**17,039**	**14,068**	**2,622**	**2,345**	**1,943**	**3,694**	**3,320**	**2,709**
X=6	22,304	19,536	15,883	16,782	14,665	11,942	2,295	2,018	1,648	3,227	2,853	2,293
X=7	19,438	16,670	13,359	14,649	12,532	10,071	1,985	1,708	1,369	2,804	2,430	1,919
X=8	17,019	14,251	11,280	12,832	10,715	8,507	1,731	1,454	1,150	2,456	2,082	1,623
X=9	14,861	12,093	9,444	11,210	9,093	7,116	1,526	1,249	980	2,125	1,751	1,348
X=10	13,177	10,409	8,044	9,951	7,834	6,066	1,343	1,066	827	1,883	1,509	1,151
X=11	11,800	9,032	6,936	8,918	6,801	5,231	1,204	927	718	1,678	1,304	987
X=12	10,726	7,958	6,120	8,119	6,002	4,617	1,086	809	631	1,521	1,147	872
X=13	9,851	7,083	5,486	7,456	5,339	4,135	990	713	557	1,405	1,031	794
X=14	9,200	6,432	5,038	6,968	4,851	3,798	922	645	510	1,310	936	730
X=15	8,757	5,989	4,766	6,621	4,504	3,588	880	603	484	1,256	882	694

B

Exams followed by a cancer diagnosis within X years

	All			Training Set			Validation Set			Test Set		
	Full	Biopsy Negative	Normal BI-RADS	Full	Biopsy Negative	Normal BI-RADS	Full	Biopsy Negative	Normal BI-RADS	Full	Biopsy Negative	Normal BI-RADS
**X=1**	**3,742**	**974**	**708**	**2,853**	**736**	**527**	**378**	**101**	**79**	**511**	**137**	**102**
X=2	4,461	1,693	1,316	3,393	1,276	986	460	183	150	608	234	180
X=3	5,152	2,384	1,899	3,918	1,801	1,430	531	254	207	703	329	262
X=4	5,745	2,977	2,398	4,360	2,243	1,798	597	320	265	788	414	335
**X=5**	**6,270**	**3,502**	**2,841**	**4,755**	**2,638**	**2,132**	**646**	**369**	**305**	**869**	**495**	**404**
X=6	6,721	3,953	3,220	5,090	2,973	2,414	699	422	348	932	558	458
X=7	7,147	4,379	3,562	5,401	3,284	2,667	745	468	384	1,001	627	511
X=8	7,498	4,730	3,839	5,665	3,548	2,877	784	507	411	1,049	675	551
X=9	7,790	5,022	4,074	5,889	3,772	3,061	812	535	433	1,089	715	580
X=10	8,023	5,255	4,253	6,058	3,941	3,193	833	556	448	1,132	758	612
X=11	8,208	5,440	4,394	6,199	4,082	3,303	845	568	458	1,164	790	633
X=12	8,352	5,584	4,496	6,308	4,191	3,381	854	577	463	1,190	816	652
X=13	8,460	5,692	4,574	6,385	4,268	3,437	861	584	469	1,214	840	668
X=14	8,530	5,762	4,619	6,440	4,323	3,472	862	585	470	1,228	854	677
X=15	8,566	5,798	4,646	6,471	4,354	3,494	863	586	471	1,232	858	681

C

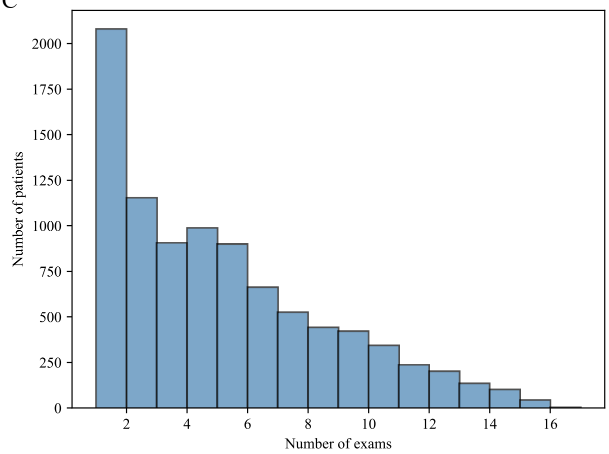

D

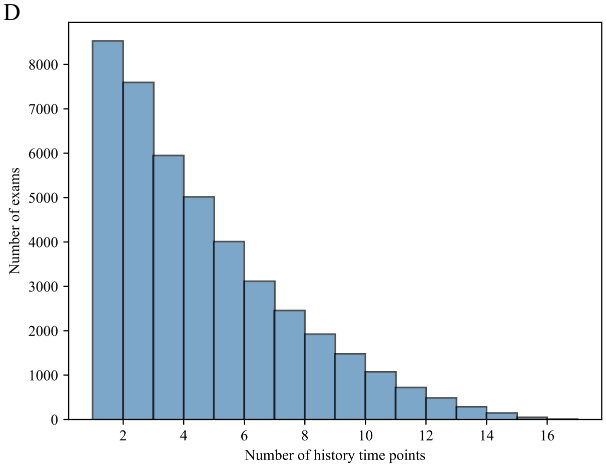

Supplementary Fig.3. Details of the inhouse dataset. A. Exams with at least X years of screening follow-up. **B.** Exams followed by a cancer diagnosis within X years. **C.** Histogram of the number of inhouse patients with different numbers of mammography records from 2004-2020. **D.** Histogram of the number of mammography examinations with different number of history time point records from 2004-2020.

Supplementary Table 1. Number of examinations per cancer type in the Inhouse dataset

Receptor subtype of tumor	Number of Exams				
	Inhouse datset	Test set	Screening cohort 1	Screening cohort 2	Recurrence cohort
ER+	4,476 (82.80%)	649 (81.53%)	151 (79.89%)	126 (76.83%)	283 (86.81%)
ER-	930 (17.20%)	147 (18.47%)	38 (20.11%)	38 (23.17%)	43 (13.19%)
PR+	3,519 (65.09%)	508 (63.82%)	131 (69.31%)	109 (66.46%)	223 (68.40%)
PR-	1,887 (34.91%)	288 (36.18%)	58 (30.69%)	55 (33.54%)	103 (31.60%)
Her2+	768 (14.21%)	144 (18.09%)	29 (15.34%)	24 (14.63%)	60 (18.40%)
Her2-	4,638 (85.79%)	652 (81.91%)	160 (84.66%)	140 (85.37%)	266 (81.60%)

Supplementary Table 2. Detailed Demographics of the training, validation, and test set of the inhouse dataset.

	Inhouse Dataset					
	Training Set		Development Set		Test Set	
	Total (N=32,049)	Cancer (N=6,058)	Total (N=4,432)	Cancer (N=833)	Total (N=6,311)	Cancer (N=1,132)
**Age:**						
<40	3,722 (11.61%)	506 (8.35%)	599 (13.52%)	88 (10.56%)	644 (10.20%)	81 (7.16%)
40-50	8,012 (25.00%)	1,339 (22.10%)	1,248 (28.16%)	189 (22.69%)	1,516 (24.02%)	265 (23.41%)
50-60	9,204 (28.72%)	1,813 (29.93%)	1,205 (27.19%)	193 (23.17%)	1,947 (30.85%)	333 (29.42%)
60-70	6,764 (21.11%)	1,551 (25.60%)	815 (18.39%)	247 (29.65%)	1,363 (21.60%)	316 (27.92%)
70-80	2,934 (9.15%)	595 (9.82%)	350 (7.90%)	78 (9.36%)	558 (8.84%)	99 (8.75%)
>80	318 (0.99%)	119 (1.96%)	46 (1.04%)	21 (2.52%)	67 (1.06%)	18 (1.59%)
Unknown	1,095 (3.42%)	135 (2.23%)	169 (3.81%)	17 (2.04%)	216 (3.42%)	20 (1.77%)
**BI-RADS：**						
BI-RADS 0	264 (0.82%)	49 (0.81%)	45 (1.02%)	12 (1.44%)	56 (0.89%)	8 (0.71%)
BI-RADS 1	4,856 (15.15%)	365 (6.03%)	736 (16.61%)	62 (7.44%)	824 (13.06%)	76 (6.71%)
BI-RADS 2	20,767 (64.80%)	2,779 (45.87%)	2,829 (63.83%)	374 (44.90%)	4,259 (67.49%)	528 (46.64%)
BI-RADS 3	1,044 (3.26%)	286 (4.72%)	154 (3.47%)	48 (5.76%)	185 (2.93%)	58 (5.12%)
BI-RADS 4	674 (2.10%)	497 (8.20%)	69 (1.56%)	50 (6.00%)	115 (1.82%)	81 (7.16%)
BI-RADS 5	633 (1.98%)	619 (10.22%)	92 (2.08%)	89 (10.68%)	124 (1.96%)	120 (10.60%)
BI-RADS 6	696 (2.17%)	696 (11.49%)	95 (2.14%)	95 (11.40%)	126 (2.00%)	126 (11.13%)
None	3,115 (9.72%)	767 (12.66%)	412 (9.30%)	103 (12.36%)	622 (9.86%)	135 (11.93%)
**Density:**						
ACR 1	2,029 (6.33%)	336 (5.55%)	259 (5.84%)	53 (6.36%)	407 (6.45%)	56 (4.95%)
ACR 2	18,698 (58.34%)	3,725 (61.49%)	2,504 (56.50%)	493 (59.18%)	3,790 (60.05%)	723 (63.87%)
ACR 3	8,420 (26.27%)	1,392 (22.98%)	1,207 (27.23%)	195 (23.41%)	1,603 (25.40%)	258 (22.79%)
ACR 4	2,742 (8.56%)	509 (8.40%)	439 (9.91%)	87 (10.44%)	479 (7.59%)	77 (6.80%)
Unknown	160 (0.50%)	96 (1.58%)	23 (0.52%)	5 (0.60%)	32 (0.51%)	18 (1.59%)
**Race:**						
White	9,043 (28.22%)	1,566 (25.85%)	1,348 (30.42%)	228 (27.37%)	1,875 (29.71%)	339 (29.95%)
African	116 (0.36%)	21 (0.35%)	9 (0.20%)	0 (0.00%)	35 (0.55%)	4 (0.35%)
Asian	221 (0.69%)	24 (0.40%)	36 (0.81%)	0 (0.00%)	39 (0.62%)	7 (0.62%)
Other Race	66 (0.21%)	4 (0.07%)	5 (0.11%)	5 (0.60%)	35 (0.55%)	7 (0.62%)
Unknown	22,603 (70.53%)	4,443 (73.34%)	3,034 (68.46%)	600 (72.03%)	4,327 (68.56%)	775 (68.46%)
**Gene mutation (Brca 1/2, Chek2, Tp53):**						
Positive	193 (0.60%)	175 (2.89%)	48 (1.08%)	42 (5.04%)	41 (0.65%)	34 (3.00%)
Negative	689 (2.15%)	394 (6.50%)	95 (2.14%)	55 (6.60%)	158 (2.50%)	75 (6.63%)
Unknown	31,167 (97.25%)	5,489 (90.61%)	4,289 (96.77%)	736 (88.36%)	6,112 (96.85%)	1,023 (90.37%)
**Menopausal Status:**						
Pre-menopausal	4,686 (14.62%)	688 (11.36%)	762 (17.19%)	125 (15.01%)	829 (13.14%)	131 (11.57%)
Peri-menopausal & Unknown	15,880 (49.55%)	2,740 (45.23%)	2,275 (51.33%)	315 (37.82%)	3,236 (51.28%)	511 (45.14%)
Post- menopausal	11,483 (35.83%)	2,630 (43.41%)	1,395 (31.48%)	393 (47.18%)	2,246 (35.59%)	490 (43.29%)
**Personal History:**						
Breast Cancer Positive	15,581 (48.62%)	2,856 (47.14%)	1,992 (44.95%)	380 (45.62%)	3,232 (51.21%)	511 (45.14%)
Breast Cancer Negative	16,468 (51.38%)	3,202 (52.86%)	2,440 (55.05%)	453 (54.38%)	3,079 (48.79%)	621 (54.86%)
Ovarian Cancer Positive	376 (1.17%)	95 (1.57%)	43 (0.97%)	11 (1.32%)	87 (1.38%)	32 (2.83%)
Ovarian Cancer Negative	31,673 (98.83%)	5,963 (98.43%)	4,389 (99.03%)	822 (98.68%)	6,224 (98.62%)	1,100 (97.17%)
**Menarche Age：**						
<12	696 (2.17%)	75 (1.24%)	138 (3.11%)	34 (4.08%)	98 (1.55%)	4 (0.35%)
12-15	4,019 (12.54%)	563 (9.29%)	637 (14.37%)	42 (5.04%)	846 (13.41%)	153 (13.52%)
>15	173 (0.54%)	13 (0.21%)	41 (0.93%)	9 (1.08%)	37 (0.59%)	6 (0.53%)
Unknown	27,161 (84.75%)	5,407 (89.25%)	3,616 (81.59%)	748 (89.80%)	5,330 (84.46%)	969 (85.60%)
**Family History：**						
Breast Cancer Positive	23,174 (72.31%)	5,174 (85.41%)	3,102 (69.99%)	706 (84.75%)	4,602 (72.92%)	972 (85.87%)
Breast Cancer Negative	8,875 (27.69%)	884 (14.59%)	1,330 (30.01%)	127 (15.25%)	1,709 (27.08%)	160 (14.13%)
Ovarian Cancer Positive	1,945 (6.07%)	403 (6.65%)	266 (6.00%)	39 (4.68%)	308 (4.88%)	68 (6.01%)
Ovarian Cancer Negative	30,104 (93.93%)	5,655 (93.35%)	4,166 (94.00%)	794 (95.32%)	6,003 (95.12%)	1,064 (93.99%)
**Manufacturer：**						
Hologic Selenia	5,264 (16.42%)	1,324 (21.86%)	683 (15.41%)	172 (20.65%)	1,008 (15.97%)	239 (21.11%)
Lorad Selenia	8,690 (27.11%)	2,105 (34.75%)	1,217 (27.46%)	276 (33.13%)	1,708 (27.06%)	364 (32.16%)
Selenia Dimensions	17,658 (55.10%)	2,302 (38.00%)	2,478 (55.91%)	343 (41.18%)	3,513 (55.66%)	465 (41.08%)
**Time to cancer, years:**						
0-1	2,853 (8.90%)	2,853 (47.09%)	378 (8.53%)	378 (45.38%)	511 (8.10%)	511 (45.14%)
1-2	540 (1.68%)	540 (8.91%)	82 (1.85%)	82 (9.84%)	97 (1.54%)	97 (8.57%)
2-3	525 (1.64%)	525 (8.67%)	71 (1.60%)	71 (8.52%)	95 (1.51%)	95 (8.39%)
3-4	442 (1.38%)	442 (7.30%)	66 (1.49%)	66 (7.92%)	85 (1.35%)	85 (7.51%)
4-5	395 (1.23%)	395 (6.52%)	49 (1.11%)	49 (5.88%)	81 (1.28%)	81 (7.16%)
5-6	335 (1.05%)	335 (5.53%)	53 (1.20%)	53 (6.36%)	63 (1.00%)	63 (5.57%)
6-7	311 (0.97%)	311 (5.13%)	46 (1.04%)	46 (5.52%)	69 (1.09%)	69 (6.10%)
7-8	264 (0.82%)	264 (4.36%)	39 (0.88%)	39 (4.68%)	48 (0.76%)	48 (4.24%)
8-9	224 (0.70%)	224 (3.70%)	28 (0.63%)	28 (3.36%)	40 (0.63%)	40 (3.53%)
9-10	169 (0.53%)	169 (2.79%)	21 (0.47%)	21 (2.52%)	43 (0.68%)	43 (3.80%)

Supplementary Table 3. Detailed demographics for three cohorts of the Inhouse dataset.

	Inhouse Dataset					
	Screening cohort 1		Screening cohort 2		Recurrence cohort	
	Total (N=5,937)	Cancer (N=758)	Total (N=5,139)	Cancer (N=612)	Total (N=3,232)	Cancer (N=511)
**Age:**						
<40	609 (10.26%)	46 (6.07%)	517 (10.06%)	39 (6.37%)	113 (3.50%)	11 (2.15%)
40-50	1,420 (23.92%)	169 (22.30%)	1,213 (23.60%)	132 (21.57%)	450 (13.92%)	77 (15.07%)
50-60	1,838 (30.96%)	224 (29.55%)	1,616 (31.45%)	190 (31.05%)	1,064 (32.92%)	152 (29.75%)
60-70	1,275 (21.48%)	228 (30.08%)	1,079 (21.00%)	181 (29.58%)	947 (29.30%)	193 (37.77%)
70-80	524 (8.83%)	65 (8.58%)	456 (8.87%)	51 (8.33%)	451 (13.95%)	56 (10.96%)
>80	56 (0.94%)	7 (0.92%)	49 (0.95%)	4 (0.65%)	56 (1.73%)	8 (1.57%)
Unknown	215 (3.62%)	19 (2.51%)	209 (4.07%)	15 (2.45%)	151 (4.67%)	14 (2.74%)
**BI-RADS：**						
BI-RADS 0	50 (0.84%)	2 (0.26%)	56 (1.09%)	8 (1.31%)	21 (0.65%)	4 (0.78%)
BI-RADS 1	824 (13.88%)	76 (10.03%)	824 (16.03%)	76 (12.42%)	31 (0.96%)	9 (1.76%)
BI-RADS 2	4,259 (71.74%)	528 (69.66%)	4,259 (82.88%)	528 (86.27%)	2,760 (85.40%)	367 (71.82%)
BI-RADS 3	156 (2.63%)	29 (3.83%)	0 (0.00%)	0 (0.00%)	53 (1.64%)	17 (3.33%)
BI-RADS 4	43 (0.72%)	9 (1.19%)	0 (0.00%)	0 (0.00%)	32 (0.99%)	21 (4.11%)
BI-RADS 5	4 (0.07%)	0 (0.00%)	0 (0.00%)	0 (0.00%)	13 (0.40%)	10 (1.96%)
BI-RADS 6	0 (0.00%)	0 (0.00%)	0 (0.00%)	0 (0.00%)	18 (0.56%)	18 (3.52%)
Unknown	32 (0.54%)	32 (4.22%)	0 (0.00%)	0 (0.00%)	304 (9.41%)	65 (12.72%)
**Density:**						
ACR 1	392 (6.60%)	41 (5.41%)	369 (7.18%)	37 (6.05%)	253 (7.83%)	35 (6.85%)
ACR 2	3,549 (59.78%)	482 (63.59%)	2,997 (58.32%)	378 (61.76%)	2,140 (66.21%)	360 (70.45%)
ACR 3	1,520 (25.60%)	175 (23.09%)	1,357 (26.41%)	152 (24.84%)	676 (20.92%)	98 (19.18%)
ACR 4	452 (7.61%)	50 (6.60%)	407 (7.92%)	42 (6.86%)	154 (4.76%)	13 (2.54%)
Unknown	24 (0.40%)	10 (1.32%)	9 (0.18%)	3 (0.49%)	9 (0.28%)	5 (0.98%)
**Race:**						
White	1,824 (30.72%)	288 (37.99%)	1,647 (32.05%)	247 (40.36%)	689 (21.32%)	162 (31.70%)
African	34 (0.57%)	3 (0.40%)	29 (0.56%)	3 (0.49%)	29 (0.90%)	3 (0.59%)
Asian	36 (0.61%)	4 (0.53%)	33 (0.64%)	4 (0.65%)	19 (0.59%)	0 (0.00%)
Other Race	34 (0.57%)	6 (0.79%)	34 (0.66%)	6 (0.98%)	22 (0.68%)	0 (0.00%)
Unknown	4,009 (67.53%)	457 (60.29%)	3,396 (66.08%)	352 (57.52%)	2473 (76.52%)	346 (67.71%)
**Gene mutation (Brca 1/2, Chek2, Tp53):**						
Positive	35 (0.59%)	28 (3.69%)	31 (0.60%)	27 (4.41%)	386 (11.94%)	135 (26.42%)
Negative	154 (2.59%)	71 (9.37%)	136 (2.65%)	62 (10.13%)	118 (3.65%)	20 (3.91%)
Unknown	5,748 (96.82%)	659 (86.94%)	4,972 (96.75%)	523 (85.46%)	2,728 (84.41%)	356 (69.67%)
**Menopausal Status:**						
Pre-menopausal	780 (13.14%)	82 (10.82%)	660 (12.84%)	70 (11.44%)	9 (0.28%)	5 (0.98%)
Peri-menopausal & Unknown	3,059 (51.52%)	334 (44.06%)	2,680 (52.15%)	272 (44.44%)	110 (3.40%)	42 (8.22%)
Post- menopausal	2,098 (35.34%)	342 (45.12%)	1,799 (35.01%)	270 (44.12%)	3,113 (96.32%)	464 (90.80%)
**Personal History:**						
Breast Cancer Positive	3,191 (53.75%)	470 (62.01%)	2,812 (54.72%)	380 (62.09%)	3,232 (100.00%)	511 (100.00%)
Breast Cancer Negative	2,746 (46.25%)	288 (37.99%)	2,327 (45.28%)	232 (37.91%)	0 (0.00%)	0 (0.00%)
Ovarian Cancer Positive	82 (1.38%)	27 (3.56%)	76 (1.48%)	26 (4.25%)	26 (0.80%)	8 (1.57%)
Ovarian Cancer Negative	5,855 (98.62%)	731 (96.44%)	5,063 (98.52%)	586 (95.75%)	3,206 (99.20%)	503 (98.43%)
**Menarche Age：**						
<12	97 (1.63%)	3 (0.40%)	91 (1.77%)	3 (0.49%)	19 (0.59%)	4 (0.78%)
12-15	822 (13.85%)	129 (17.02%)	755 (14.69%)	120 (19.61%)	217 (6.71%)	54 (10.57%)
>15	37 (0.62%)	6 (0.79%)	33 (0.64%)	3 (0.49%)	15 (0.46%)	6 (1.17%)
Unknown	4,981 (83.90%)	620 (81.79%)	4,260 (82.90%)	486 (79.41%)	2,981 (92.23%)	447 (87.48%)
**Family History：**						
Breast Cancer Positive	4,321 (72.78%)	691 (91.16%)	3,810 (74.14%)	576 (94.12%)	2,754 (85.21%)	466 (91.19%)
Breast Cancer Negative	1,616 (27.22%)	67 (8.84%)	1,329 (25.86%)	36 (5.88%)	478 (14.79%)	45 (8.81%)
Ovarian Cancer Positive	295 (4.97%)	55 (7.26%)	258 (5.02%)	48 (7.84%)	83 (2.57%)	13 (2.54%)
Ovarian Cancer Negative	5,642 (95.03%)	703 (92.74%)	4,881 (94.98%)	564 (92.16%)	3,149 (97.43%)	498 (97.46%)
**Manufacturer：**						
Hologic Selenia	914 (15.39%)	145 (19.13%)	581 (11.31%)	96 (15.69%)	454 (14.05%)	102 (19.96%)
Lorad Selenia	1,567 (26.39%)	223 (29.42%)	1,266 (24.64%)	170 (27.78%)	813 (25.15%)	139 (27.20%)
Selenia Dimensions	3,429 (57.76%)	381 (50.26%)	3,281 (63.85%)	342 (55.88%)	1,961 (60.67%)	269 (52.64%)
**Time to cancer, years:**						
0-1	137 (2.31%)	137 (18.07%)	102 (1.98%)	102 (16.67%)	121 (3.74%)	121 (23.68%)
1-2	97 (1.63%)	97 (12.80%)	78 (1.52%)	78 (12.75%)	67 (2.07%)	67 (13.11%)
2-3	95 (1.60%)	95 (12.53%)	82 (1.60%)	82 (13.40%)	63 (1.95%)	63 (12.33%)
3-4	85 (1.43%)	85 (11.21%)	73 (1.42%)	73 (11.93%)	59 (1.83%)	59 (11.55%)
4-5	81 (1.36%)	81 (10.69%)	69 (1.34%)	69 (11.27%)	47 (1.45%)	47 (9.20%)
5-6	63 (1.06%)	63 (8.31%)	54 (1.05%)	54 (8.82%)	39 (1.21%)	39 (7.63%)
6-7	69 (1.16%)	69 (9.10%)	53 (1.03%)	53 (8.66%)	40 (1.24%)	40 (7.83%)
7-8	48 (0.81%)	48 (6.33%)	40 (0.78%)	40 (6.54%)	26 (0.80%)	26 (5.09%)
8-9	40 (0.67%)	40 (5.28%)	29 (0.56%)	29 (4.74%)	24 (0.74%)	24 (4.70%)
9-10	43 (0.72%)	43 (5.67%)	32 (0.62%)	32 (5.23%)	25 (0.77%)	25 (4.89%)

Supplementary Table 4. Detailed demographics for two primary screening cohorts of the Inhouse dataset.

	Inhouse Dataset			
	Screening cohort 1*		Screening cohort 2*	
	Total (N=2,445)	Cancer (N=268)	Total (N=2,059)	Cancer (N=214)
**Age:**				
<40	272 (11.12%)	21 (7.84%)	226 (10.98%)	17 (7.94%)
40-50	973 (39.80%)	95 (35.45%)	833 (40.46%)	74 (34.58%)
50-60	780 (31.90%)	78 (29.10%)	667 (32.39%)	67 (31.31%)
60-70	349 (14.27%)	56 (20.90%)	274 (13.31%)	44 (20.56%)
70-80	71 (2.90%)	18 (6.72%)	59 (2.87%)	12 (5.61%)
>80	0 (0.00%)	0 (0.00%)	0 (0.00%)	0 (0.00%)
Unknown	0 (0.00%)	0 (0.00%)	0 (0.00%)	0 (0.00%)
**BI-RADS：**				
BI-RADS 0	29 (1.19%)	1 (0.37%)	32 (1.55%)	4 (1.87%)
BI-RADS 1	634 (25.93%)	57 (21.27%)	634 (30.79%)	57 (26.64%)
BI-RADS 2	1,393 (56.97%)	153 (57.09%)	1,393 (67.65%)	153 (71.50%)
BI-RADS 3	104 (4.25%)	17 (6.34%)	0 (0.00%)	0 (0.00%)
BI-RADS 4	26 (1.06%)	3 (1.12%)	0 (0.00%)	0 (0.00%)
BI-RADS 5	1 (0.04%)	0 (0.00%)	0 (0.00%)	0 (0.00%)
BI-RADS 6	0 (0.00%)	0 (0.00%)	0 (0.00%)	0 (0.00%)
Unknown	17 (0.70%)	17 (6.34%)	0 (0.00%)	0 (0.00%)
**Density:**				
ACR 1	134 (5.48%)	9 (3.36%)	123 (5.97%)	9 (4.21%)
ACR 2	1,313 (53.70%)	143 (53.36%)	1,072 (52.06%)	114 (53.27%)
ACR 3	750 (30.67%)	78 (29.10%)	652 (31.67%)	64 (29.91%)
ACR 4	233 (9.53%)	33 (12.31%)	207 (10.05%)	27 (12.62%)
Unknown	15 (0.61%)	5 (1.87%)	5 (0.24%)	0 (0.00%)
**Race:**				
White	1,019 (41.68%)	127 (47.39%)	904 (43.90%)	113 (52.80%)
African	4 (0.16%)	1 (0.37%)	3 (0.15%)	1 (0.47%)
Asian	13 (0.53%)	2 (0.75%)	11 (0.53%)	2 (0.93%)
Other Race	7 (0.29%)	6 (2.24%)	7 (0.34%)	6 (2.80%)
Unknown	Unknown	132 (49.25%)	1,134 (55.08%)	92 (42.99%)
**Gene:**				
Positive	14 (0.57%)	14 (5.22%)	13 (0.63%)	13 (6.07%)
Negative	46 (1.88%)	31 (11.57%)	36 (1.75%)	27 (12.62%)
Unknown	2,385 (97.55%)	223 (83.21%)	2,010 (97.62%)	174 (81.31%)
**Menopausal Status:**				
Pre-menopausal	399 (16.32%)	41 (15.30%)	333 (16.17%)	35 (16.36%)
Peri-menopausal & Unknown	1,539 (62.94%)	142 (52.99%)	1,319 (64.06%)	115 (53.74%)
Post- menopausal	507 (20.74%)	85 (31.72%)	407 (19.77%)	64 (29.91%)
**Personal History:**				
Breast Cancer Positive	0 (0.00%)	0 (0.00%)	0 (0.00%)	0 (0.00%)
Breast Cancer Negative	2,445 (100.00%)	268 (100.00%)	2,059 (100.00%)	214 (100.00%)
Ovarian Cancer Positive	51 (2.09%)	19 (7.09%)	45 (2.19%)	18 (8.41%)
Ovarian Cancer Negative	2,394 (97.91%)	249 (92.91%)	2,014 (97.81%)	196 (91.59%)
**Menarche Age：**				
<12	73 (2.99%)	0 (0.00%)	68 (3.30%)	0 (0.00%)
12-15	542 (22.17%)	72 (26.87%)	492 (23.90%)	66 (30.84%)
>15	13 (0.53%)	0 (0.00%)	13 (0.63%)	0 (0.00%)
Unknown	1,817 (74.31%)	196 (73.13%)	1,486 (72.17%)	148 (69.16%)
**Family History：**				
Breast Cancer Positive	1,412 (57.75%)	244 (91.04%)	1,214 (58.96%)	205 (95.79%)
Breast Cancer Negative	1,033 (42.25%)	24 (8.96%)	845 (41.04%)	9 (4.21%)
Ovarian Cancer Positive	180 (7.36%)	40 (14.93%)	152 (7.38%)	33 (15.42%)
Ovarian Cancer Negative	2,265 (92.64%)	228 (85.07%)	1,907 (92.62%)	181 (84.58%)
**Manufacturer：**				
Hologic Selenia	427 (17.46%)	53 (19.78%)	270 (13.11%)	36 (16.82%)
Lorad Selenia	695 (28.43%)	86 (32.09%)	555 (26.95%)	70 (32.71%)
Selenia Dimensions	1,301 (53.21%)	122 (45.52%)	1,226 (59.54%)	105 (49.07%)
**Time to cancer, years:**				
0-1	49 (2.00%)	49 (18.28%)	34 (1.65%)	34 (15.89%)
1-2	28 (1.15%)	28 (10.45%)	20 (0.97%)	20 (9.35%)
2-3	32 (1.31%)	32 (11.94%)	25 (1.21%)	25 (11.68%)
3-4	26 (1.06%)	26 (9.70%)	23 (1.12%)	23 (10.75%)
4-5	33 (1.35%)	33 (12.31%)	29 (1.41%)	29 (13.55%)
5-6	23 (0.94%)	23 (8.58%)	21 (1.02%)	21 (9.81%)
6-7	27 (1.10%)	27 (10.07%)	20 (0.97%)	20 (9.35%)
7-8	20 (0.82%)	20 (7.46%)	17 (0.83%)	17 (7.94%)
8-9	15 (0.61%)	15 (5.60%)	14 (0.68%)	14 (6.54%)
9-10	15 (0.61%)	15 (5.60%)	11 (0.53%)	11 (5.14%)

*, Note that, for the sake of maintaining comparability with BCSC methods, we also excluded examinations with a prior history of breast cancer or those outside the age range of 35-74 for both screening cohorts.

Supplementary result of 10-year BC risk prediction

Ablation studies

Supplementary Table 5. Ablation studies on 10-year risk dataset. C-index and AUC results are presented with 95% Confidence Interval. Exam Based: baseline method uses multi-view mammograms (Right/Left; CC/MLO) to predicate breast cancer risk. Multi-Task: multi-task prediction of breast cancer risk and auxiliary tasks (e.g. age, BIRADS, and breast density). Multi-Level: using side-based and exam-based classifiers derived from Side-Specific Prediction Module and Exam-Based Prediction Module. Multi-Time-Point: using multi-time points exams. Use risk factors: input risk factors (race, gene, menarche, menopausal, personal ovarian cancer history, and family history) and prior tumor information and also more auxiliary tasks learning (personal breast cancer history; future cancer subtype and location).

Exam	√	√	√	√	√	√	√
Multi-Task		√		√	√	√	√
Multi-Level			√	√	√	√	√
Multi-Time-Point						√	√
Use risk factors					√		√
10 year C-index	0.64 (0.62-0.66)	0.71 (0.70-0.73)	0.70 (0.68-0.72)	0.73 (0.72-0.75)	0.79 (0.78-0.81)	0.77 (0.75-0.78)	**0.82 (0.81-0.84)**
1-Year AUC	0.74 (0.72-0.76)	0.80 (0.78-0.83)	0.79 (0.77-0.81)	0.84 (0.82-0.86)	0.88 (0.86-0.90)	0.87 (0.85-0.89)	**0.91 (0.89-0.92)**
2-Year AUC	0.70 (0.68-0.72)	0.76 (0.74-0.78)	0.75 (0.73-0.78)	0.80 (0.78-0.82)	0.84 (0.82-0.86)	0.83 (0.81-0.85)	**0.88 (0.86-0.89)**
3-Year AUC	0.67 (0.65-0.69)	0.74 (0.72-0.76)	0.73 (0.71-0.75)	0.78 (0.76-0.80)	0.82 (0.80-0.84)	0.80 (0.78-0.82)	**0.86 (0.84-0.87)**
4-Year AUC	0.65 (0.62-0.67)	0.72 (0.70-0.74)	0.70 (0.68-0.72)	0.75 (0.73-0.77)	0.80 (0.78-0.82)	0.79 (0.77-0.81)	**0.84 (0.82-0.85)**
5-Year AUC	0.62 (0.60-0.64)	0.70 (0.68-0.73)	0.69 (0.67-0.71)	0.73 (0.71-0.75)	0.79 (0.77-0.81)	0.77 (0.75-0.79)	**0.82 (0.81-0.84)**
6-Year AUC	0.61 (0.58-0.63)	0.70 (0.68-0.72)	0.68 (0.66-0.70)	0.72 (0.70-0.74)	0.78 (0.76-0.80)	0.77 (0.75-0.79)	**0.82 (0.80-0.83)**
7-Year AUC	0.59 (0.57-0.61)	0.69 (0.67-0.71)	0.67 (0.65-0.69)	0.71 (0.69-0.73)	0.77 (0.76-0.79)	0.76 (0.75-0.78)	**0.81 (0.80-0.83)**
8-Year AUC	0.57 (0.55-0.59)	0.68 (0.66-0.70)	0.66 (0.64-0.69)	0.70 (0.68-0.72)	0.77 (0.75-0.78)	0.76 (0.74-0.78)	**0.80 (0.79-0.82)**
9-Year AUC	0.55 (0.52-0.57)	0.68 (0.65-0.70)	0.66 (0.64-0.68)	0.69 (0.67-0.71)	0.76 (0.74-0.78)	0.76 (0.74-0.78)	**0.80 (0.78-0.81)**
10-Year AUC	0.53 (0.51-0.56)	0.69 (0.67-0.71)	0.67 (0.65-0.70)	0.70 (0.67-0.72)	0.77 (0.75-0.78)	0.77 (0.75-0.79)	**0.80 (0.78-0.82)**

Supplementary Table 6. Comparison of BCR-MTP method for 10-year risk predictions using different numbers of time point exams on the full inhouse test set. C-index and AUC results are presented with 95% Confidence Interval.

Use risk factors					√	√	√	√
Number of prior reference exams	0	1	3	5	0	1	3	5
10 year C-index	0.73 (0.72-0.75)	0.75 (0.73-0.77)	0.75 (0.73-0.77)	**0.77 (0.75-0.78)**	0.79 (0.78-0.81)	0.80 (0.79-0.82)	0.81 (0.79-0.82)	**0.82 (0.81-0.84)**
1-Year AUC	0.84 (0.82-0.86)	0.86 (0.83-0.88)	0.85 (0.83-0.87)	**0.87 (0.85-0.89)**	0.88 (0.86-0.90)	0.89 (0.88-0.91)	0.90 (0.88-0.92)	**0.91 (0.89-0.92)**
2-Year AUC	0.80 (0.78-0.82)	0.81 (0.79-0.83)	0.81 (0.78-0.83)	**0.83 (0.81-0.85)**	0.84 (0.82-0.86)	0.86 (0.84-0.88)	0.87 (0.85-0.89)	**0.88 (0.86-0.89)**
3-Year AUC	0.78 (0.76-0.80)	0.79 (0.77-0.81)	0.78 (0.76-0.80)	**0.80 (0.78-0.82)**	0.82 (0.80-0.84)	0.84 (0.82-0.86)	0.85 (0.83-0.86)	**0.86 (0.84-0.87)**
4-Year AUC	0.75 (0.73-0.77)	0.77 (0.75-0.79)	0.77 (0.75-0.79)	**0.79 (0.77-0.81)**	0.80 (0.78-0.82)	0.82 (0.80-0.84)	0.83 (0.81-0.84)	**0.84 (0.82-0.85)**
5-Year AUC	0.73 (0.71-0.75)	0.75 (0.73-0.77)	0.76 (0.74-0.78)	**0.77 (0.75-0.79)**	0.79 (0.77-0.81)	0.80 (0.79-0.82)	0.81 (0.79-0.83)	**0.82 (0.81-0.84)**
6-Year AUC	0.72 (0.70-0.74)	0.75 (0.73-0.77)	0.76 (0.74-0.77)	**0.77 (0.75-0.79)**	0.78 (0.76-0.80)	0.80 (0.78-0.81)	0.80 (0.78-0.82)	**0.82 (0.80-0.83)**
7-Year AUC	0.71 (0.69-0.73)	0.74 (0.73-0.76)	0.75 (0.73-0.77)	**0.76 (0.75-0.78)**	0.77 (0.76-0.79)	0.79 (0.77-0.81)	0.79 (0.78-0.81)	**0.81 (0.80-0.83)**
8-Year AUC	0.70 (0.68-0.72)	0.74 (0.72-0.76)	0.75 (0.73-0.77)	**0.76 (0.74-0.78)**	0.77 (0.75-0.78)	0.78 (0.76-0.80)	0.79 (0.77-0.80)	**0.80 (0.79-0.82)**
9-Year AUC	0.69 (0.67-0.71)	0.74 (0.72-0.76)	0.75 (0.73-0.76)	**0.76 (0.74-0.78)**	0.76 (0.74-0.78)	0.77 (0.75-0.79)	0.78 (0.76-0.80)	**0.80 (0.78-0.81)**
10-Year AUC	0.70 (0.67-0.72)	0.74 (0.72-0.76)	0.75 (0.73-0.77)	**0.77 (0.75-0.79)**	0.77 (0.75-0.78)	0.77 (0.75-0.79)	0.78 (0.76-0.80)	**0.80 (0.78-0.82)**

Supplementary Table 7. C-index performance of MTP-BCR models which trained on different training datasets. C-index results are presented with 95% Confidence Interval. Results indicate that the model achieves the best C-index among the full test set and all other sub-cohorts when training on the full dataset, which is better than only training on the corresponding subsets (primary or recurrence) from scratch.

		Full dataset training	Primary dataset training	Recurrence dataset training
MTP-BCR (with risk fators)	Entire test set	**0.82 (0.81-0.84)**	0.77 (0.76-0.79)	0.61 (0.60-0.63)
	Screening cohort 1	**0.74 (0.72-0.76)**	0.70 (0.68-0.71)	0.64 (0.63-0.66)
	Screening cohort 2	**0.73 (0.71-0.75)**	0.70 (0.68-0.72)	0.66 (0.64-0.68)
	Recurrence cohort	**0.72 (0.69-0.74)**	0.68 (0.64-0.72)	0.67 (0.65-0.70)
		Full dataset training	Primary dataset training	Recurrence dataset
MTP-BCR (without risk factors)	Entire test set	**0.77 (0.75-0.78)**	0.72 (0.70-0.73)	0.55 (0.53-0.56)
	Screening cohort 1	**0.65 (0.63-0.67)**	0.62 (0.60-0.64)	0.56 (0.54-0.58)
	Screening cohort 2	**0.64 (0.62-0.67)**	0.61 (0.59-0.63)	0.56 (0.54-0.58)
	Recurrence cohort	**0.63 (0.60-0.66)**	0.59 (0.56-0.61)	0.57 (0.54-0.59)

Results of comparison of 10-year risk prediction on primary BC population

Supplementary Table 8. Comparison of 10-year risk prediction on primary BC (35-74 years old without prior BC) population of the inhouse test set. C-index and AUC results are presented with 95% Confidence Interval. The black fonts represent the performance of the target tasks for which different methods were originally designed. The gray fonts represent the AUC metric of 1- to 10- years BC risk for these methods we explored. Bold: P < 0.05, the AUCs of our methods are significantly higher than all other models for the same time horizon. Traditional-Risk factors: Traditional risk model (BCSC) based on risk factors; Baseline-Risk factors: SVM model based on risk factors; STP-Baseline: Single time point (STP) image-only based baseline DL methods; STP-Detection: STP-based detection DL method; STP-Transformer: STP-based DL risk prediction method, which leverages the transformer to fusion the representation of each view.

	BI-RADS	Traditional-Risk factors	Baseline-Risk factors	STP-Baseline	STP-Detection	STP-Transformer	MTP-BCR (no risk factots)		MTP-BCR	
Risk level	Patient	Patient	Patient	Patient	Patient	Patient	Patient	Breast	Patient	Breast
5-Year C-Index	0.78 (0.76-0.80)	0.63 (0.61-0.65)	0.58 (0.55-0.60)	0.72 (0.69-0.74)	0.77 (0.74-0.79)	0.83 (0.81-0.85)	0.87 (0.85-0.89)	0.86 (0.84-0.88)	0.91 (0.90-0.92)	0.90 (0.88-0.91)
10-Year C-Index	0.76 (0.74-0.78)	0.64 (0.61-0.66)	0.59 (0.57-0.61)	0.70 (0.67-0.72)	0.75 (0.73-0.77)	0.80 (0.78-0.83)	0.86 (0.84-0.87)	0.84 (0.82-0.86)	0.90 (0.88-0.91)	0.88 (0.86-0.89)
1-Year AUC	0.86 (0.83-0.88)	0.62 (0.58-0.64)	0.55 (0.52-0.58)	0.77 (0.75-0.80)	0.80 (0.77-0.83)	0.89 (0.87-0.91)	**0.93 (0.91-0.95)**	0.92 (0.90-0.94)	**0.96 (0.94-0.97)**	0.94 (0.92-0.95)
2-Year AUC	0.84 (0.81-0.86)	0.62 (0.59-0.65)	0.56 (0.53-0.59)	0.75 (0.72-0.78)	0.79 (0.76-0.82)	0.87 (0.85-0.90)	**0.91 (0.89-0.93)**	0.90 (0.88-0.92)	**0.94 (0.92-0.95)**	0.93 (0.91-0.94)
3-Year AUC	0.81 (0.79-0.83)	0.63 (0.61-0.66)	0.57 (0.54-0.60)	0.73 (0.70-0.76)	0.78 (0.76-0.81)	0.85 (0.83-0.88)	**0.89 (0.87-0.91)**	0.89 (0.86-0.91)	**0.92 (0.91-0.94)**	0.91 (0.89-0.93)
4-Year AUC	0.79 (0.77-0.82)	0.64 (0.61-0.67)	0.58 (0.55-0.61)	0.71 (0.68-0.74)	0.78 (0.75-0.80)	0.84 (0.81-0.86)	**0.88 (0.86-0.91)**	0.87 (0.85-0.89)	**0.91 (0.90-0.93)**	0.90 (0.88-0.92)
5-Year AUC	0.77 (0.75-0.79)	0.65 (0.62-0.68)	0.59 (0.57-0.62)	0.69 (0.66-0.72)	0.76 (0.74-0.79)	0.82 (0.79-0.84)	**0.87 (0.85-0.89)**	0.86 (0.84-0.88)	**0.90 (0.89-0.92)**	0.89 (0.87-0.90)
6-Year AUC	0.76 (0.74-0.78)	0.66 (0.63-0.69)	0.61 (0.58-0.64)	0.68 (0.65-0.71)	0.76 (0.74-0.79)	0.81 (0.78-0.84)	**0.87 (0.85-0.89)**	0.86 (0.83-0.88)	**0.90 (0.89-0.92)**	0.88 (0.86-0.90)
7-Year AUC	0.75 (0.73-0.77)	0.67 (0.65-0.70)	0.62 (0.59-0.65)	0.66 (0.63-0.69)	0.76 (0.73-0.78)	0.79 (0.77-0.82)	**0.86 (0.84-0.88)**	0.85 (0.82-0.87)	**0.90 (0.88-0.91)**	0.87 (0.85-0.88)
8-Year AUC	0.74 (0.72-0.76)	0.69 (0.66-0.71)	0.62 (0.60-0.65)	0.63 (0.60-0.66)	0.74 (0.72-0.77)	0.79 (0.76-0.82)	**0.86 (0.84-0.88)**	0.84 (0.82-0.86)	**0.89 (0.87-0.91)**	0.86 (0.84-0.88)
9-Year AUC	0.73 (0.71-0.75)	0.70 (0.67-0.73)	0.63 (0.60-0.67)	0.61 (0.58-0.65)	0.74 (0.71-0.77)	0.77 (0.75-0.81)	**0.86 (0.84-0.88)**	0.84 (0.82-0.86)	**0.89 (0.87-0.90)**	0.85 (0.83-0.86)
10-Year AUC	0.73 (0.70-0.75)	0.71 (0.68-0.74)	0.65 (0.61-0.68)	0.59 (0.56-0.63)	0.74 (0.71-0.76)	0.76 (0.73-0.80)	**0.86 (0.84-0.88)**	0.84 (0.82-0.86)	**0.88 (0.86-0.90)**	0.84 (0.82-0.86)

Supplementary Table 9. Comparison of 10-year risk predictions on primary screening (35-74 years old without prior BC) cohorts. C-index and AUC results are presented with 95% Confidence Interval. The black fonts represent the performance of the target tasks for which different methods were originally designed. The gray fonts represent the AUC metric of 1- to 10-years BC risk for these methods we explored. Bold: $P < 0.05$, the AUCs of our methods are significantly higher than all other models for the same time horizon. Traditional-Risk factors: Traditional risk model (BCSC) based on risk factors; Baseline-Risk factors: SVM model based on risk factors; STP-Baseline: Single time point (STP) image-only based baseline DL methods; STP-Detection: STP-based detection DL method; STP-Transformer: STP-based DL risk prediction method, which leverages the transformer to fusion the representation of each view.

	BI-RADS	Traditional-Risk factors	Baseline-Risk factors	STP-Detection	STP-Transformer	MTP-BCR (no risk factors)		MTP-BCR	
						Patient	Breast	Patient	Breast
Part of Inhouse Screening cohort 1 (Biopsy Negative): only for women who were completely scored across the BCSC model, consisting of 2,445 exams, 49 were followed by cancer diagnosis within one year; 168 diagnosis within five years; 268 diagnosis within ten years.									
5-Year C-Index	0.55 (0.52-0.58)	0.69 (0.65-0.72)	0.61 (0.57-0.65)	0.64 (0.59-0.68)	0.64 (0.60-0.69)	0.72 (0.68-0.76)	0.70 (0.66-0.74)	0.79 (0.76-0.82)	0.77 (0.74-0.81)
10-Year C-Index	0.54 (0.52-0.56)	0.69 (0.66-0.72)	0.64 (0.61-0.67)	0.63 (0.60-0.67)	0.63 (0.59-0.66)	0.71 (0.68-0.74)	0.69 (0.66-0.72)	0.80 (0.77-0.82)	0.76 (0.74-0.79)
1-Year AUC	0.61 (0.55-0.69)	0.70 (0.63-0.76)	0.51 (0.45-0.58)	0.58 (0.48-0.68)	0.76 (0.68-0.83)	0.82 (0.75-0.89)	0.77 (0.67-0.86)	**0.87 (0.81-0.92)**	0.84 (0.76-0.92)
2-Year AUC	0.60 (0.55-0.65)	0.69 (0.64-0.75)	0.56 (0.50-0.62)	0.61 (0.54-0.69)	0.70 (0.63-0.76)	0.75 (0.69-0.81)	0.73 (0.66-0.80)	**0.81 (0.76-0.86)**	0.81 (0.74-0.86)
3-Year AUC	0.57 (0.53-0.61)	0.70 (0.65-0.74)	0.60 (0.54-0.65)	0.64 (0.58-0.69)	0.68 (0.62-0.73)	0.74 (0.69-0.79)	0.73 (0.67-0.78)	**0.80 (0.76-0.85)**	0.79 (0.74-0.84)
4-Year AUC	0.56 (0.52-0.59)	0.70 (0.65-0.74)	0.62 (0.57-0.67)	0.65 (0.59-0.69)	0.65 (0.60-0.70)	0.73 (0.69-0.78)	0.72 (0.66-0.76)	**0.79 (0.76-0.83)**	0.77 (0.73-0.81)
5-Year AUC	0.54 (0.52-0.57)	0.70 (0.65-0.74)	0.63 (0.58-0.67)	0.64 (0.59-0.68)	0.64 (0.59-0.68)	0.73 (0.69-0.77)	0.72 (0.67-0.76)	**0.79 (0.76-0.82)**	0.76 (0.72-0.80)
6-Year AUC	0.54 (0.51-0.56)	0.71 (0.67-0.74)	0.65 (0.61-0.69)	0.65 (0.60-0.69)	0.64 (0.59-0.68)	0.74 (0.70-0.77)	0.72 (0.68-0.76)	**0.80 (0.77-0.83)**	0.76 (0.73-0.80)
7-Year AUC	0.54 (0.52-0.57)	0.71 (0.68-0.75)	0.67 (0.63-0.71)	0.64 (0.60-0.68)	0.63 (0.58-0.67)	0.73 (0.70-0.77)	0.72 (0.68-0.75)	**0.80 (0.77-0.83)**	0.76 (0.72-0.79)
8-Year AUC	0.54 (0.52-0.56)	0.72 (0.69-0.75)	0.67 (0.63-0.71)	0.63 (0.59-0.67)	0.62 (0.58-0.66)	0.74 (0.71-0.78)	0.72 (0.69-0.76)	**0.80 (0.77-0.83)**	0.75 (0.72-0.78)
9-Year AUC	0.53 (0.51-0.55)	0.73 (0.69-0.76)	0.68 (0.64-0.72)	0.63 (0.59-0.67)	0.61 (0.57-0.65)	0.74 (0.71-0.78)	0.73 (0.69-0.76)	**0.80 (0.76-0.82)**	0.73 (0.70-0.76)
10-Year AUC	0.53 (0.51-0.56)	0.74 (0.70-0.77)	0.69 (0.65-0.73)	0.63 (0.58-0.67)	0.60 (0.56-0.64)	0.76 (0.72-0.79)	0.73 (0.70-0.77)	**0.80 (0.77-0.83)**	0.73 (0.70-0.76)
Part of Inhouse Screening cohort 2 (Normal BI-RADS): only for women who were completely scored across the BCSC model, consisting of 2,059 exams, 102 followed by cancer diagnosis within one years; 131 diagnosis within five years; 612 diagnosis within ten years.									
5-Year C-Index	0.51 (0.50-0.53)	0.68 (0.64-0.72)	0.62 (0.58-0.66)	0.64 (0.59-0.68)	0.66 (0.61-0.70)	0.70 (0.66-0.74)	0.70 (0.66-0.75)	0.78 (0.75-0.82)	0.78 (0.74-0.81)
10-Year C-Index	0.51 (0.50-0.52)	0.69 (0.66-0.72)	0.64 (0.61-0.68)	0.64 (0.60-0.67)	0.65 (0.61-0.68)	0.70 (0.67-0.74)	0.69 (0.65-0.73)	0.79 (0.76-0.82)	0.77 (0.74-0.80)
1-Year AUC	0.54 (0.49-0.59)	0.70 (0.63-0.77)	0.52 (0.44-0.60)	0.62 (0.51-0.72)	0.76 (0.69-0.83)	0.79 (0.71-0.87)	0.79 (0.69-0.86)	**0.85 (0.78-0.91)**	0.87 (0.79-0.93)
2-Year AUC	0.52 (0.49-0.56)	0.69 (0.63-0.75)	0.56 (0.49-0.63)	0.62 (0.54-0.70)	0.71 (0.65-0.76)	0.72 (0.65-0.80)	0.74 (0.67-0.81)	**0.80 (0.74-0.85)**	0.82 (0.76-0.88)
3-Year AUC	0.51 (0.49-0.54)	0.70 (0.64-0.74)	0.60 (0.54-0.67)	0.63 (0.57-0.70)	0.68 (0.62-0.73)	0.71 (0.65-0.77)	0.72 (0.65-0.78)	**0.79 (0.74-0.83)**	0.79 (0.74-0.84)
4-Year AUC	0.51 (0.49-0.53)	0.69 (0.65-0.74)	0.62 (0.57-0.67)	0.65 (0.59-0.70)	0.67 (0.61-0.72)	0.71 (0.65-0.76)	0.71 (0.65-0.76)	**0.78 (0.74-0.82)**	0.78 (0.73-0.82)
5-Year AUC	0.51 (0.50-0.53)	0.70 (0.65-0.74)	0.63 (0.58-0.68)	0.64 (0.59-0.69)	0.66 (0.61-0.70)	0.72 (0.67-0.76)	0.71 (0.66-0.76)	**0.78 (0.75-0.82)**	0.77 (0.73-0.80)
6-Year AUC	0.51 (0.50-0.52)	0.71 (0.66-0.75)	0.66 (0.61-0.70)	0.65 (0.60-0.69)	0.67 (0.62-0.71)	0.72 (0.68-0.76)	0.71 (0.67-0.76)	**0.79 (0.76-0.83)**	0.77 (0.73-0.80)
7-Year AUC	0.51 (0.50-0.52)	0.71 (0.67-0.75)	0.66 (0.62-0.71)	0.65 (0.61-0.69)	0.66 (0.62-0.71)	0.72 (0.68-0.76)	0.71 (0.67-0.75)	**0.79 (0.76-0.83)**	0.75 (0.72-0.79)
8-Year AUC	0.51 (0.50-0.52)	0.72 (0.68-0.76)	0.67 (0.62-0.71)	0.64 (0.59-0.68)	0.66 (0.62-0.71)	0.74 (0.70-0.78)	0.72 (0.68-0.76)	**0.79 (0.76-0.83)**	0.75 (0.71-0.78)
9-Year AUC	0.51 (0.50-0.52)	0.73 (0.69-0.76)	0.68 (0.63-0.72)	0.64 (0.60-0.68)	0.65 (0.61-0.70)	0.74 (0.70-0.78)	0.73 (0.69-0.77)	**0.79 (0.76-0.83)**	0.73 (0.70-0.77)
10-Year AUC	0.51 (0.50-0.52)	0.74 (0.70-0.77)	0.69 (0.65-0.74)	0.63 (0.58-0.67)	0.63 (0.58-0.67)	0.75 (0.71-0.79)	0.73 (0.69-0.77)	**0.79 (0.76-0.83)**	0.73 (0.69-0.76)

Supplementary Table 10. The comparison of risk stratification ability on the external CSAW-CC dataset. The summary table for each percentile range (0-10th, 10-50th, 50-90th, 90th and up) was provided, detailing the number of women, number of cancers, percent of cancers accounted for women of each group, and percent of group cancers accounted for all cancers.

			Breast cancer within 7 years			Breast cancer within 60 days			Breast cancer within 60 days-2 years			Breast cancer within 2-7 year		
	All	24694	1826	100.00%	7.39%	524	100.00%	2.12%	267	100.00%	1.10%	1035	100.00%	4.33%
Methods	Groups	N exams	N cancers	Precentage of all N cancers	Precentage of group N exams	N cancers	Precentage of all N cancers	Precentage of group N exams	N cancers	Precentage of all N cancers	Precentage of group N exams	N cancers	Precentage of all N cancers	Precentage of group N exams
STP-Detection	0-10th	2466	76	4.16%	3.08%	1	0.19%	0.04%	14	5.24%	0.58%	61	5.89%	2.49%
	10th-50th	9867	449	24.59%	4.55%	50	9.54%	0.53%	74	27.72%	0.78%	325	31.40%	3.34%
	50th-90th	9891	816	44.69%	8.25%	195	37.21%	2.10%	127	47.57%	1.38%	494	47.73%	5.16%
	90th-100th	2470	485	26.56%	19.64%	278	53.05%	12.28%	52	19.48%	2.55%	155	14.98%	7.24%
STP-Transformer	0-10th	2470	58	3.18%	2.35%	4	0.76%	0.17%	11	4.12%	0.45%	43	4.15%	1.75%
	10th-50th	9877	452	24.75%	4.58%	43	8.21%	0.45%	72	26.97%	0.76%	337	32.56%	3.45%
	50th-90th	9877	791	43.32%	8.01%	155	29.58%	1.68%	134	50.19%	1.45%	502	48.50%	5.24%
	90th-100th	2470	525	28.75%	21.26%	322	61.45%	14.20%	50	18.73%	2.51%	153	14.78%	7.29%
MTP-BCR	0-10th	2470	43	2.35%	1.74%	6	1.15%	0.25%	10	3.75%	0.41%	27	2.61%	1.10%
	10th-50th	9877	350	19.17%	3.54%	41	7.82%	0.43%	58	21.72%	0.61%	251	24.25%	2.57%
	50th-90th	9877	777	42.55%	7.87%	97	18.51%	1.05%	128	47.94%	1.39%	552	53.33%	5.72%
	90th-100th	2470	656	35.93%	26.56%	380	72.52%	17.32%	71	26.59%	3.77%	205	19.81%	10.15%

We also evaluated the ability of risk stratification of different models by dividing the population into specified percentile ranges (0-10th, 10-50th, 50-90th, 90th and up). The results show that our MTP-BCR method effectively stratifies high-risk (>90th percentile) and low-risk (<10th percentile) populations. Women in the highest-risk group accounted for 35.93% of cancers by 7 years, while those in the lowest-risk group accounted for only 2.35%. For breast cancer detection within 60 days, MTP-BCR identified 72.52% of cancers, outperforming other methods. Additionally, it achieved the highest detection rate for interval cancers (26.59%) and consistently outperformed others in stratifying risk for 2-7 year predictions."

Supplementary Table 11. Comparison of 10-year risk predictions with MIRAI on inhosue test set, two screening cohorts, and CSAW-CC dataset. C-index and AUC results are presented with 95% Confidence Interval. "MIRAI-Test (2048×1664)" presents directly testing MIRAI using their released weight with raw mammogram images. It includes a pre-set pre-processing pipeline which will resize the images to a pixel size of 2048×1664. "STP-Transformer" means retraining the MIRAI based on released weight with our pre-processed mammograms (1024×512). We note that when downsampling the mammograms, the performance of the risk model will thus be limited. For further validation on the public CSAW-CC dataset, *MIRAI: directly testing MIRAI using officially provided weight with raw mammogram images. *MTP-BCR: we also combined the MTP-BCR model with a retrained MTP-BCR model based on the large image size of the mammograms for fair comparison. Due to the GPU memory limitations, only two-time points are used in the large image-based model.

Method	C-Index	1-Year AUC	2-Year AUC	3-Year AUC	5-Year AUC	10-Year AUC
Inhouse test set: 6,311 exams, 511 followed by cancer diagnosis within 1 years; 869 diagnosis within 5 years; 1,132 diagnosis within 10 years.						
MIRAI - Test (2048×1664)	0.72 (0.70-0.73)	0.76 (0.74-0.78)	0.74 (0.72-0.76)	0.74 (0.72-0.76)	0.74 (0.72-0.76)	0.75 (0.73-0.77)
STP-Transforme (1024×512)	0.73 (0.72-0.75)	0.84 (0.81-0.86)	0.80 (0.78-0.82)	0.78 (0.76-0.80)	0.74 (0.72-0.76)	0.71 (0.68-0.73)
MTP-BCR (Ours) Patient Level (1024×512)	0.82 (0.81-0.84)	**0.91 (0.89-0.92)**	**0.88 (0.86-0.89)**	**0.86 (0.84-0.87)**	**0.82 (0.81-0.84)**	**0.80 (0.78-0.82)**
MTP-BCR (Ours) Unilateral Breast Level (1024×512)	0.81 (0.79-0.82)	0.89 (0.87-0.91)	0.87 (0.85-0.88)	0.84 (0.83-0.86)	0.81 (0.79-0.82)	0.77 (0.75-0.78)
Inhouse Screening cohort 1 (Normal or biopsy negative): 5,937 exams, 137 followed by cancer diagnosis within 1 years; 495 diagnosis within 5 years; 758 diagnosis within 10 years.						
MIRAI - Test (2048×1664)	0.66 (0.64-0.67)	0.63 (0.58-0.69)	0.64 (0.60-0.68)	0.66 (0.63-0.70)	0.68 (0.66-0.71)	0.71 (0.68-0.73)
STP-Transforme (1024×512)	0.64 (0.62-0.66)	0.65 (0.60-0.70)	0.64 (0.60-0.67)	0.65 (0.62-0.68)	0.66 (0.63-0.68)	0.68 (0.66-0.71)
MTP-BCR (Ours) Patient Level (1024×512)	0.74 (0.72-0.76)	**0.77 (0.73-0.81)**	**0.75 (0.71-0.78)**	**0.75 (0.72-0.77)**	**0.73 (0.71-0.76)**	**0.73 (0.71-0.75)**
MTP-BCR (Ours) Unilateral Breast Level (1024×512)	0.72 (0.71-0.74)	0.76 (0.71-0.81)	0.75 (0.72-0.79)	0.74 (0.71-0.77)	0.72 (0.69-0.74)	0.69 (0.67-0.72)
Inhouse Screening cohort 2 (Normal BI-RADS): 5,139 exams, 102 followed by cancer diagnosis within 1 years; 404 diagnosis within 5 years; 612 diagnosis within 10 yea						
MIRAI - Test (2048×1664)	0.65 (0.63-0.67)	0.63 (0.57-0.68)	0.63 (0.58-0.67)	0.66 (0.62-0.69)	0.68 (0.65-0.70)	0.71 (0.68-0.73)
STP-Transforme (1024×512)	0.62 (0.60-0.65)	0.63 (0.57-0.68)	0.61 (0.57-0.65)	0.63 (0.59-0.66)	0.64 (0.61-0.67)	0.67 (0.65-0.70)
MTP-BCR (Ours) Patient Level (1024×512)	0.73 (0.71-0.75)	**0.74 (0.69-0.79)**	**0.73 (0.69-0.76)**	**0.73 (0.70-0.76)**	**0.72 (0.70-0.75)**	0.73 (0.71-0.76)
MTP-BCR (Ours) Unilateral Breast Level (1024×512)	0.73 (0.71-0.75)	0.77 (0.72-0.81)	0.75 (0.72-0.79)	0.74 (0.71-0.77)	0.72 (0.69-0.74)	0.70 (0.67-0.72)
Method	C-Index	1-Year AUC	2-Year AUC	7-Year AUC		
External CSAW-CC dataset: 24,697 exams, 1,826 followed by cancer diagnosis.						
MIRAI - Test (2048×1664)	0.77 (0.75-0.78)	0.92 (0.91-0.93)	0.86 (0.85-0.88)	0.77 (0.76-0.78)		
STP-Transforme (1024×512)	0.69 (0.68-0.70)	0.86 (0.85-0.88)	0.78 (0.77-0.80)	0.69 (0.68-0.70)		
MTP-BCR (Ours) Patient Level (1024×512)	0.74 (0.73-0.75)	0.88 (0.86-0.90)	0.81 (0.79-0.83)	0.74 (0.72-0.75)		
MTP-BCR (Ours) Unilateral Breast Level (1024×512)	0.74 (0.72-0.75)	0.90 (0.88-0.91)	0.82 (0.81-0.84)	0.74 (0.72-0.75)		
*MTP-BCR (Ours) Patient Level (2048×1664)	0.78 (0.77-0.79)	**0.94 (0.93-0.95)**	0.87 (0.85-0.88)	**0.78 (0.77-0.79)**		
*MTP-BCR (Ours) Unilateral Breast Level (2048×1664)	0.80 (0.79-0.81)	0.95 (0.94-0.96)	0.88 (0.87-0.90)	0.80 (0.78-0.81)		

A — MTP-BCR (Ours) Patient level

	1 year AUC	2 year AUC	3 year AUC	4 year AUC	5 year AUC	6 year AUC	7 year AUC	8 year AUC	9 year AUC	10 year AUC
Excl. BC in 90 days	0.77	0.75	0.75	0.74	0.73	0.74	0.74	0.73	0.73	0.73
Excl. BC in 1 year		0.71	0.73	0.72	0.72	0.73	0.73	0.72	0.72	0.73
Excl. BC in 2 years			0.74	0.72	0.72	0.73	0.73	0.73	0.72	0.73
Excl. BC in 3 years				0.7	0.72	0.73	0.73	0.72	0.72	0.73
Excl. BC in 4 years					0.73	0.75	0.74	0.73	0.73	0.73
Excl. BC in 5 years						0.77	0.75	0.73	0.72	0.73
Excl. BC in 6 years							0.73	0.71	0.71	0.72
Excl. BC in 7 years								0.69	0.69	0.72
Excl. BC in 8 years									0.71	0.73
Excl. BC in 9 years										0.75

B — MTP-BCR (Ours) Breast level

	1 year AUC	2 year AUC	3 year AUC	4 year AUC	5 year AUC	6 year AUC	7 year AUC	8 year AUC	9 year AUC	10 year AUC
Excl. BC in 90 days	0.76	0.75	0.74	0.73	0.72	0.72	0.71	0.7	0.69	0.69
Excl. BC in 1 year		0.74	0.73	0.71	0.71	0.71	0.7	0.69	0.69	0.69
Excl. BC in 2 years			0.71	0.7	0.7	0.7	0.7	0.69	0.68	0.69
Excl. BC in 3 years				0.69	0.7	0.7	0.7	0.69	0.68	0.69
Excl. BC in 4 years					0.71	0.72	0.7	0.69	0.69	0.69
Excl. BC in 5 years						0.73	0.71	0.69	0.69	0.7
Excl. BC in 6 years							0.69	0.68	0.68	0.69
Excl. BC in 7 years								0.68	0.68	0.7
Excl. BC in 8 years									0.69	0.72
Excl. BC in 9 years										0.76

C — STP-Transformer

	1 year AUC	2 year AUC	3 year AUC	4 year AUC	5 year AUC	6 year AUC	7 year AUC	8 year AUC	9 year AUC	10 year AUC
Excl. BC in 90 days	0.64	0.63	0.63	0.62	0.61	0.62	0.62	0.61	0.61	0.61
Excl. BC in 1 year		0.61	0.62	0.6	0.6	0.6	0.61	0.6	0.6	0.6
Excl. BC in 2 years			0.62	0.6	0.6	0.6	0.61	0.6	0.6	0.59
Excl. BC in 3 years				0.57	0.58	0.59	0.6	0.59	0.59	0.59
Excl. BC in 4 years					0.59	0.6	0.61	0.6	0.6	0.59
Excl. BC in 5 years						0.62	0.62	0.6	0.6	0.6
Excl. BC in 6 years							0.62	0.59	0.6	0.59
Excl. BC in 7 years								0.55	0.59	0.59
Excl. BC in 8 years									0.63	0.61
Excl. BC in 9 years										0.59

D — STP-Detection

	1 year AUC	2 year AUC	3 year AUC	4 year AUC	5 year AUC	6 year AUC	7 year AUC	8 year AUC	9 year AUC	10 year AUC
Excl. BC in 90 days	0.59	0.61	0.62	0.63	0.62	0.63	0.63	0.62	0.62	0.63
Excl. BC in 1 year		0.62	0.63	0.64	0.63	0.63	0.63	0.62	0.62	0.63
Excl. BC in 2 years			0.64	0.64	0.62	0.63	0.63	0.62	0.62	0.62
Excl. BC in 3 years				0.63	0.61	0.62	0.62	0.6	0.61	0.61
Excl. BC in 4 years					0.59	0.61	0.61	0.59	0.6	0.6
Excl. BC in 5 years						0.63	0.61	0.59	0.6	0.6
Excl. BC in 6 years							0.59	0.56	0.58	0.59
Excl. BC in 7 years								0.51	0.57	0.59
Excl. BC in 8 years									0.63	0.63
Excl. BC in 9 years										0.63

E — Basesline-Risk factors

	1 year AUC	2 year AUC	3 year AUC	4 year AUC	5 year AUC	6 year AUC	7 year AUC	8 year AUC	9 year AUC	10 year AUC
Excl. BC in 90 days	0.52	0.52	0.53	0.53	0.54	0.55	0.56	0.57	0.58	0.58
Excl. BC in 1 year		0.51	0.54	0.55	0.56	0.57	0.58	0.59	0.59	0.6
Excl. BC in 2 years			0.58	0.57	0.58	0.59	0.6	0.61	0.61	0.62
Excl. BC in 3 years				0.56	0.59	0.6	0.61	0.62	0.63	0.63
Excl. BC in 4 years					0.63	0.63	0.63	0.64	0.65	0.65
Excl. BC in 5 years						0.64	0.64	0.65	0.66	0.66
Excl. BC in 6 years							0.64	0.66	0.66	0.67
Excl. BC in 7 years								0.68	0.67	0.68
Excl. BC in 8 years									0.66	0.67
Excl. BC in 9 years										0.67

F — BI-RADS

	1 year AUC	2 year AUC	3 year AUC	4 year AUC	5 year AUC	6 year AUC	7 year AUC	8 year AUC	9 year AUC	10 year AUC
Excl. BC in 90 days	0.61	0.58	0.56	0.54	0.54	0.53	0.53	0.53	0.52	0.52
Excl. BC in 1 year		0.53	0.51	0.5	0.51	0.51	0.51	0.5	0.5	0.5
Excl. BC in 2 years			0.5	0.49	0.5	0.5	0.5	0.5	0.5	0.5
Excl. BC in 3 years				0.49	0.5	0.5	0.5	0.5	0.5	0.5
Excl. BC in 4 years					0.52	0.51	0.51	0.51	0.5	0.5
Excl. BC in 5 years						0.49	0.51	0.5	0.5	0.5
Excl. BC in 6 years							0.52	0.51	0.5	0.5
Excl. BC in 7 years								0.49	0.49	0.5
Excl. BC in 8 years									0.49	0.5
Excl. BC in 9 years										0.52

Supplementary Fig.4. 1- to 10-year AUC results of risk prediction excluding the breast cancer cases in 90 days-9 years. STP-Baseline: Single time point (STP) image-only based baseline DL methods; STP-Detection: STP-based detection DL method; STP-Transformer: STP-based DL risk prediction method, which leverages the transformer to fusion the representation of each view.

Results of comparison of 10-year risk prediction on recurrence BC group

Supplementary Table 12. Comparison of 10-year risk predictions on recurrence cohort. C-index and AUC results are presented with 95% Confidence Interval. Risk factors only: SVM model based on risk factors; Recurrence SVM: a traditional machine learning (SVM)-based recurrence risk prediction model, leveraging risk factors and prognostic factors. The black fonts represent the performance of the target tasks for which different methods were originally designed. The gray fonts represent the AUC metric of 2- to 10- years BC risk for the methods we explored. Bold: *P*<0.05, the AUCs of our methods are significantly higher than all other models for the same time horizon.

Method	C-Index	1-Year AUC	2-Year AUC	3-Year AUC	5-Year AUC	10-Year AUC
Ipsilateral recurrence + Contralateral recurrence						
BI-RADS	0.58 (0.56-0.60)	0.80 (0.74-0.86)	0.68 (0.64-0.73)	0.62 (0.59-0.66)	0.59 (0.56-0.61)	0.55 (0.52-0.57)
Baseline-Risk factors	0.58 (0.55-0.66)	0.59 (0.52-0.65)	0.55 (0.50-0.61)	0.56 (0.52-0.61)	0.56 (0.52-0.60)	0.58 (0.54-0.62)
Baseline-Recurrence	0.62 (0.59-0.65)	0.64 (0.57-0.71)	0.63 (0.58-0.68)	0.62 (0.57-0.66)	0.61 (0.57-0.64)	0.59 (0.54-0.63)
STP-Detection	0.63 (0.60-0.66)	0.73 (0.67-0.80)	0.69 (0.64-0.74)	0.66 (0.61-0.70)	0.63 (0.60-0.67)	0.62 (0.58-0.66)
MTP-BCR (Patient)	0.72 (0.69-0.74)	0.82 (0.76-0.87)	**0.79 (0.75-0.83)**	**0.76 (0.72-0.79)**	**0.71 (0.68-0.75)**	0.64 (0.59-0.68)
MTP-BCR (Breast)	0.69 (0.66-0.71)	0.78 (0.72-0.83)	0.76 (0.71-0.80)	0.72 (0.68-0.76)	0.68 (0.65-0.71)	0.61 (0.57-0.64)
Contralateral recurrence						
BI-RADS	0.61 (0.57-0.64)	0.83 (0.76-0.90)	0.73 (0.67-0.79)	0.66 (0.61-0.71)	0.61 (0.58-0.65)	0.56 (0.53-0.59)
Baseline-Risk factors	0.57 (0.53-0.61)	0.57 (0.48-0.65)	0.56 (0.49-0.63)	0.57 (0.50-0.62)	0.55 (0.49-0.60)	0.56 (0.51-0.61)
Baseline-Recurrence	0.65 (0.61-0.69)	0.66 (0.56-0.75)	0.67 (0.60-0.73)	0.65 (0.59-0.71)	0.64 (0.59-0.68)	0.61 (0.55-0.66)
MTP-BCR (Patient)	0.79 (0.76-0.82)	0.85 (0.78-0.91)	**0.84 (0.79-0.89)**	**0.82 (0.77-0.86)**	**0.78 (0.74-0.82)**	**0.71 (0.66-0.75)**
MTP-BCR (Breast)	0.68 (0.64-0.71)	0.77 (0.69-0.85)	0.74 (0.67-0.80)	0.71 (0.66-0.77)	0.66 (0.61-0.71)	0.59 (0.54-0.63)
Ipsilateral recurrence						
BI-RADS	0.56 (0.54-0.59)	0.76 (0.66-0.85)	0.63 (0.58-0.69)	0.59 (0.55-0.63)	0.57 (0.54-0.60)	0.53 (0.51-0.56)
Baseline-Risk factors	0.60 (0.56-0.64)	0.63 (0.54-0.72)	0.56 (0.48-0.64)	0.57 (0.51-0.64)	0.58 (0.53-0.63)	0.60 (0.55-0.65)
Baseline-Recurrence	0.62 (0.58-0.66)	0.65 (0.56-0.74)	0.61 (0.54-0.69)	0.60 (0.54-0.66)	0.60 (0.55-0.64)	0.59 (0.53-0.64)
MTP-BCR (Patient)	0.69 (0.65-0.73)	0.79 (0.70-0.87)	**0.76 (0.70-0.82)**	**0.72 (0.66-0.77)**	**0.68 (0.63-0.72)**	0.59 (0.54-0.65)
MTP-BCR (Breast)	0.74 (0.70-0.77)	0.81 (0.74-0.88)	0.80 (0.75-0.85)	0.75 (0.70-0.81)	0.72 (0.68-0.77)	0.67 (0.62-0.71)

Subgroup analysis

Supplementary Table 13. C-index comparison of 10-year risk prediction in different sub-groups of inhsoue dataset. Traditional-Risk factors: Traditional risk model based on risk factors; Baseline-Risk factors: SVM model based on risk factors; STP-Baseline: Single time point (STP) image-only based baseline DL methods; STP-Detection: STP-based detection DL method; STP-Transformer: STP-based DL risk prediction method, which leverages the transformer to fusion the representation of each view.

Subgroup		MTP-BCR	Radiologist BI-RADS	Traditional-Risk factors*	Baseline-Risk factors	STP-Baseline	STP-Detection	STP-Transformer
Age	<40	**0.87 (0.84-0.90)**	0.70 (0.65-0.75)	0.74 (0.68-0.80)	0.65 (0.60-0.70)	0.65 (0.60-0.70)	0.71 (0.66-0.76)	0.76 (0.70-0.81)
	40-60	**0.81 (0.79-0.83)**	0.69 (0.67-0.71)	0.59 (0.57-0.62)	0.55 (0.53-0.57)	0.62 (0.60-0.65)	0.68 (0.66-0.70)	0.72 (0.70-0.74)
	60-80	**0.82 (0.79-0.84)**	0.68 (0.66-0.71)	0.62 (0.57-0.66)	0.56 (0.54-0.59)	0.63 (0.60-0.66)	0.66 (0.63-0.69)	0.71 (0.68-0.74)
	>80	0.78 (0.64-0.90)	0.72 (0.61-0.83)	-	0.51 (0.39-0.63)	0.68 (0.54-0.81)	0.74 (0.62-0.85)	0.78 (0.65-0.89)
BI-RADS	12	**0.73 (0.71-0.75)**	0.50 (0.50-0.50)	0.69 (0.65-0.72)	0.56 (0.53-0.59)	0.54 (0.51-0.56)	0.61 (0.59-0.63)	0.60 (0.58-0.62)
	34	**0.78 (0.73-0.83)**	0.50 (0.50-0.50)	0.54 (0.47-0.60)	0.56 (0.50-0.62)	0.53 (0.47-0.59)	0.65 (0.59-0.70)	0.68 (0.62-0.73)
	36	**0.85 (0.82-0.88)**	0.50 (0.50-0.50)	0.53 (0.47-0.58)	0.55 (0.50-0.60)	0.62 (0.58-0.67)	0.65 (0.61-0.69)	0.77 (0.74-0.81)
Breast Density	No Dense (ACR 1-2)	**0.83 (0.81-0.84)**	0.68 (0.67-0.70)	0.70 (0.67-0.73)	0.58 (0.56-0.60)	0.64 (0.62-0.66)	0.70 (0.68-0.72)	0.73 (0.71-0.75)
	Dense (ACR 3-4)	**0.81 (0.79-0.83)**	0.69 (0.66-0.71)	0.57 (0.53-0.61)	0.57 (0.54-0.60)	0.63 (0.60-0.66)	0.65 (0.62-0.69)	0.72 (0.69-0.75)
Family Cancer History	Ovarian Cancer Positive	**0.80 (0.75-0.85)**	0.64 (0.58-0.71)	0.64 (0.56-0.72)	0.69 (0.63-0.75)	0.51 (0.44-0.58)	0.58 (0.51-0.65)	0.65 (0.58-0.73)
	Ovarian Cancer Negative	**0.82 (0.81-0.84)**	0.69 (0.68-0.71)	0.64 (0.61-0.66)	0.57 (0.55-0.59)	0.65 (0.63-0.67)	0.69 (0.68-0.71)	0.74 (0.72-0.75)
	Breast Cancer Positive	**0.78 (0.76-0.79)**	0.66 (0.65-0.68)	0.59 (0.56-0.62)	0.56 (0.54-0.57)	0.61 (0.59-0.63)	0.67 (0.66-0.69)	0.70 (0.69-0.72)
	Breast Cancer Negative	**0.93 (0.91-0.95)**	0.79 (0.76-0.83)	0.62 (0.57-0.67)	0.59 (0.55-0.63)	0.78 (0.75-0.81)	0.74 (0.69-0.78)	0.83 (0.80-0.86)
Manufacturer	Hologic Selenia	**0.81 (0.79-0.84)**	0.69 (0.66-0.72)	0.68 (0.63-0.73)	0.57 (0.54-0.60)	0.62 (0.59-0.66)	0.68 (0.65-0.72)	0.76 (0.72-0.79)
	Lorad Selenia	**0.84 (0.82-0.86)**	0.70 (0.68-0.72)	0.67 (0.63-0.70)	0.58 (0.56-0.61)	0.67 (0.64-0.70)	0.69 (0.66-0.71)	0.76 (0.74-0.79)
	Selenia Dimensions	**0.79 (0.77-0.81)**	0.63 (0.61-0.66)	0.64 (0.60-0.67)	0.57 (0.54-0.60)	0.60 (0.57-0.62)	0.67 (0.65-0.70)	0.66 (0.64-0.69)
Menopausal Status	Pre-menopausal	**0.87 (0.84-0.89)**	0.68 (0.64-0.72)	0.73 (0.68-0.78)	0.68 (0.63-0.72)	0.63 (0.58-0.67)	0.72 (0.68-0.76)	0.73 (0.69-0.77)
	Peri- & Unknow	**0.80 (0.78-0.82)**	0.70 (0.67-0.72)	0.58 (0.55-0.61)	0.54 (0.52-0.56)	0.63 (0.60-0.66)	0.67 (0.64-0.70)	0.72 (0.69-0.75)
	Post-menopausal	**0.81 (0.79-0.83)**	0.68 (0.66-0.70)	0.61 (0.57-0.65)	0.57 (0.55-0.60)	0.63 (0.60-0.65)	0.67 (0.64-0.70)	0.72 (0.69-0.75)
Cancer Subtype	ER+	**0.82 (0.81-0.84)**	0.68 (0.67-0.70)	0.65 (0.62-0.67)	0.57 (0.55-0.58)	0.65 (0.63-0.66)	0.70 (0.68-0.71)	0.74 (0.72-0.76)
	ER-	**0.84 (0.82-0.86)**	0.69 (0.67-0.71)	0.59 (0.56-0.63)	0.59 (0.56-0.61)	0.64 (0.61-0.67)	0.68 (0.66-0.71)	0.70 (0.68-0.73)
	PR+	**0.81 (0.80-0.83)**	0.68 (0.66-0.69)	0.65 (0.62-0.67)	0.57 (0.55-0.59)	0.64 (0.62-0.66)	0.70 (0.68-0.72)	0.73 (0.71-0.74)
	PR-	**0.85 (0.83-0.86)**	0.70 (0.68-0.72)	0.61 (0.58-0.64)	0.59 (0.57-0.61)	0.66 (0.63-0.68)	0.69 (0.66-0.71)	0.73 (0.71-0.75)
	Her2+	**0.84 (0.82-0.85)**	0.68 (0.65-0.70)	0.59 (0.56-0.62)	0.56 (0.53-0.58)	0.65 (0.62-0.67)	0.71 (0.69-0.73)	0.73 (0.70-0.75)
	Her2-	**0.82 (0.81-0.84)**	0.69 (0.67-0.71)	0.65 (0.62-0.67)	0.59 (0.57-0.60)	0.64 (0.63-0.66)	0.68 (0.67-0.70)	0.73 (0.71-0.75)
	HER2-enriched	**0.86 (0.84-0.88)**	0.69 (0.66-0.71)	0.60 (0.56-0.64)	0.57 (0.54-0.60)	0.66 (0.63-0.68)	0.71 (0.68-0.74)	0.73 (0.70-0.76)
	Lumina	**0.81 (0.80-0.83)**	0.68 (0.66-0.69)	0.65 (0.62-0.67)	0.57 (0.55-0.59)	0.64 (0.62-0.66)	0.70 (0.68-0.72)	0.73 (0.71-0.74)
	Lumina A	**0.83 (0.81-0.84)**	0.68 (0.66-0.70)	0.65 (0.63-0.68)	0.58 (0.56-0.60)	0.65 (0.63-0.67)	0.70 (0.68-0.72)	0.73 (0.71-0.75)
	Lumina B	**0.82 (0.80-0.84)**	0.67 (0.65-0.69)	0.58 (0.54-0.62)	0.55 (0.53-0.58)	0.63 (0.60-0.65)	0.70 (0.67-0.73)	0.70 (0.67-0.72)
	Triple Negative (TN)	**0.82 (0.81-0.84)**	0.68 (0.66-0.71)	0.59 (0.55-0.62)	0.58 (0.56-0.61)	0.63 (0.60-0.65)	0.67 (0.64-0.70)	0.69 (0.66-0.71)

Note that: Note that results of the Traditional-Risk factors model (BCSC) are based on the primary screening population (age range of 35-74 and without prior BC) the full inhouse test set.

Supplementary Table 14. Comparison of risk prediction in different sub-groups of the external CSAW dataset. AUC results are presented with 95% Confidence Interval. Bold: P<0.05, the AUCs of our methods are significantly higher than all other models for the same time horizon. STP-Baseline: Single time point (STP) image-only based baseline DL methods; STP-Detection: STP-based detection DL method; STP-Transformer: STP-based DL risk prediction method, which leverages the transformer to fusion the representation of each view.

Subgroup	Method	C-index	1-Year AUC	2-Year AUC	7 Year-AUC	2-7 Year AUC	3-7 Year-AUC
**Age**							
	STP-Baseline	0.56 (0.54-0.58)	0.67 (0.63-0.72)	0.61 (0.58-0.64)	0.56 (0.54-0.58)	0.53 (0.51-0.55)	0.53 (0.51-0.56)
	STP-Detection	0.65 (0.63-0.66)	0.83 (0.80-0.87)	0.73 (0.70-0.76)	0.65 (0.63-0.67)	0.60 (0.57-0.62)	0.58 (0.56-0.61)
	STP-Transfromer	0.69 (0.67-0.71)	0.88 (0.85-0.91)	0.77 (0.74-0.80)	0.69 (0.67-0.71)	0.63 (0.61-0.66)	0.62 (0.60-0.65)
	**MTP-BCR (Patient level)**	0.73 (0.71-0.75)	0.88 (0.84-0.91)	0.79 (0.76-0.82)	0.73 (0.71-0.75)	0.69 (0.67-0.71)	0.69 (0.66-0.71)
40-55	**MTP-BCR (Breast level)**	0.73 (0.71-0.74)	0.89 (0.85-0.92)	0.80 (0.77-0.83)	0.73 (0.71-0.75)	0.68 (0.66-0.70)	0.68 (0.65-0.70)
	STP-Baseline	0.56 (0.54-0.58)	0.67 (0.63-0.70)	0.63 (0.60-0.66)	0.56 (0.54-0.58)	0.51 (0.48-0.53)	0.51 (0.49-0.53)
	STP-Detection	0.68 (0.67-0.70)	0.82 (0.80-0.84)	0.78 (0.76-0.80)	0.68 (0.67-0.70)	0.61 (0.59-0.63)	0.60 (0.58-0.63)
	STP-Transfromer	0.67 (0.65-0.69)	0.84 (0.82-0.86)	0.78 (0.75-0.80)	0.67 (0.65-0.69)	0.58 (0.56-0.60)	0.58 (0.56-0.60)
	**MTP-BCR (Patient level)**	0.72 (0.71-0.74)	0.87 (0.84-0.89)	0.81 (0.79-0.84)	0.72 (0.71-0.74)	0.65 (0.63-0.67)	0.65 (0.62-0.67)
>55	**MTP-BCR (Breast level)**	0.73 (0.72-0.75)	0.89 (0.87-0.91)	0.84 (0.81-0.86)	0.73 (0.71-0.75)	0.65 (0.63-0.67)	0.65 (0.62-0.67)
**Breast density**							
	STP-Baseline	0.61 (0.59-0.62)	0.71 (0.68-0.74)	0.67 (0.64-0.70)	0.60 (0.59-0.62)	0.55 (0.53-0.57)	0.55 (0.53-0.58)
	STP-Detection	0.69 (0.67-0.70)	0.84 (0.82-0.86)	0.80 (0.77-0.82)	0.69 (0.67-0.70)	0.60 (0.58-0.62)	0.60 (0.57-0.62)
	STP-Transfromer	0.69 (0.67-0.71)	0.87 (0.85-0.89)	0.80 (0.78-0.82)	0.69 (0.67-0.71)	0.60 (0.58-0.62)	0.60 (0.57-0.62)
	**MTP-BCR (Patient level)**	0.75 (0.74-0.77)	0.89 (0.87-0.91)	0.84 (0.82-0.86)	0.75 (0.73-0.77)	0.68 (0.66-0.70)	0.67 (0.65-0.69)
No-dense	**MTP-BCR (Breast level)**	0.75 (0.74-0.77)	0.91 (0.89-0.93)	0.86 (0.84-0.88)	0.75 (0.74-0.77)	0.67 (0.65-0.69)	0.66 (0.64-0.69)
	STP-Baseline	0.56 (0.54-0.58)	0.63 (0.59-0.68)	0.59 (0.55-0.62)	0.56 (0.54-0.58)	0.54 (0.51-0.56)	0.54 (0.52-0.57)
	STP-Detection	0.66 (0.64-0.67)	0.80 (0.77-0.84)	0.72 (0.70-0.75)	0.66 (0.64-0.68)	0.61 (0.59-0.63)	0.60 (0.58-0.63)
	STP-Transfromer	0.69 (0.67-0.71)	0.87 (0.84-0.90)	0.76 (0.73-0.79)	0.69 (0.67-0.71)	0.64 (0.62-0.66)	0.64 (0.61-0.66)
	**MTP-BCR (Patient level)**	0.72 (0.70-0.74)	0.86 (0.82-0.89)	0.77 (0.74-0.80)	0.72 (0.70-0.74)	0.68 (0.66-0.70)	0.68 (0.65-0.70)
Dense	**MTP-BCR (Breast level)**	0.72 (0.70-0.73)	0.87 (0.84-0.90)	0.77 (0.74-0.80)	0.72 (0.70-0.73)	0.67 (0.65-0.70)	0.68 (0.65-0.70)
**Tumer type**							
	STP-Baseline	0.52 (0.48-0.55)	0.60 (0.53-0.67)	0.56 (0.50-0.62)	0.52 (0.48-0.56)	0.49 (0.44-0.54)	0.50 (0.45-0.55)
	STP-Detection	0.72 (0.69-0.75)	0.90 (0.87-0.93)	0.84 (0.80-0.88)	0.72 (0.68-0.75)	0.64 (0.60-0.69)	0.62 (0.57-0.67)
	STP-Transfromer	0.72 (0.69-0.75)	0.87 (0.82-0.91)	0.81 (0.77-0.86)	0.72 (0.69-0.75)	0.66 (0.62-0.70)	0.64 (0.60-0.69)
	**MTP-BCR (Patient level)**	0.75 (0.72-0.78)	0.87 (0.82-0.92)	0.85 (0.81-0.89)	0.75 (0.72-0.78)	0.70 (0.66-0.74)	0.67 (0.63-0.72)
In situ	**MTP-BCR (Breast level)**	0.77 (0.74-0.80)	0.89 (0.84-0.93)	0.87 (0.83-0.91)	0.77 (0.74-0.80)	0.69 (0.65-0.73)	0.67 (0.65-0.68)
	STP-Baseline	0.59 (0.58-0.60)	0.69 (0.67-0.72)	0.64 (0.62-0.66)	0.59 (0.58-0.60)	0.54 (0.53-0.56)	0.55 (0.53-0.57)
	STP-Detection	0.67 (0.65-0.68)	0.82 (0.80-0.84)	0.75 (0.73-0.77)	0.67 (0.65-0.68)	0.60 (0.59-0.62)	0.60 (0.58-0.62)
	STP-Transfromer	0.69 (0.67-0.70)	0.86 (0.85-0.88)	0.78 (0.76-0.80)	0.68 (0.67-0.70)	0.61 (0.60-0.63)	0.61 (0.60-0.63)
	**MTP-BCR (Patient level)**	0.73 (0.72-0.75)	0.88 (0.86-0.90)	0.81 (0.79-0.83)	0.73 (0.72-0.75)	0.68 (0.66-0.69)	0.68 (0.66-0.69)
Invasive	**MTP-BCR (Breast level)**	0.73 (0.72-0.74)	0.90 (0.88-0.92)	0.82 (0.80-0.84)	0.73 (0.72-0.74)	0.67 (0.65-0.68)	0.67 (0.65-0.68)

We further assessed the model's performance across different clinical subgroups based on age, breast density, and tumor type using the CSAW-CC dataset. The results consistently demonstrate the advantages of the MTP-BCR model, with performance either superior or comparable across these subgroups. These findings support our conclusion that modeling breast tissue changes over time enhances the prediction of future breast cancer risk.

Results of adjusted AUC (aAUC)

Supplementary Table 15. Comparison of 10-year risk predictions based on age-adjusted aAUC on inhouse test set. The aAUC results are presented with 95% Confidence Interval. Traditional-Risk factors: Traditional risk model (BCSC) based on risk factors; STP-Baseline: Single time point (STP) image-only based baseline DL methods; STP-Detection: STP-based detection DL method; STP-Transformer: STP-based DL risk prediction method, which leverages the transformer to fusion the representation of each view.

	BI-RADS	Traditional-Risk factors	Baseline-Risk factors	STP-Baseline	STP-Detection	STP-Transformer	MTP-BCR (no risk factors)		MTP-BCR	
Risk level	Patient	Patient	Patient	Patient	Patient	Patient	Patient	Breast	Patient	Breast
**Inhouse test set:** 6,311 exams, 511 followed by cancer diagnosis within 1 years; 869 diagnosis within 5 years; 1,132 diagnosis within 10 years.										
1-Year AUC	0.83 (0.81-0.85)	NA	0.55 (0.54-0.57)	0.74 (0.72-0.76)	0.74 (0.72-0.77)	0.84 (0.81-0.86)	0.87 (0.85-0.89)	0.87 (0.84-0.89)	0.91 (0.89-0.92)	0.89 (0.87-0.90)
2-Year AUC	0.78 (0.76-0.80)	NA	0.55 (0.53-0.57)	0.70 (0.68-0.72)	0.72 (0.70-0.74)	0.80 (0.77-0.82)	0.83 (0.81-0.85)	0.83 (0.81-0.85)	0.88 (0.86-0.89)	0.86 (0.85-0.88)
3-Year AUC	0.74 (0.72-0.76)	NA	0.55 (0.53-0.57)	0.67 (0.65-0.69)	0.71 (0.69-0.73)	0.77 (0.75-0.79)	0.80 (0.78-0.82)	0.80 (0.78-0.82)	0.85 (0.84-0.87)	0.84 (0.82-0.86)
4-Year AUC	0.71 (0.70-0.73)	NA	0.54 (0.52-0.55)	0.64 (0.62-0.66)	0.70 (0.68-0.72)	0.74 (0.72-0.76)	0.78 (0.76-0.80)	0.78 (0.76-0.80)	0.83 (0.82-0.85)	0.82 (0.80-0.84)
5-Year AUC	0.70 (0.68-0.72)	NA	0.54 (0.52-0.56)	0.61 (0.59-0.63)	0.69 (0.67-0.71)	0.72 (0.70-0.75)	0.76 (0.74-0.78)	0.76 (0.74-0.78)	0.82 (0.80-0.83)	0.80 (0.78-0.82)
6-Year AUC	0.68 (0.66-0.70)	NA	0.54 (0.51-0.56)	0.59 (0.57-0.61)	0.68 (0.66-0.70)	0.71 (0.69-0.73)	0.75 (0.73-0.77)	0.74 (0.73-0.76)	0.81 (0.79-0.82)	0.78 (0.77-0.80)
7-Year AUC	0.67 (0.65-0.69)	NA	0.53 (0.51-0.55)	0.57 (0.55-0.59)	0.68 (0.66-0.70)	0.70 (0.68-0.72)	0.74 (0.72-0.76)	0.73 (0.71-0.75)	0.80 (0.78-0.82)	0.77 (0.75-0.79)
8-Year AUC	0.66 (0.64-0.68)	NA	0.53 (0.50-0.55)	0.54 (0.51-0.56)	0.67 (0.64-0.69)	0.69 (0.66-0.71)	0.74 (0.72-0.76)	0.73 (0.71-0.74)	0.78 (0.76-0.80)	0.76 (0.74-0.78)
9-Year AUC	0.65 (0.63-0.68)	NA	0.52 (0.50-0.55)	0.52 (0.49-0.54)	0.66 (0.64-0.69)	0.68 (0.65-0.70)	0.74 (0.72-0.76)	0.73 (0.71-0.74)	0.77 (0.75-0.79)	0.75 (0.73-0.76)
10-Year AUC	0.65 (0.62-0.67)	NA	0.51 (0.48-0.54)	0.50 (0.47-0.52)	0.66 (0.64-0.69)	0.66 (0.64-0.69)	0.75 (0.72-0.77)	0.72 (0.71-0.74)	0.77 (0.74-0.79)	0.74 (0.72-0.76)
**Part of Full Inhouse test set (only for women who were completely scored across the BCSC model)**										
1-Year AUC	0.86 (0.83-0.88)	0.54 (0.51-0.57)	0.53 (0.51-0.55)	0.77 (0.74-0.79)	0.79 (0.76-0.82)	0.89 (0.87-0.91)	0.93 (0.91-0.95)	0.92 (0.90-0.94)	0.95 (0.94-0.97)	0.93 (0.91-0.95)
2-Year AUC	0.84 (0.81-0.86)	0.56 (0.53-0.59)	0.53 (0.51-0.55)	0.74 (0.72-0.77)	0.78 (0.75-0.81)	0.87 (0.84-0.89)	0.91 (0.89-0.93)	0.90 (0.88-0.92)	0.94 (0.92-0.95)	0.92 (0.90-0.94)
3-Year AUC	0.81 (0.79-0.84)	0.56 (0.53-0.59)	0.54 (0.51-0.56)	0.72 (0.69-0.75)	0.77 (0.74-0.80)	0.84 (0.82-0.87)	0.89 (0.87-0.91)	0.88 (0.86-0.91)	0.92 (0.91-0.94)	0.91 (0.89-0.93)
4-Year AUC	0.79 (0.77-0.82)	0.56 (0.53-0.59)	0.54 (0.52-0.56)	0.69 (0.66-0.72)	0.76 (0.73-0.79)	0.82 (0.80-0.85)	0.88 (0.86-0.90)	0.87 (0.85-0.89)	0.91 (0.89-0.93)	0.90 (0.88-0.91)
5-Year AUC	0.77 (0.75-0.80)	0.56 (0.53-0.59)	0.54 (0.51-0.56)	0.67 (0.64-0.70)	0.75 (0.72-0.77)	0.80 (0.77-0.83)	0.87 (0.85-0.89)	0.86 (0.84-0.88)	0.90 (0.88-0.91)	0.88 (0.86-0.90)
6-Year AUC	0.76 (0.73-0.78)	0.56 (0.53-0.60)	0.54 (0.52-0.57)	0.66 (0.63-0.69)	0.74 (0.71-0.77)	0.79 (0.76-0.81)	0.86 (0.84-0.88)	0.85 (0.83-0.87)	0.90 (0.88-0.91)	0.87 (0.85-0.89)
7-Year AUC	0.75 (0.72-0.77)	0.57 (0.54-0.60)	0.54 (0.51-0.57)	0.64 (0.61-0.67)	0.73 (0.70-0.76)	0.78 (0.75-0.80)	0.86 (0.83-0.88)	0.84 (0.82-0.86)	0.89 (0.87-0.91)	0.86 (0.84-0.88)
8-Year AUC	0.74 (0.71-0.76)	0.57 (0.54-0.60)	0.53 (0.50-0.57)	0.61 (0.57-0.64)	0.72 (0.68-0.74)	0.77 (0.74-0.79)	0.85 (0.83-0.87)	0.84 (0.81-0.86)	0.89 (0.87-0.90)	0.85 (0.83-0.87)
9-Year AUC	0.73 (0.69-0.76)	0.58 (0.54-0.61)	0.53 (0.49-0.58)	0.58 (0.55-0.62)	0.72 (0.69-0.75)	0.75 (0.72-0.78)	0.85 (0.82-0.87)	0.83 (0.81-0.86)	0.88 (0.86-0.90)	0.84 (0.82-0.85)
10-Year AUC	0.72 (0.69-0.76)	0.58 (0.54-0.62)	0.53 (0.48-0.59)	0.54 (0.49-0.58)	0.71 (0.68-0.75)	0.74 (0.71-0.77)	0.85 (0.83-0.87)	0.83 (0.81-0.85)	0.87 (0.85-0.89)	0.83 (0.80-0.85)

Supplementary Table 16. Comparison of 10-year risk predictions based on age-adjusted aAUC on two screening cohorts. The aAUC results are presented with 95% Confidence Interval. STP-Detection: STP-based detection method; STP-Transformer: STP-based risk prediction method, which leverages the transformer to fusion the representation of each view.

	BI-RADS	STP-Detection	STP-Transformer	MTP-BCR (no risk factors)		MTP-BCR	
				Patient	Breast	Patient	Breast
Inhouse Screening cohort 1 (Biopsy Negative): 5,937 exams, 137 followed by cancer diagnosis within 1 years; 495 diagnosis within 5 years; 758 diagnosis within 10 years.							
1-Year AUC	0.61 (0.58-0.65)	0.58 (0.53-0.64)	0.64 (0.59-0.69)	0.68 (0.64-0.73)	0.67 (0.61-0.72)	0.76 (0.72-0.80)	0.76 (0.71-0.80)
2-Year AUC	0.58 (0.55-0.60)	0.60 (0.55-0.63)	0.61 (0.57-0.65)	0.66 (0.62-0.70)	0.65 (0.62-0.69)	0.75 (0.71-0.78)	0.75 (0.71-0.78)
3-Year AUC	0.55 (0.53-0.58)	0.60 (0.57-0.64)	0.62 (0.58-0.65)	0.65 (0.62-0.68)	0.65 (0.62-0.68)	0.74 (0.71-0.77)	0.73 (0.70-0.76)
4-Year AUC	0.54 (0.52-0.56)	0.61 (0.58-0.64)	0.61 (0.58-0.64)	0.64 (0.61-0.67)	0.64 (0.61-0.67)	0.73 (0.70-0.75)	0.72 (0.69-0.74)
5-Year AUC	0.54 (0.52-0.56)	0.60 (0.58-0.63)	0.62 (0.59-0.64)	0.63 (0.61-0.66)	0.63 (0.61-0.66)	0.72 (0.70-0.74)	0.71 (0.68-0.73)
6-Year AUC	0.53 (0.51-0.55)	0.60 (0.57-0.62)	0.63 (0.60-0.66)	0.63 (0.61-0.66)	0.63 (0.60-0.65)	0.72 (0.69-0.74)	0.69 (0.67-0.72)
7-Year AUC	0.53 (0.50-0.55)	0.60 (0.57-0.63)	0.63 (0.60-0.65)	0.64 (0.61-0.66)	0.62 (0.60-0.65)	0.72 (0.69-0.74)	0.68 (0.66-0.71)
8-Year AUC	0.53 (0.50-0.55)	0.59 (0.57-0.62)	0.63 (0.60-0.65)	0.64 (0.61-0.66)	0.63 (0.61-0.65)	0.70 (0.68-0.73)	0.68 (0.65-0.70)
9-Year AUC	0.52 (0.49-0.55)	0.59 (0.57-0.62)	0.63 (0.60-0.65)	0.65 (0.62-0.67)	0.63 (0.61-0.65)	0.69 (0.66-0.71)	0.66 (0.64-0.68)
10-Year AUC	0.52 (0.49-0.56)	0.59 (0.56-0.62)	0.62 (0.59-0.65)	0.66 (0.64-0.69)	0.63 (0.60-0.66)	0.69 (0.67-0.72)	0.66 (0.63-0.68)
Inhouse Screening cohort 2 (Normal BI-RADS): 5,139 exams, 102 followed by cancer diagnosis within 1 years; 404 diagnosis within 5 years; 612 diagnosis within 10 years.							
1-Year AUC	0.52 (0.50-0.55)	0.60 (0.54-0.66)	0.61 (0.56-0.67)	0.65 (0.59-0.70)	0.68 (0.62-0.73)	0.74 (0.68-0.79)	0.77 (0.71-0.81)
2-Year AUC	0.51 (0.49-0.53)	0.60 (0.55-0.64)	0.59 (0.54-0.63)	0.64 (0.59-0.68)	0.66 (0.62-0.70)	0.72 (0.69-0.76)	0.75 (0.71-0.78)
3-Year AUC	0.51 (0.49-0.52)	0.61 (0.57-0.64)	0.60 (0.56-0.63)	0.63 (0.60-0.67)	0.65 (0.61-0.68)	0.73 (0.70-0.76)	0.73 (0.69-0.76)
4-Year AUC	0.51 (0.49-0.52)	0.61 (0.58-0.64)	0.59 (0.56-0.63)	0.63 (0.60-0.66)	0.64 (0.61-0.67)	0.71 (0.69-0.74)	0.72 (0.69-0.75)
5-Year AUC	0.51 (0.49-0.52)	0.60 (0.57-0.63)	0.60 (0.57-0.63)	0.63 (0.60-0.65)	0.63 (0.61-0.66)	0.71 (0.69-0.74)	0.71 (0.68-0.73)
6-Year AUC	0.50 (0.48-0.52)	0.60 (0.57-0.63)	0.61 (0.58-0.64)	0.63 (0.60-0.65)	0.63 (0.60-0.65)	0.71 (0.69-0.73)	0.70 (0.67-0.72)
7-Year AUC	0.50 (0.47-0.53)	0.60 (0.57-0.64)	0.61 (0.58-0.64)	0.63 (0.60-0.65)	0.62 (0.60-0.65)	0.71 (0.68-0.74)	0.69 (0.67-0.71)
8-Year AUC	0.50 (0.47-0.53)	0.60 (0.57-0.63)	0.62 (0.58-0.64)	0.64 (0.61-0.67)	0.62 (0.60-0.65)	0.70 (0.68-0.73)	0.68 (0.66-0.71)
9-Year AUC	0.50 (0.47-0.53)	0.60 (0.57-0.63)	0.62 (0.58-0.64)	0.65 (0.62-0.68)	0.63 (0.60-0.66)	0.69 (0.66-0.72)	0.66 (0.64-0.69)
10-Year AUC	0.50 (0.46-0.54)	0.59 (0.56-0.62)	0.61 (0.58-0.64)	0.66 (0.63-0.69)	0.63 (0.60-0.66)	0.70 (0.66-0.73)	0.66 (0.63-0.69)

Supplementary Table 17. Comparison of 10-year risk predictions based on age-adjusted aAUC on two primary screening (35-74 years old without prior BC) cohorts. The aAUC results are presented with 95% Confidence Interval. Traditional-Risk factors: Traditional risk model (BCSC) based on risk factors; STP-Detection: STP-based detection method; STP-Transformer: STP-based risk prediction method, which leverages the transformer to fusion the representation of each view.

		Traditional -	STP-	STP-	MTP-BCR (no risk factors)		MTP-BCR	
	BI-RADS	Risk factors	Detection	Transformer	Patient	Breast	Patient	Breast
Part of Inhouse Screening cohort 1 (Biopsy Negative): only for women who were completely scored across the BCSC model, consisting of 2,445 exams, 49 were followed by cancer diagnosis within one year; 168 diagnosis within five years; 268 diagnosis within ten years.								
1-Year AUC	0.61 (0.55-0.69)	0.58 (0.51-0.66)	0.56 (0.46-0.66)	0.75 (0.67-0.82)	0.81 (0.73-0.88)	0.76 (0.67-0.85)	0.86 (0.80-0.91)	0.84 (0.75-0.90)
2-Year AUC	0.60 (0.55-0.65)	0.63 (0.57-0.69)	0.59 (0.52-0.66)	0.70 (0.64-0.76)	0.76 (0.69-0.81)	0.73 (0.65-0.79)	0.81 (0.76-0.86)	0.81 (0.74-0.85)
3-Year AUC	0.57 (0.53-0.61)	0.64 (0.59-0.69)	0.62 (0.56-0.67)	0.67 (0.61-0.72)	0.74 (0.69-0.79)	0.72 (0.66-0.77)	0.80 (0.76-0.84)	0.79 (0.74-0.83)
4-Year AUC	0.56 (0.52-0.60)	0.63 (0.58-0.68)	0.62 (0.57-0.67)	0.66 (0.60-0.71)	0.73 (0.68-0.77)	0.71 (0.66-0.75)	0.79 (0.75-0.83)	0.77 (0.72-0.81)
5-Year AUC	0.54 (0.51-0.58)	0.63 (0.59-0.67)	0.61 (0.57-0.66)	0.64 (0.60-0.69)	0.72 (0.68-0.76)	0.71 (0.67-0.75)	0.78 (0.74-0.81)	0.76 (0.72-0.79)
6-Year AUC	0.54 (0.50-0.57)	0.63 (0.60-0.67)	0.62 (0.57-0.66)	0.64 (0.59-0.68)	0.72 (0.69-0.76)	0.71 (0.67-0.75)	0.79 (0.75-0.82)	0.75 (0.72-0.79)
7-Year AUC	0.54 (0.51-0.58)	0.64 (0.59-0.68)	0.61 (0.57-0.65)	0.63 (0.59-0.67)	0.72 (0.68-0.76)	0.71 (0.67-0.74)	0.80 (0.77-0.83)	0.74 (0.71-0.78)
8-Year AUC	0.54 (0.49-0.58)	0.64 (0.60-0.68)	0.60 (0.56-0.64)	0.63 (0.58-0.67)	0.73 (0.69-0.76)	0.71 (0.68-0.74)	0.79 (0.76-0.82)	0.73 (0.70-0.76)
9-Year AUC	0.53 (0.48-0.58)	0.65 (0.61-0.69)	0.60 (0.56-0.64)	0.62 (0.57-0.67)	0.73 (0.69-0.77)	0.71 (0.68-0.75)	0.78 (0.75-0.82)	0.71 (0.68-0.75)
10-Year AUC	0.53 (0.47-0.59)	0.65 (0.60-0.69)	0.60 (0.55-0.65)	0.60 (0.55-0.65)	0.74 (0.70-0.78)	0.72 (0.68-0.75)	0.78 (0.74-0.82)	0.71 (0.67-0.74)
Part of Inhouse Screening cohort 2 (Normal BI-RADS): only for women who were completely scored across the BCSC model, consisting of 2,059 exams, 102 followed by cancer diagnosis within one years; 131 diagnosis within five years; 612 diagnosis within ten years.								
1-Year AUC	0.54 (0.49-0.59)	0.62 (0.54-0.70)	0.60 (0.50-0.70)	0.73 (0.64-0.80)	0.78 (0.68-0.86)	0.78 (0.69-0.86)	0.85 (0.77-0.91)	0.86 (0.77-0.92)
2-Year AUC	0.52 (0.49-0.56)	0.66 (0.59-0.72)	0.60 (0.53-0.68)	0.68 (0.61-0.75)	0.72 (0.64-0.79)	0.74 (0.65-0.81)	0.79 (0.73-0.85)	0.82 (0.76-0.88)
3-Year AUC	0.51 (0.48-0.54)	0.67 (0.62-0.72)	0.62 (0.55-0.68)	0.64 (0.58-0.70)	0.71 (0.65-0.77)	0.72 (0.66-0.78)	0.78 (0.73-0.83)	0.79 (0.74-0.84)
4-Year AUC	0.51 (0.48-0.54)	0.65 (0.60-0.70)	0.63 (0.57-0.68)	0.63 (0.57-0.69)	0.70 (0.65-0.75)	0.70 (0.65-0.75)	0.78 (0.73-0.82)	0.78 (0.73-0.82)
5-Year AUC	0.51 (0.48-0.54)	0.64 (0.59-0.69)	0.62 (0.57-0.67)	0.61 (0.56-0.66)	0.71 (0.66-0.76)	0.70 (0.65-0.75)	0.77 (0.73-0.81)	0.76 (0.72-0.80)
6-Year AUC	0.50 (0.47-0.54)	0.64 (0.60-0.69)	0.62 (0.57-0.66)	0.62 (0.57-0.67)	0.71 (0.67-0.75)	0.70 (0.65-0.74)	0.78 (0.74-0.82)	0.75 (0.71-0.79)
7-Year AUC	0.50 (0.47-0.54)	0.65 (0.61-0.69)	0.63 (0.58-0.67)	0.62 (0.57-0.67)	0.71 (0.66-0.75)	0.69 (0.65-0.73)	0.78 (0.74-0.82)	0.74 (0.70-0.77)
8-Year AUC	0.50 (0.46-0.55)	0.64 (0.60-0.68)	0.61 (0.56-0.66)	0.61 (0.56-0.66)	0.72 (0.67-0.75)	0.70 (0.67-0.74)	0.78 (0.74-0.82)	0.72 (0.69-0.76)
9-Year AUC	0.50 (0.45-0.55)	0.65 (0.60-0.70)	0.61 (0.56-0.66)	0.61 (0.56-0.66)	0.72 (0.68-0.77)	0.71 (0.67-0.75)	0.77 (0.73-0.81)	0.71 (0.67-0.75)
10-Year AUC	0.50 (0.42-0.56)	0.64 (0.59-0.69)	0.59 (0.54-0.65)	0.57 (0.50-0.63)	0.72 (0.67-0.76)	0.70 (0.67-0.74)	0.76 (0.72-0.80)	0.69 (0.65-0.73)

e

Supplementary Table 18. Comparison of 10-year risk predictions based on age-adjusted aAUC on recurrence cohort. The aAUC results are presented with 95% Confidence Interval. Baseline-Recurrence SVM: a traditional machine learning (SVM)-based recurrence risk prediction model, leveraging risk factors and prognostic factors. STP-Detection: STP-based detection method.

	BI-RADS	Baseline-Recurrence	STP-Detection	MTP-BCR Patient	MTP-BCR Breast
Inhouse recurrence cohort: 3,232 exams, 121 followed by cancer diagnosis within one year; 357 diagnosis within five years; 511 diagnosis within ten years.					
1-Year AUC	0.80 (0.74-0.85)	0.65 (0.58-0.71)	0.72 (0.65-0.78)	0.81 (0.76-0.86)	0.77 (0.72-0.83)
2-Year AUC	0.68 (0.63-0.73)	0.63 (0.58-0.68)	0.68 (0.62-0.73)	0.79 (0.75-0.83)	0.75 (0.70-0.79)
3-Year AUC	0.62 (0.59-0.66)	0.62 (0.57-0.66)	0.65 (0.60-0.69)	0.76 (0.72-0.79)	0.72 (0.68-0.75)
4-Year AUC	0.59 (0.56-0.63)	0.61 (0.57-0.65)	0.63 (0.59-0.67)	0.73 (0.69-0.76)	0.69 (0.65-0.73)
5-Year AUC	0.59 (0.56-0.62)	0.61 (0.57-0.65)	0.62 (0.58-0.66)	0.71 (0.68-0.74)	0.68 (0.64-0.71)
6-Year AUC	0.58 (0.54-0.61)	0.61 (0.57-0.64)	0.61 (0.57-0.65)	0.69 (0.66-0.72)	0.66 (0.63-0.69)
7-Year AUC	0.56 (0.53-0.59)	0.61 (0.57-0.64)	0.62 (0.58-0.65)	0.67 (0.64-0.71)	0.64 (0.61-0.67)
8-Year AUC	0.55 (0.52-0.59)	0.60 (0.56-0.64)	0.61 (0.57-0.65)	0.64 (0.61-0.68)	0.62 (0.59-0.65)
9-Year AUC	0.55 (0.51-0.59)	0.59 (0.55-0.64)	0.60 (0.56-0.64)	0.62 (0.58-0.66)	0.61 (0.57-0.64)
10-Year AUC	0.54 (0.50-0.59)	0.59 (0.54-0.63)	0.60 (0.56-0.64)	0.62 (0.58-0.66)	0.60 (0.56-0.63)

Results of comparison of 10-year risk prediction on patient-level evaluation:

Supplementary Table 19. Comparison of 10-year risk predictions on inhouse test set, three cohorts, and CSAW-CC dataset using patient-level evaluation (one exam per patient).

Method	C-Index	1-Year AUC	2-Year AUC	3-Year AUC	5-Year AUC	10-Year AUC
**Inhouse test set: 1,356 patients, 192 followed by cancer diagnosis within 1 years; 249 diagnosis within 5 years; 290 diagnosis within 10 years.**						
BI-RADS	0.76 (0.74-0.79)	0.84 (0.80-0.87)	0.80 (0.77-0.83)	0.78 (0.75-0.82)	0.76 (0.73-0.80)	0.72 (0.68-0.75)
STP-Baseline	0.71 (0.68-0.74)	0.78 (0.74-0.81)	0.74 (0.71-0.78)	0.71 (0.67-0.75)	0.67 (0.62-0.71)	0.61 (0.56-0.66)
Baseline-Risk factors	0.60 (0.57-0.62)	0.61 (0.58-0.65)	0.59 (0.56-0.63)	0.59 (0.56-0.63)	0.56 (0.52-0.61)	0.55 (0.50-0.61)
STP-Detection	0.69 (0.65-0.72)	0.71 (0.66-0.76)	0.70 (0.66-0.74)	0.70 (0.66-0.74)	0.69 (0.65-0.73)	0.70 (0.65-0.74)
STP-Transformer	0.77 (0.74-0.80)	0.84 (0.80-0.87)	0.80 (0.76-0.83)	0.79 (0.75-0.82)	0.77 (0.73-0.80)	0.75 (0.70-0.79)
MTP-BCR (Ours)	**0.85 (0.82-0.87)**	**0.92 (0.89-0.94)**	**0.88 (0.85-0.91)**	**0.87 (0.84-0.90)**	**0.84 (0.80-0.87)**	**0.81 (0.77-0.85)**
**Inhouse Screening cohort 1 (Normal or biopsy negative): 1,236 patients, 57 followed by cancer diagnosis within 1 years; 121 diagnosis within 5 years; 161 diagnosis within 10 years.**						
BI-RADS	0.58 (0.55-0.61)	0.66 (0.59-0.72)	0.60 (0.56-0.65)	0.59 (0.55-0.63)	0.57 (0.54-0.61)	0.55 (0.51-0.58)
STP-Baseline	0.60 (0.55-0.65)	0.66 (0.59-0.73)	0.61 (0.55-0.67)	0.59 (0.53-0.64)	0.55 (0.48-0.61)	0.52 (0.46-0.58)
Baseline-Risk factors	0.55 (0.50-0.60)	0.55 (0.47-0.62)	0.50 (0.44-0.57)	0.53 (0.47-0.59)	0.52 (0.46-0.58)	0.54 (0.48-0.60)
STP-Detection	0.59 (0.54-0.63)	0.59 (0.50-0.67)	0.59 (0.52-0.65)	0.61 (0.55-0.67)	0.58 (0.52-0.64)	0.61 (0.55-0.67)
STP-Transformer	0.62 (0.57-0.67)	0.68 (0.59-0.76)	0.64 (0.57-0.70)	0.64 (0.58-0.70)	0.61 (0.54-0.67)	0.61 (0.54-0.66)
MTP-BCR (Ours)	**0.73 (0.69-0.77)**	**0.78 (0.71-0.85)**	**0.76 (0.70-0.81)**	**0.76 (0.71-0.81)**	**0.71 (0.66-0.76)**	**0.71 (0.65-0.76)**
**Inhouse Screening cohort 2 (Normal BI-RADS): 1,157 patients, 47 followed by cancer diagnosis within 1 years; 101 diagnosis within 5 years; 141 diagnosis within 10 years.**						
BI-RADS	0.52 (0.50-0.54)	0.54 (0.50-0.58)	0.52 (0.50-0.55)	0.52 (0.50-0.55)	0.52 (0.50-0.54)	0.51 (0.49-0.53)
STP-Baseline	0.57 (0.52-0.62)	0.61 (0.52-0.70)	0.60 (0.52-0.67)	0.57 (0.50-0.64)	0.52 (0.44-0.59)	0.50 (0.43-0.57)
Baseline-Risk factors	0.56 (0.50-0.62)	0.51 (0.42-0.61)	0.52 (0.44-0.60)	0.55 (0.47-0.62)	0.53 (0.45-0.60)	0.59 (0.52-0.66)
STP-Detection	0.60 (0.55-0.65)	0.64 (0.55-0.73)	0.62 (0.55-0.69)	0.61 (0.55-0.67)	0.59 (0.53-0.65)	0.62 (0.55-0.69)
STP-Transformer	0.59 (0.54-0.64)	0.64 (0.55-0.72)	0.61 (0.54-0.68)	0.61 (0.54-0.67)	0.58 (0.51-0.64)	0.59 (0.52-0.66)
MTP-BCR (Ours)	**0.70 (0.66-0.75)**	**0.73 (0.65-0.80)**	**0.71 (0.64-0.77)**	**0.72 (0.65-0.77)**	**0.67 (0.61-0.73)**	**0.75 (0.69-0.80)**
**Inhouse recurrence cohort: 614 patients, 20 followed by cancer diagnosis within 1 years; 63 diagnosis within 5 years; 92 diagnosis within 10 years.**						
BI-RADS	0.61 (0.57-0.66)	0.84 (0.73-0.94)	0.71 (0.62-0.80)	0.65 (0.58-0.73)	0.62 (0.57-0.67)	0.56 (0.51-0.61)
Baseline-Risk factors	0.57 (0.50-0.63)	0.60 (0.48-0.72)	0.53 (0.43-0.64)	0.54 (0.45-0.63)	0.54 (0.46-0.63)	0.58 (0.48-0.67)
Baseline-Recurrence	0.61 (0.55-0.68)	0.66 (0.53-0.79)	0.60 (0.49-0.70)	0.60 (0.50-0.69)	0.59 (0.51-0.67)	0.57 (0.47-0.67)
STP-Detection	0.58 (0.51-0.65)	0.71 (0.57-0.83)	0.67 (0.57-0.78)	0.65 (0.56-0.75)	0.56 (0.47-0.64)	0.54 (0.43-0.64)
MTP-BCR (Ours)	0.70 (0.63-0.76)	0.81 (0.69-0.91)	0.77 (0.68-0.86)	0.74 (0.66-0.83)	0.68 (0.60-0.76)	0.59 (0.49-0.69)
**Method**	**C-Index**	**1-Year AUC**	**2-Year AUC**	**7-Year AUC**		
**CSAW-CC dataset: 8723 patients, 873 followed by cancer diagnosis.**						
STP-Baseline	0.60 (0.58-0.62)	0.69 (0.66-0.72)	0.65 (0.62-0.68)	0.60 (0.58-0.62)		
STP-Detection	0.70 (0.68-0.71)	0.84 (0.81-0.86)	0.77 (0.75-0.79)	0.69 (0.67-0.71)		
STP-Transformer	0.71 (0.69-0.73)	0.86 (0.84-0.89)	0.79 (0.77-0.81)	0.71 (0.69-0.73)		
MTP-BCR (Ours)	**0.75 (0.74-0.77)**	**0.88 (0.86-0.91)**	**0.83 (0.81-0.85)**	**0.75 (0.73-0.77)**		
